# Opposite causal effects of birthweight on myocardial infarction and atrial fibrillation and the distinct mediating pathways: a Mendelian randomization study

**DOI:** 10.1186/s12933-023-02062-5

**Published:** 2023-12-12

**Authors:** Lijie Kong, Yiying Wang, Chaojie Ye, Chun Dou, Dong Liu, Min Xu, Jie Zheng, Ruizhi Zheng, Yu Xu, Mian Li, Zhiyun Zhao, Jieli Lu, Yuhong Chen, Weiqing Wang, Ruixin Liu, Yufang Bi, Tiange Wang, Guang Ning

**Affiliations:** 1 Department of Endocrine and Metabolic Diseases, Shanghai Institute of Endocrine and Metabolic Diseases, Ruijin Hospital, Shanghai Jiao Tong University School of Medicine, 197 Rujin 2nd Road, Shanghai, China; 2 Shanghai National Clinical Research Center for Metabolic Diseases, Key Laboratory for Endocrine and Metabolic Diseases of the National Health Commission of the PR China, Shanghai Key Laboratory for Endocrine Tumor, Ruijin Hospital, Shanghai Jiao Tong University School of Medicine, Shanghai, China

**Keywords:** Atrial fibrillation, Birthweight, Body composition, Cardiometabolic factors, Mediation, Mendelian randomization, Myocardial Infarction, Socioeconomic status

## Abstract

**Background:**

Previous observational studies have documented an inverse association of birthweight with myocardial infarction (MI) but a positive association with atrial fibrillation (AF). However, the causality of these associations and the underlying mediating pathways remain unclear. We aimed to investigate the causal effects of birthweight, incorporating both fetal and maternal genetic effects, on MI and AF, and identify potential mediators in their respective pathways.

**Methods:**

We performed Mendelian randomization (MR) analyses using genome-wide association study summary statistics for birthweight (N = 297,356 for own birthweight and 210,248 for offspring birthweight), MI (N_case_=61,000, N_control_=577,000), AF (N_case_=60,620, N_control_=970,216), and 52 candidate mediators (N = 13,848-1,295,946). Two-step MR was employed to identify and assess the mediation proportion of potential mediators in the associations of birthweight with MI and AF, respectively. As a complement, we replicated analyses for fetal-specific birthweight and maternal-specific birthweight.

**Results:**

Genetically determined each 1-SD lower birthweight was associated with a 40% (95% CI: 1.22–1.60) higher risk of MI, whereas each 1-SD higher birthweight was causally associated with a 29% (95% CI: 1.16–1.44) higher risk of AF. Cardiometabolic factors, including lipids and lipoproteins, glucose and insulin, blood pressure, and fatty acids, each mediated 4.09-23.71% of the total effect of birthweight on MI, followed by body composition and strength traits (i.e., appendicular lean mass, height, and grip strength) and socioeconomic indicators (i.e., education and household income), with the mediation proportion for each factor ranging from 8.08 to 16.80%. By contrast, appendicular lean mass, height, waist circumference, childhood obesity, and body mass index each mediated 15.03-45.12% of the total effect of birthweight on AF. Both fetal-specific birthweight and maternal-specific birthweight were inversely associated with MI, while only fetal-specific birthweight was positively associated with AF. Psychological well-being and lifestyle factors conferred no mediating effect in either association.

**Conclusions:**

Cardiometabolic factors mainly mediated the association between lower birthweight and MI, while body composition and strength traits mediated the association between higher birthweight and AF. These findings provide novel evidence for the distinct pathogenesis of MI and AF and advocate adopting a life-course approach to improving fetal development and subsequent causal mediators to mitigate the prevalence and burden of cardiovascular diseases.

**Supplementary Information:**

The online version contains supplementary material available at 10.1186/s12933-023-02062-5.

## Introduction

Myocardial infarction (MI) and atrial fibrillation (AF) are major contributors to the global burden of cardiovascular diseases, with their prevalence nearly doubling in the past three decades [1–3]. MI is primarily caused by coronary artery embolism, whereas AF mainly results from structural changes induced by ischemia and over-inflammation [2, 4, 5]. The mechanisms of atherosclerosis, as well as over-oxidation and inflammation, are mainly responsible for atherosclerotic plaque instability, rupture, and acute coronary events [6, 7]. Furthermore, patients with recurrent AF exhibited elevated inflammation marker levels, while the persistence of AF was found to exert a detrimental influence on atrial over-inflammation, oxidation, and fibrosis through epigenetic regulation [8, 9].

The Developmental Origins of Health and Disease (DOHaD) hypothesis proposes that early-life intrauterine exposures that affect fetal growth shape individual differences in the pathogenesis of cardiometabolic disease in later life [10–13]. Birthweight is a widely used indicator reflecting fetal intrauterine growth and maternal environment that influences fetal growth [14]. Previous cohort and Mendelian randomization (MR) studies have documented an inverse association of birthweight with MI but a positive association with AF [15–17]. Both maternal and fetal genomes contribute to birthweight [14, 18], however, few studies have distinguished the impacts of birthweight determined by maternal genetic effect and fetal genetic effect, respectively, on MI or AF, which could offer detailed insights into the origins and the biological regulations of the relationships of birthweight with these outcomes [14].

Another uncertainty is whether there exist different mediating pathways that may explain the observed opposite effects of birthweight on MI and AF. Experimental evidence suggests that an adverse intrauterine environment permanently influences the cellular proliferation, key organ differentiation, and biophysical profiles of cardiovascular and metabolic systems [13]. Genetic epidemiological evidence has associated birthweight with body composition, physical strength, and metabolic traits in later life [14, 19], some of which have been suggested as risk factors for MI or AF. Therefore, exploring the respective mediators in the associations of birthweight with MI and AF could improve the understanding of etiology and facilitate additional opportunities for the prevention and intervention of MI and AF.

To fill the knowledge gap, we applied MR approaches to discern the causal effects of birthweight, incorporating both fetal and maternal genetic effects, on MI and AF, with a particular interest in identifying mediators in respective association pathways. MR approaches apply genetic variants as instrumental variables (IVs) to infer causality between related traits [20]. For mediation analysis, the two-step MR strategy is sensitive to causal mediating effects and less susceptible to measurement error [21].

## Methods

### Study design

**Fig. 1:**
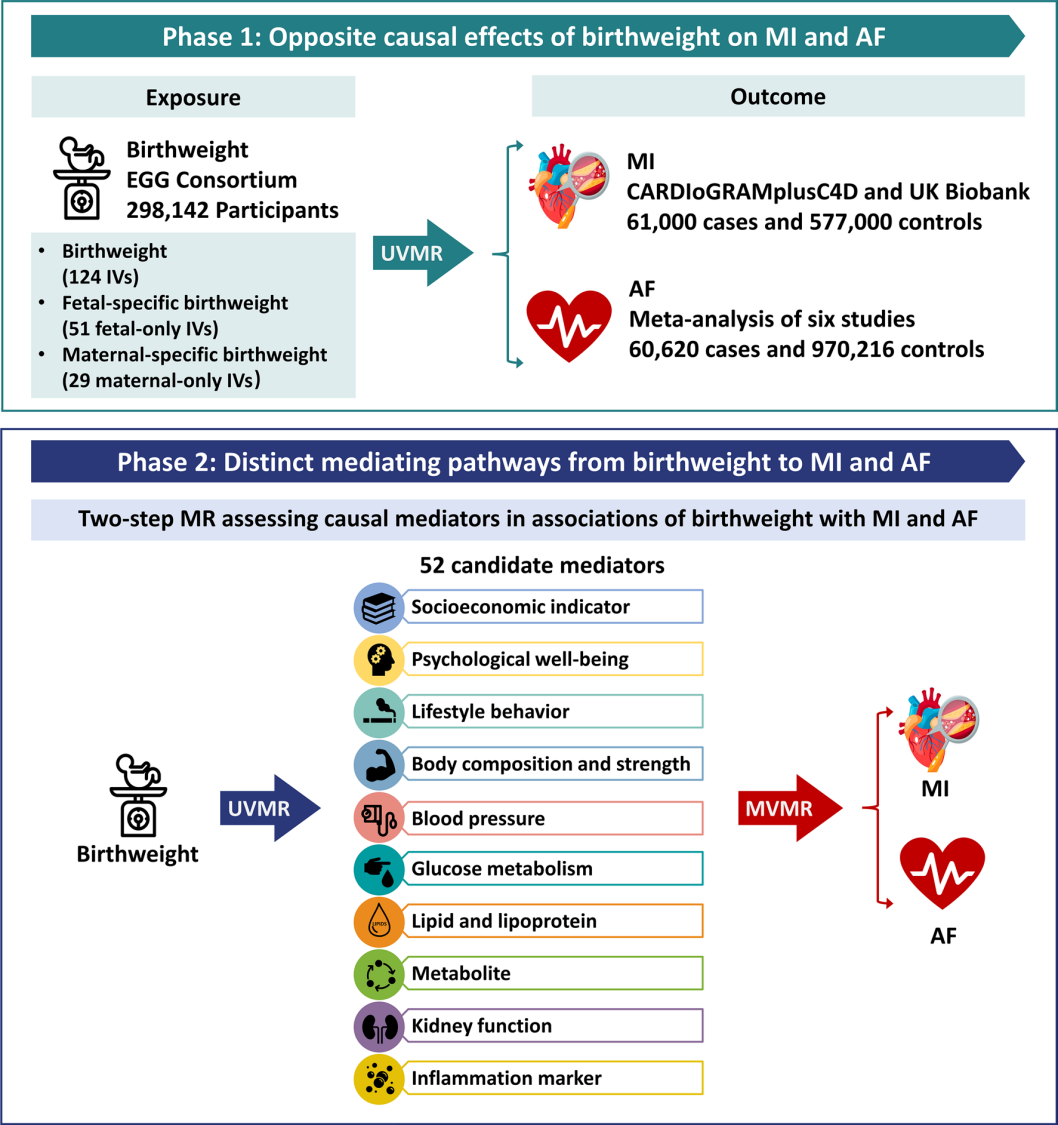
Overview of the MR study design.  This MR study consisted of two analysis phases. In Phase 1, the causal associations of birthweight with MI and AF were estimated using UVMR, and the MR estimates showed that birthweight had opposite effects on MI and AF. In Phase 2, two-step MR was applied to screen for 52 candidate mediators that may lie in the pathways between birthweight and MI or AF and quantify the mediation proportions for qualified mediators.  *AF*  atrial fibrillation, *EGG*  Early Growth Genetics, *IV*  instrumental variable; *MR*  Mendelian randomization; *MI*  myocardial infarction, *MVMR*  multivariable Mendelian randomization, *UVMR*  univariable Mendelian randomization

This MR study consisted of two analysis phases (Fig. 1). In Phase 1, we assessed the causal effects of birthweight on MI and AF using univariable MR (UVMR) and identified that birthweight had opposite effects on MI and AF. In Phase 2, we screened for 52 candidate mediators that may lie in the pathways between birthweight and MI or AF and calculated the mediation proportion of each qualified mediator using two-step MR. This MR study was reported following the Strengthening the Reporting of Observational Studies in Epidemiology using Mendelian Randomization (STROBE-MR) guidelines (Additional file 1: Table S1) [22]. We adopted multiple methods to meet the three core assumptions of MR as follows [20, 23]. First, the IVs are strongly associated with the exposure (i.e., birthweight) in UVMR analysis or at least one of the multiple exposures in multivariable MR (MVMR) analysis. Second, the IVs are independent of confounders of the relationship between exposure and outcome (i.e., MI or AF). Third, the IVs influence the outcome only through exposure but not any direct or indirect pathways.

This study used publicly available summary statistics of genome-wide association study (GWAS) derived from reliable consortia or studies of predominantly European-descent individuals. Ethical approval and informed consent can be found in the corresponding GWAS publications cited in this work.

### Data sources for and selection of genetic instrumental variable

The data sources for the exposure, mediators, and outcomes used in this study are shown in Table 1.

**Table 1:** GWAS data sources of MR study

Phenotype	PMID or GWAS ID	Sample size (overall or case/control)	Ancestry	Unit	Consortium or cohort study
Exposure					
Birthweight	31043758	298,142	European	1-SD (about 500 g)	EGG
Outcome					
MI	33532862	61,000/577,000	European	Event	CARDIoGRAMplusC4D and UK Biobank
AF	30061737	60,620/970,216	European	Event	HUNT, deCODE, MGI, DiscovEHR, UK Biobank, and AFGen Consortium
Candidate mediator					
Socioeconomic indicators (n = 4)					
Education	30038396	1,131,881	European	1-SD	SSGAC
Household income	29846171	397,751	European	1-SD	UK Biobank
Occupational attainment	34613391	248,847	European	1-point in skill level^a^	
Townsend deprivation index	29846171	462,464	European	1-SD	
Psychological well-being indicators (n=4)					
Positive affect	30643256	410,603	European	Z score	Meta
Life satisfaction	30643256	80,852	European	Z score	
Neuroticism	30643256	582,989	European	Z score	
Depressive symptoms	30643256	1,295,946	European	Z score	
Lifestyle behaviours (n=7)					
Cigarettes smoked per day	30643251	337,334	European	1-SD	GSCAN
Alcoholic drinks per week	30643251	335,394	European	1-SD	
Coffee consumption	31046077	375,833	European	1% change	UK Biobank
Long sleep (≥9 h per night)	30846698	34,184/305,742	European	log-transformed odds	
Short sleep (<7 h per night)	30846698	106,192/305,742	European	log-transformed odds	
Chronotype	29846171	413,343	European	1-SD	
MPA (device-measured)	29899525	91,084	European	1-SD	
Body composition and strength (n=7)					
Childhood obesity	22484627	5,530/8,318	European	log-transformed odds	EGG
BMI	25673413	322,154	European	1-SD	GIANT
WC	25673412	210,088	European	1-SD	
WHR	25673412	210,088	European	1-SD	
Height	25282103	253,288	European	1-SD	
Appendicular lean mass	33097823	450,243	European	1-SD	UK Biobank
Grip strength	29846171	461,026	European	1-SD	
Cardiometabolic traits (n = 30)					
Blood pressure					
Hypertension	29846171	42,857/162,837	European	log-transformed odds	FinnGen
SBP	30224653	757,601	European	1 mmHg	ICBP
DBP	30224653	757,601	European	1 mmHg	
Glucose metabolism					
Type 2 diabetes	30054458	62,892/596,424	European	log-transformed odds	DIAGRAM, GERA, and UK Biobank
Fasting glucose	34059833	200,622	European	mmol/L	MAGIC
2-h glucose	34059833	63,396	European	log-transformed mmol/L	
HbA1c	34059833	146,806	European	%	
Fasting insulin	34059833	151,013	European	log-transformed pmol/L	
Lipid and lipoprotein					
Total cholesterol	24097068	188,577	Mixed	1-SD	GLGC
HDL-C	24097068	188,577	Mixed	1-SD	
LDL-C	24097068	188,577	Mixed	1-SD	
Triglycerides	24097068	188,577	Mixed	1-SD	
ApoA-I	27005778	24,925	European	1-SD	Meta
ApoB	27005778	24,925	European	1-SD	
Metabolites					
Omega-3 fatty acids	met-d-Omega_3	114,999	European	1-SD	UK Biobank
Omega-6 fatty acids	met-d-Omega_6	114,999	European	1-SD	
DHA	met-d-DHA	114,999	European	1-SD	
Linoleic acid	met-d-LA	114,999	European	1-SD	
Isoleucine	met-d-Ile	115,075	European	1-SD	
Leucine	met-d-Leu	115,074	European	1-SD	
Valine	met-d-Val	115,048	European	1-SD	
Phenylalanine	met-d-Phe	115,025	European	1-SD	
Tyrosine	met-d-Tyr	115,075	European	1-SD	
Alanine	met-d-Ala	115,074	European	1-SD	
Glutamine	met-d-Gln	114,750	European	1-SD	
Glycine	met-d-Gly	114,972	European	1-SD	
Kidney function					
CKD	31152163	41,395/439,303	European	log-transformed odds	CKDGen
eGFR	31152163	567,460	European	1-SD	
UACR	31511532	547,361	European	1-SD	
Inflammation marker					
CRP	35459240	575,531	European	1-SD	Meta

^a^Occupational attainment was recoded as numeric ordinal variables ranging from one to nine with higher values reflecting greater and more complex occupational attainment

*AF* atrial fibrillation, *AFGen *atrial Fibrillation Genetics, *ApoA-I *Apolipoprotein A-I, *ApoB *Apolipoprotein B, *BMI *body mass index, *CARDIoGRAMplusC4D*  Coronary ARtery Disease Genome wide Replication and Meta-analysis plus The Coronary Artery Disease Genetics, *CKD*  Chronic kidney disease, *CKDGen*  CKD Genetics, *CRP*  C-reactive protein, *DBP*  diastolic blood pressure, *DHA*  docosahexaenoic acid, *DIAGRAM*  DIAbetes Genetics Replication and Meta-analysis, *eGFR*  estimated glomerular filtration rate, *EGG*  Early Growth Genetics; *eMERGE*  Electronic Medical Records and Genomics, *GIANT*  Genetic Investigation of ANthropometric Traits, *GLGC*  Global Lipids Genetics Consortium, *GWAS*  genome-wide association study, *HbA1c*  Glycated hemoglobin, *HDL-C*  high-density lipoprotein cholesterol, *HUNT*  Nord-Trøndelag Health Study, *ICBP*  International Consortium of Blood Pressure, *LDL-C*  low-density lipoprotein cholesterol, *MAGIC*  Meta-Analyses of Glucose and Insulin-related traits Consortium, *MGI*  Michigan Genomics Initiative, *MR*  Mendelian randomization, *SBP*  systolic blood pressure, *SD*  standard deviation, *SSGAC*  Social Science Genetic Association Consortium, *WC*  Waist circumference; *WHR *waist-to-hip ratio, *UACR * urinary albumin-to-creatinine ratio

### Birthweight

We extracted GWAS summary statistics of birthweight (unadjusted overall genetic variants on own birthweight), fetal-specific birthweight (derived from the association of fetal genetic variants on own birthweight with adjustment for maternal genetic effects using the weighted linear model), and maternal-specific birthweight (derived from the association of maternal genetic variants on offspring birthweight with adjustment for fetal genetic effects using the weighted linear model) from currently the largest GWAS meta-analysis from the Early Growth Genetics (EGG) Consortium and UK Biobank (n = 297,356 for own birthweight and n = 210,248 for offspring birthweight) [14]. Information on birthweight was collected by measurement at birth, obstetric records, medical registries, or self-reports. In the EGG consortium, individuals reporting being part of multiple births or with a gestational age < 37 weeks were excluded. In the UK Biobank, individuals reporting being part of multiple births or those likely pre-term births with birthweight < 2.5 kg or > 4.5 kg were excluded. Birthweight measures were Z-score transformed separately in men and women for analysis and adjusted for study-specific covariates and gestational duration (where available).

The original GWAS study identified 205 autosomal single nucleotide polymorphisms (SNP) independently associated with birthweight (P < 6.60 × 10^–9^ and r^2^ < 0.10) [14], including 63 SNPs with fetal-only effects and 31 SNPs with maternal-only effects (Additional file 1: Table S2). We conducted a more stringent linkage disequilibrium clumping with a cut-off of r^2^ < 0.001 within a 10,000 kb window using the 1000 genomes reference panel to select independent genetic variants. Finally, we used 124 birthweight IVs to estimate the causal effects of birthweight on candidate mediators, MI, and AF. As a complement, we replicated analyses using 51 fetal-only effects IVs to determine fetal-specific birthweight and 29 maternal-only effects IVs to determine maternal-specific birthweight, in order to provide an in-depth understanding of the biological regulation of birthweight and the origins of the relationships of birthweight with MI or AF.

### Candidate mediators

Based on literature reviews of observational and MR studies, we focused on 52 candidate mediators that may lie in the pathways from birthweight to MI or AF (detailed evidence see Additional file 1: Table S3). These candidate mediators are prevalent and can be modified, prevented, or treated, making them valuable targets for the prevention or intervention of cardiovascular diseases, and they have available genetic instruments derived from GWASs. Candidate mediators included socioeconomic indicators, psychological well-being indicators, lifestyle behaviors, body composition and strength traits, and cardiometabolic traits representing blood pressure, glucose metabolism, lipids and lipoproteins, metabolites, kidney function, and inflammation (Table 1).

We screened for mediators of the causal associations of birthweight with MI and AF according to the following criteria: [1] birthweight should be causally associated with the mediator; [2] the mediator should have a direct causal effect on the outcome independently of birthweight; and [3] the total effect of birthweight on the outcome and the mediating effect of the mediator should be in the same direction. Eventually, 17 and 5 mediators met all criteria and were included in the mediation analyses to quantify their mediating proportions in the causal associations of birthweight with MI and AF, respectively. We replicated the mediation analyses for the associations of fetal- and maternal-specific birthweight with MI or AF, respectively.

In UVMR analyses, genetic IVs for each candidate mediator were at a genome-wide significant level (P < 5 × 10^–8^) and independent of each other (LD r^2^ < 0.001 within 10,000 kb). In MVMR analyses, genetic IVs were the combination of SNPs, which were genome-wide significant (P < 5 × 10^–8^) in either the GWAS of birthweight or the GWAS of each candidate mediator and were independent of each other (LD r^2^ < 0.001 within 10,000 kb). Where SNPs for the exposures were not available in the GWAS summary statistics of MI or AF, we used proxies of SNPs with r^2^ > 0.8 as substitutes by using the LDproxy search on the online platform LDlink (https://ldlink.nci.nih.gov/).

### Outcomes

Genetic associations of IVs with MI were obtained from the largest GWAS meta-analysis by the CARDIoGRAMplusC4D Consortium and UK Biobank, including 61,000 MI cases and 577,000 controls of European ancestry [24]. MI cases were defined as positive for International Classification of Diseases version-10 (ICD10) codes I21, I22, I23, and I25.2, which included MI and complications following acute MI, and physician-diagnosed or self-reported MI.

Genetic associations with AF were extracted from the largest GWAS meta-analysis of 60,620 AF cases and 970,216 controls of European ancestry [25], comprising data from the Nord-Trøndelag Health Study (HUNT), deCODE, the Michigan Genomics Initiative (MGI), DiscovEHR, UK Biobank, and the AFGen Consortium. AF cases were defined by clinically diagnosed atrial fibrillation or flutter (ICD-10 code I48 and ICD-9 code 427.3).

### Statistical analysis

#### UVMR and MVMR analyses

We used the inverse-variance weighted (IVW) method as the primary analysis in UVMR and the multivariable inverse variance weighted (MV-IVW) as the main analysis in MVMR. The IVW method combines the Wald ratio estimates of every SNP in the set of IVs into one causal estimate using the random-effects meta-analysis [26]. The MR causal estimates were provided as odds ratios (OR) with 95% confidence intervals (CI) for binary outcomes and β coefficients with 95% CIs for continuous outcomes.

#### Total effect of birthweight on MI and AF

We performed UVMR to assess the total causal effects of birthweight on MI and AF and conducted leave-one-out analyses to evaluate the influence of individual variants on these associations.

#### Mediation MR analysis

We applied two-step MR analyses to assess whether an intermediate factor could mediate the association between birthweight and MI and AF, respectively [19, 27]. The first step was to estimate the causal effect (β1) of genetically determined birthweight on each potential mediator using UVMR and the second step was to estimate the causal effect (β2) of each potential mediator on MI or AF with adjustment for birthweight using MVMR. Where there was evidence that birthweight influenced the mediator, which in turn influenced the outcome, we utilized the “product of coefficients” method to assess the mediation effect (β1 × β2) of birthweight on MI or AF via each mediator. The mediation proportion of each mediator was calculated by dividing the mediation effect by the total effect. Standard errors for the mediation effects were derived by using the delta method. The negative mediation proportion was truncated at a lower limit of 0%, as this is the lowest threshold to determine a mediation proportion.

### MR sensitivity analysis

For UVMR analyses, we performed the weighted median, simple mode, weighted mode, MR-Egger, and MR pleiotropy residual sum and outlier (PRESSO) methods as sensitivity analyses to evaluate the robustness of the IVW estimates under different assumptions. For MVMR analyses, we performed the MVMR Egger method to validate the robustness of the MV-IVW results. The weighted median method selects the median MR estimate as the causal estimate and provides a consistent causal estimate if over 50% of the weight in the analysis is derived from valid IVs [28]. The simple mode and weighted mode methods cluster the SNPs based on the similarity of causal effects and estimate the causal effect based on the largest cluster of SNPs [29]. The MR-Egger method, which allows the intercept to be freely estimated as an indicator of pleiotropy, is used to identify and adjust for the potential directional pleiotropic bias but has limited precision [30]. The presence of pleiotropy was also assessed by applying the MR-PRESSO method, which detects and corrects for any outlying SNP reflecting likely pleiotropic biases for MR causal estimates and evaluates whether the exclusion of the outlying SNPs influences the causal estimates [31]. We calculated the F-statistics to evaluate the validity of the IVs and applied Cochran’s Q statistic to assess the heterogeneity and the intercept of MR Egger to test for the pleiotropy of the IVW estimates. Given that multiple candidate mediators were tested in the analyses, we used the Benjamini-Hochberg method for false discovery rate (FDR) correction to account for multiple testing. IVW estimates with P < 0.05 and FDR q-value < 0.05 and supported by at least one sensitivity analysis were considered causal evidence, whereas the IVW estimates with P < 0.05 but FDR q-value ≥ 0.05 or not supported by sensitivity analysis were considered suggestive causal evidence.

All MR analyses were conducted with the R packages ‘*TwoSampleMR*’, ‘*MRPRESSO*’, and ‘*MendelianRandomization*’, and the FDR q-values were estimated using the R package ‘*fdrtool*’ in R software (version 4.1.1; R Foundation for Statistical Computing, Vienna, Austria).

## Results

### Causal associations of birthweight with MI and AF

In UVMR, genetically determined each 1-standard deviation (SD) lower birthweight was causally associated with an increased risk of MI (IVW-OR: 1.40; 95% CI: 1.22–1.60), which was supported by at least three sensitivity analyses (Table 2). Genetically determined lower fetal-specific birthweight (IVW-OR: 1.31; 95% CI: 1.13–1.51) and maternal-specific birthweight (1.44; 1.10–1.88) were also associated with an increased risk of MI. By contrast, genetically determined each 1-SD higher birthweight was causally associated with an increased risk of AF (IVW-OR: 1.29; 95% CI: 1.16–1.44), supported by all five sensitivity analyses. Genetically determined fetal-specific birthweight was positively associated with AF (IVW-OR: 1.18; 95% CI: 1.02–1.36), whereas maternal-specific birthweight was not significantly associated with AF.

**Table 2:** MR estimates for the causal associations of birthweight with MI and AF

Exposure	Method	No. of SNPs or outliers	F-statistic	OR (95% CI)^a^	P value
Association between lower birthweight and MI					
Birthweight	IVW	121	52	1.40 (1.22–1.60)	2.31E−06
	MR Egger			1.17 (0.84–1.61)	3.53E−01
	Simple mode			1.34 (0.98–1.83)	6.62E−02
	Weighted median			1.27 (1.13–1.43)	8.60E−05
	Weighted mode			1.22 (1.03–1.44)	2.46E−02
	MR-PRESSO	7		1.30 (1.17–1.43)	1.16E−06
Fetal-specific birthweight	IVW	50	33	1.31 (1.13–1.51)	3.12E−04
	MR Egger			1.25 (0.84–1.85)	2.72E−01
	Simple mode			1.35 (0.99–1.85)	6.43E−02
	Weighted median			1.30 (1.12–1.52)	7.62E−04
	Weighted mode			1.30 (1.05–1.61)	1.89E−02
	MR-PRESSO	3		1.34 (1.19–1.51)	1.82E−05
Maternal-specific birthweight	IVW	29	32	1.44 (1.10–1.88)	7.42E−03
	MR Egger			1.24 (0.52–2.95)	6.36E−01
	Simple mode			1.34 (0.92–1.95)	1.38E−01
	Weighted median			1.22 (1.01–1.48)	3.63E−02
	Weighted mode			1.37 (0.94–1.99)	1.16E−01
	MR-PRESSO	4		1.36 (1.16–1.59)	8.81E−04
Association between higher birthweight and AF					
Birthweight	IVW	123	52	1.29 (1.16–1.44)	4.50E−06
	MR Egger			1.48 (1.15–1.91)	3.16E−03
	Simple mode			1.29 (1.01–1.63)	4.30E−02
	Weighted median			1.29 (1.16–1.44)	5.18E−06
	Weighted mode			1.24 (1.06–1.45)	7.57E−03
	MR-PRESSO	5		1.27 (1.15–1.40)	2.65E−06
Fetal-specific birthweight	IVW	51	33	1.18 (1.02–1.36)	2.31E−02
	MR Egger			1.48 (1.01–2.17)	5.05E−02
	Simple mode			1.23 (0.96–1.59)	1.06E−01
	Weighted median			1.28 (1.12–1.47)	3.52E−04
	Weighted mode			1.25 (1.03–1.50)	2.48E−02
	MR-PRESSO	3		1.22 (1.09–1.37)	1.58E−03
Maternal-specific birthweight	IVW	29	32	1.13 (0.96–1.32)	1.31E−01
	MR Egger			1.37 (0.85–2.21)	2.11E−01
	Simple mode			1.37 (1.02–1.86)	4.91E−02
	Weighted median			1.07 (0.92–1.25)	3.78E−01
	Weighted mode			1.02 (0.80–1.29)	8.95E−01
	MR-PRESSO	2		1.12 (0.99–1.27)	8.90E−02

^a^ORs (95% CIs) represent the risk for MI associated with each 1-SD lower birthweight or the risk for AF associated with each 1-SD higher birthweight

*AF*  atrial fibrillation, *CI*  confidence interval, *IVW*  inverse variance weighted, *MI*  myocardial infarction, *MR*  Mendelian randomization, *MR-PRESSO*  Mendelian randomization pleiotropy residual sum and outlier, *No*  number, *OR*  odds ratio, *SNP*  single nucleotide polymorphism

There was potential heterogeneity between IVs, but the instrumental validity test suggested sufficient instrument strength (all F-statistics ≥ 32), and no horizontal pleiotropy was detected (all P_intercept_≥0.07; Additional file 1: Table S4). Leave-one-out analysis revealed that no single SNP significantly altered the causal associations (Additional file 1: Figs. S1, S2).

### Causal associations of birthweight with candidate mediators

**Fig. 2:**
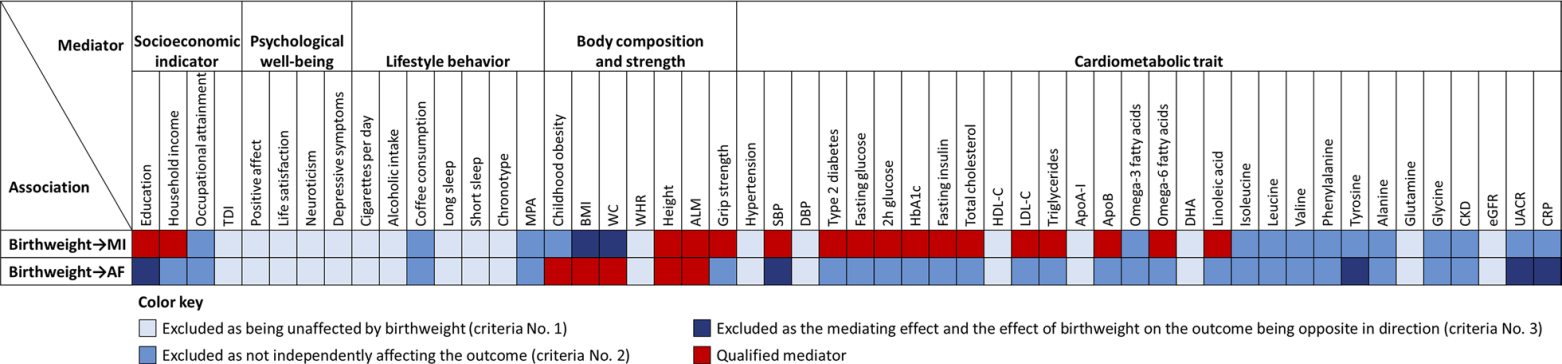
Selection process for mediators in the causal associations of birthweight with MI and AF. Three criteria were applied to screen for the mediators in the causal associations of birthweight with MI and AF: [1] birthweight is causally associated with the mediator; [2] the mediator has a direct causal effect on the outcome independently of birthweight; and [3] the total effect of birthweight on the outcome and the mediating effect of the mediator are in the same direction. Eventually, a total of 17 and 5 qualified mediators met all criteria and were included in the mediation analyses to quantify their individual mediating proportions in the causal associations of birthweight with MI and AF, respectively. The full MR estimates are shown in Additional file 1: Table S5, Table S7.  *AF*  atrial fibrillation, *ApoA-I*  Apolipoprotein A-I, *ApoB*  Apolipoprotein B, *BMI*  body mass index, *CI*  confidence interval, *CKD*  Chronic kidney disease, *CRP*  C-reactive protein, *DBP*  diastolic blood pressure; *DHA*  docosahexaenoic acid, *eGFR*  estimated glomerular filtration rate, *HbA1c * glycated hemoglobin, *HDL-C*  high-density lipoprotein cholesterol, *LDL-C*  low-density lipoprotein cholesterol, *MI*  myocardial infarction, *SBP*  systolic blood pressure; *TDI * Townsend deprivation index, *UACR*  urinary albumin-to-creatinine ratio; *WC*  Waist circumference, *WHR*  waist-to-hip ratio

Of 52 candidate mediators, 17 and 5 candidates met all screening criteria and were qualified as mediators between birthweight and MI and AF, respectively (Fig. 2). In UVMR, higher birthweight was causally associated with higher socioeconomic status and higher body composition and strength traits, as well as lower type 2 diabetes risk and lower glycemic and insulin traits, lipids and lipoproteins, metabolites, and systolic blood pressure (SBP), after FDR adjustment for multiple comparisons (Additional file 1: Table S5). These IVW estimates were confirmed by at least one sensitivity analysis. Although the MR-Egger intercept test indicated potential pleiotropy between birthweight and childhood obesity, waist circumference (WC), type 2 diabetes, and triglycerides (Additional file 1: Table S6), the MR-PRESSO method showed consistent results with the IVW after excluding outlying SNPs.

Similar to birthweight, fetal-specific birthweight showed causal associations with socioeconomic indicators, body composition and strength, glucose metabolism, and lipids and lipoproteins. Moreover, fetal-specific birthweight showed a positive association with occupational attainment and glycine (Additional file 1: Tables S5, S6). Slightly different from birthweight, maternal-specific birthweight did not show associations with glycemic traits, which were observed in the overall birthweight analysis, but showed strong associations with blood pressure.

### Causal associations of mediators with MI and AF

In MVMR, with adjustment for birthweight, each 1-unit increase in genetically determined education, household income, height, appendicular lean mass, and grip strength were associated with a 6-46% lower MI risk, whereas SBP, type 2 diabetes, fasting glucose, 2-h glucose, glycated hemoglobin A1c (HbA1c), fasting insulin, total cholesterol, low-density lipoprotein cholesterol (LDL-C), triglycerides, apolipoprotein B (ApoB), Omega-6 fatty acids, and linoleic acid were causally associated with a 3-103% higher MI risk (Additional file 1: Table S7).

Genetically determined higher childhood obesity, body mass index (BMI), WC, height, and appendicular lean mass were associated with higher AF risks after adjusting for birthweight, with ORs (95% CIs) ranging from 1.08 (1.02–1.13) for childhood obesity to 1.44 (1.28–1.62) for WC (Additional file 1: Table S7). The robustness of the MV-IVW estimates was highly confirmed by the MVMR Egger sensitivity analyses.

Similar independent causal effects were observed for mediators between fetal-specific birthweight and MI or AF, as well as mediators between maternal-specific birthweight and MI (Additional file 1: Table S7).

### Mediators in the associations of birthweight with MI and AF

**Fig. 3:**
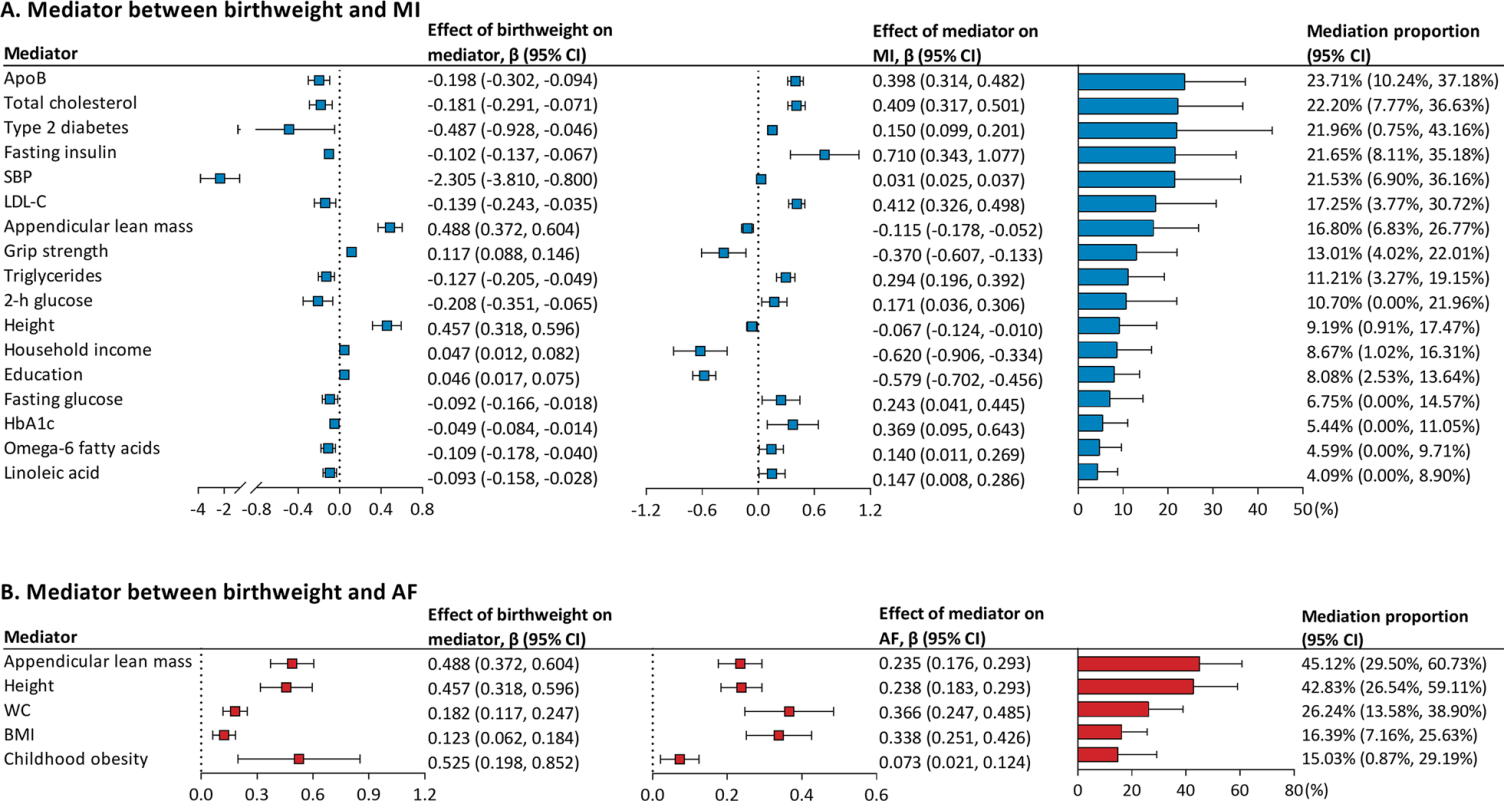
Mediating role of each mediator in the causal associations of birthweight with MI and AF. Two-step MR was used to evaluate the mediating role of each mediator in the causal associations of birthweight with MI (**A**) and AF (**B**). Left: UVMR estimates for the causal effect of birthweight on each mediator. Middle: MVMR estimates for the causal effect of each mediator on MI or AF with adjustment for birthweight. Right: mediation proportion of each mediator in the causal association between birthweight and MI or AF. Negative mediation proportions were truncated at a lower limit of 0%, as it is the lowest threshold to determine a mediation proportion. *AF*  atrial fibrillation, *ApoB*  Apolipoprotein B, *BMI*  body mass index, *CI*  confidence interval, *HbA1c*  Glycated hemoglobin, *LDL-C*  low-density lipoprotein cholesterol, *MI*  myocardial infarction, *MVMR*  multivariable Mendelian randomization, *SBP*  systolic blood pressure, *UVMR*  univariable Mendelian randomization, *WC*  Waist circumference

ApoB (mediation proportion: 23.71%), total cholesterol (22.20%), type 2 diabetes (21.96%), fasting insulin (21.65%), SBP (21.53%), LDL-C (17.25%), appendicular lean mass (16.80%), grip strength (13.01%), triglycerides (11.21%), and 2-h glucose (10.7%) each mediated more than 10% of the total effect of birthweight on MI (Fig. 3A). The other seven mediators including height, household income, education, fasting glucose, HbA1c, Omega-6 fatty acids, and linoleic acid each mediated 9.19–4.09% of the total effect of birthweight on MI. For the causal association between birthweight and AF, mediators ranked by individual mediation proportion included appendicular lean mass (45.12%), height (42.83%), WC (26.24%), BMI (16.39%), and childhood obesity (15.03%; Fig. 3B).

Sensitivity analyses of fetal-specific birthweight further supported the mediating roles for most of the above mediators and suggested two additional mediators (i.e., occupational attainment and glycine) between fetal-specific birthweight and MI (Additional file 1: Table S8). Furthermore, blood pressure primarily mediated the total effect of maternal-specific birthweight on MI (mediation proportion: 43.87% for hypertension, 38.39% for SBP, and 36.94% for diastolic blood pressure).

## Discussion

This MR study demonstrated the opposite causal effects of birthweight on MI and AF and shed light on their distinct mediating pathways. Genetically determined each 1-SD lower birthweight was causally associated with a 40% higher risk of MI, whereas each 1-SD higher birthweight was causally associated with a 29% higher risk of AF. Both fetal-specific birthweight and maternal-specific birthweight showed inverse associations with MI; only fetal-specific birthweight, but not maternal-specific birthweight, showed a positive association with AF. The causal association between lower birthweight and MI risk was substantially mediated by cardiometabolic traits, body composition and strength traits, and socioeconomic indicators. On the other hand, only body composition and strength traits played a mediating role in the association between higher birthweight and AF. Of 52 potential mediators of interest, psychological well-being and lifestyle factors conferred no mediating effect in either association.

High birthweight is mainly caused by maternal hyperglycemia, obesity, and intrauterine overnutrition, while low birthweight strongly represents fetal poor growth and fetal programming due to intrauterine malnutrition [19]. Our study extended previous cohort and MR studies [15–17, 32] by employing fetal- and maternal-specific as well as total effects on birthweight and untangling the developmental origins of the relationships of birthweight with MI and AF [14]. In this study, the causal associations of overall birthweight and fetal- and maternal-specific birthweight with MI indicated that lower birthweight was not a maker but a cause in the pathogeneses of MI, and supported that the restricted intrauterine environment could increase the risk of MI in later life. We also demonstrated that fetal-specific birthweight, rather than maternal-specific birthweight, was causally associated with AF, suggesting that birthweight determined by fetal genetic factors contributes to future AF risk. These findings underscore the long-term health consequences of intrauterine environment and fetal growth, emphasizing the importance of following the critical periods of fetal development closely to improve cardiovascular health later in life.

In view of birthweight being determined early in life and its opposite causal effects on MI and AF, we specifically explored if there are causal mediators in the subsequent life trajectories mediating the relationships of birthweight with MI or AF. Notably, we found that cardiometabolic factors accounted for the majority of mediating roles in the causal pathway from low birthweight to high MI risk. Our findings can be interpreted by the thrifty phenotype hypothesis and evidence from experimental studies that adaptation to a nutritionally depleted intrauterine environment might result in alternations in insulin-signaling pathways, epigenetic modifications, cellular proliferation, and key organ differentiation, which induce disruptions in the metabolism of glucose, lipids, and metabolites and contribute to adult cardiometabolic diseases [13, 33–35]. Intriguingly, blood pressure primarily mediated the association between maternal-specific birthweight and MI. This could be partially explained by basic research indicating that the fetus may respond to an aberrant intrauterine environment through vascular adaptations, including endothelial dysfunction, potentially leading to lifelong alterations in blood pressure and ultimately contributing to MI [13]. We also revealed that socioeconomic indicators including education, household income, and occupational attainment, played a mediating role in the causal association between birthweight and MI, supported by previous studies [36–38]. Taken together, our study highlights the importance of improving socioeconomic status and promoting cardiometabolic health in order to reduce the burden of MI attributable to low birthweight. By contrast, body composition and strength traits including height, appendicular lean mass, and adiposity traits, played a pronounced mediating role in the pathway from higher birthweight to AF. It seems plausible because obesity is a recognized determinant of AF and higher birthweight is associated with obesity in both childhood and adulthood [39, 40].

The strengths of this study included the rigorously designed MR analytical framework to detect the causality of birthweight with MI and AF and the mediation pathways, the employment of comprehensive complementary sensitivity analyses to validate the robustness of the MR results, and the use of summary statistics from large-scale GWASs, enhancing statistical power and precision of causal estimates. This study also had several limitations. First, based on the two-sample MR design, we assumed that the relationships between birthweight and outcomes are linear in UVMR and MVMR analyses. Future studies using individual-level data are warranted to investigate the potential non-linear causal relationships. Second, although we have included common and important candidate mediators to facilitate clinical practice, this study cannot fully capture all mediation pathways, especially non-heritable factors. Third, the potential exposure-mediator interaction cannot be modeled in the present two-sample MR setting. Nonetheless, the MR approach could largely alleviate the potential bias caused by the exposure-mediator interaction [41]. Fourth, we identified potential pleiotropy between the genetic variants of birthweight and childhood obesity, WC, type 2 diabetes, and triglycerides through the MR-Egger intercept test. However, the MR-PRESSO analysis further confirmed that the causal associations remained consistent after excluding the outlying SNPs. Fifth, to ensure consistency in genetic background, this MR study was almost exclusively restricted to European-ancestry individuals; thus, the generalization of our findings to other ethnic groups should be cautious.

In conclusion, this study elucidated that lower birthweight was causally associated with a greater risk of MI, while higher birthweight was associated with a greater risk of AF. Cardiometabolic factors and body composition and strength traits were the primary mediators in the pathway from birthweight to MI and AF, respectively. Our findings provide novel evidence for the pathogenesis of MI and AF and advocate adopting a life-course approach to improving fetal development and targeting subsequent causal mediators to mitigate the prevalence and burden of cardiovascular diseases.

### Supplementary Information

**Additional file 1. **Additional tables and figures.

## Data Availability

All the summary-level GWAS data used in the analyses are publicly available as shown in Table 1. The GWAS data can be obtained through the EGG Consortium (http://egg-consortium.org/), the DIAGRAM consortium (https://diagram-consortium.org/), the GIANT consortium (https://portals.broadinstitute.org/collaboration/giant/index.php/GIANT_consortium_data_files), the SSGAC data portal (http://www.thessgac.org/data), the IEU OpenGWAS project (https://gwas.mrcieu.ac.uk/), and the NHGRI-EBI GWAS Catalog (https://www.ebi.ac.uk/gwas/downloads/summary-statistics). The analytical script of the MR analyses conducted in this study is available via the GitHub repository of the “TwoSampleMR” R package (https://github.com/MRCIEU/TwoSampleMR/).

## Abbreviations

AF: Atrial fibrillation
ApoB: Apolipoprotein B
BMI: Body mass index
CI: Confidence interval
DBP: Diastolic blood pressure
EGG: Early Growth Genetics
FDR: False discovery rate
GWAS: Genome-wide association study
HbA1c: Glycated hemoglobin
IV: Instrumental variable
IVW: Inverse variance weighted
LDL-C: Low-density lipoprotein cholesterol
MI: Myocardial infarction
MR: Mendelian randomization
MR-PRESSO: Mendelian randomization pleiotropy residual sum and outlier
MVMR: Multivariable Mendelian randomization
OR: Odds ratio
SBP: Systolic blood pressure
SD: Standard deviation
SNP: Single nucleotide polymorphism
UVMR: Univariable Mendelian randomization
WC: Waist circumference

## References

[1] Roth GA, Mensah GA, Johnson CO, Addolorato G, Ammirati E, Baddour LM (2020). Global burden of cardiovascular diseases and risk factors, 1990–2019: update from the GBD 2019 study. J Am Coll Cardiol.

[2] Frederiksen TC, Dahm CC, Preis SR, Lin H, Trinquart L, Benjamin EJ (2023). The bidirectional association between atrial fibrillation and myocardial infarction. Nat Rev Cardiol.

[3] Jabre P, Roger VL, Murad MH, Chamberlain AM, Prokop L, Adnet F (2011). Mortality associated with atrial fibrillation in patients with myocardial infarction: a systematic review and meta-analysis. Circulation.

[4] Popovic B, Agrinier N, Bouchahda N, Pinelli S, Maigrat CH, Metzdorf PA (2018). Coronary embolism among ST-segment-elevation myocardial infarction patients: mechanisms and management. Circ Cardiovasc Interv.

[5] Nishida K, Qi XY, Wakili R, Comtois P, Chartier D, Harada M (2011). Mechanisms of atrial tachyarrhythmias associated with coronary artery occlusion in a chronic canine model. Circulation.

[6] Sardu C, Trotta MC, Sasso FC, Sacra C, Carpinella G, Mauro C (2023). SGLT2-inhibitors effects on the coronary fibrous cap thickness and MACEs in diabetic patients with inducible myocardial ischemia and multi vessels non-obstructive coronary artery stenosis. Cardiovasc Diabetol.

[7] Marfella R, Prattichizzo F, Sardu C, Paolisso P, D’Onofrio N, Scisciola L (2023). Evidence of an anti-inflammatory effect of PCSK9 inhibitors within the human atherosclerotic plaque. Atherosclerosis.

[8] Sardu C, Santulli G, Santamaria M, Barbieri M, Sacra C, Paolisso P (2017). Effects of alpha lipoic acid on multiple cytokines and biomarkers and recurrence of atrial fibrillation within 1 year of catheter ablation. Am J Cardiol.

[9] Sardu C, Santamaria M, Paolisso G, Marfella R (2015). microRNA expression changes after atrial fibrillation catheter ablation. Pharmacogenomics.

[10] Gluckman PD, Hanson MA, Cooper C, Thornburg KL (2008). Effect of in utero and early-life conditions on adult health and disease. N Engl J Med.

[11] Singhal A, Lucas A (2004). Early origins of cardiovascular disease: is there a unifying hypothesis?. Lancet.

[12] Hoffman DJ, Powell TL, Barrett ES, Hardy DB (2021). Developmental origins of metabolic diseases. Physiol Rev.

[13] Fleming TP, Watkins AJ, Velazquez MA, Mathers JC, Prentice AM, Stephenson J (2018). Origins of lifetime health around the time of conception: causes and consequences. Lancet.

[14] Warrington NM, Beaumont RN, Horikoshi M, Day FR, Helgeland Ø, Laurin C (2019). Maternal and fetal genetic effects on birth weight and their relevance to cardio-metabolic risk factors. Nat Genet.

[15] Kember RL, Levin MG, Cousminer DL, Tsao N, Judy R, Schur GM (2020). Genetically determined birthweight associates with atrial fibrillation: a Mendelian randomization study. Circ Genom Precis Med.

[16] Zanetti D, Tikkanen E, Gustafsson S, Priest JR, Burgess S, Ingelsson E, Birthweight (2018). Type 2 diabetes mellitus, and cardiovascular disease: addressing the barker hypothesis with Mendelian randomization. Circ Genom Precis Med.

[17] Conen D, Tedrow UB, Cook NR, Buring JE, Albert CM (2010). Birth weight is a significant risk factor for incident atrial fibrillation. Circulation.

[18] Juliusdottir T, Steinthorsdottir V, Stefansdottir L, Sveinbjornsson G, Ivarsdottir EV, Thorolfsdottir RB (2021). Distinction between the effects of parental and fetal genomes on fetal growth. Nat Genet.

[19] Kong L, Ye C, Wang Y, Zheng J, Zhao Z, Li M (2023). Causal effect of lower birthweight on non-alcoholic fatty liver disease and mediating roles of insulin resistance and metabolites. Liver Int.

[20] Davies NM, Holmes MV, Davey Smith G (2018). Reading Mendelian randomisation studies: a guide, glossary, and checklist for clinicians. BMJ.

[21] Relton CL, Davey Smith G (2012). Two-step epigenetic Mendelian randomization: a strategy for establishing the causal role of epigenetic processes in pathways to disease. Int J Epidemiol.

[22] Skrivankova VW, Richmond RC, Woolf BAR, Davies NM, Swanson SA, VanderWeele TJ (2021). Strengthening the reporting of observational studies in epidemiology using Mendelian randomisation (STROBE-MR): explanation and elaboration. BMJ.

[23] Kong L, Ye C, Wang Y, Hou T, Zheng J, Zhao Z (2023). Genetic evidence for causal effects of socioeconomic, lifestyle, and cardiometabolic factors on epigenetic-age acceleration. J Gerontol A Biol Sci Med Sci.

[24] Hartiala JA, Han Y, Jia Q, Hilser JR, Huang P, Gukasyan J (2021). Genome-wide analysis identifies novel susceptibility loci for myocardial infarction. Eur Heart J.

[25] Nielsen JB, Thorolfsdottir RB, Fritsche LG, Zhou W, Skov MW, Graham SE (2018). Biobank-driven genomic discovery yields new insight into atrial fibrillation biology. Nat Genet.

[26] Lawlor DA, Harbord RM, Sterne JA, Timpson N, Davey Smith G (2008). Mendelian randomization: using genes as instruments for making causal inferences in epidemiology. Stat Med.

[27] Carter AR, Sanderson E, Hammerton G, Richmond RC, Davey Smith G, Heron J (2021). Mendelian randomisation for mediation analysis: current methods and challenges for implementation. Eur J Epidemiol.

[28] Bowden J, Davey Smith G, Haycock PC, Burgess S (2016). Consistent estimation in Mendelian randomization with some invalid instruments using a weighted median estimator. Genet Epidemiol.

[29] Hartwig FP, Davey Smith G, Bowden J (2017). Robust inference in summary data Mendelian randomization via the zero modal pleiotropy assumption. Int J Epidemiol.

[30] Burgess S, Bowden J, Fall T, Ingelsson E, Thompson SG (2017). Sensitivity analyses for robust causal inference from Mendelian randomization analyses with multiple genetic variants. Epidemiology.

[31] Verbanck M, Chen CY, Neale B, Do R (2018). Detection of widespread horizontal pleiotropy in causal relationships inferred from Mendelian randomization between complex traits and diseases. Nat Genet.

[32] Wang SF, Shu L, Sheng J, Mu M, Wang S, Tao XY (2014). Birth weight and risk of coronary heart disease in adults: a meta-analysis of prospective cohort studies. J Dev Orig Health Dis.

[33] Hughes AE, Hattersley AT, Flanagan SE, Freathy RM (2021). Two decades since the fetal insulin hypothesis: what have we learned from genetics?. Diabetologia.

[34] Lelièvre-Pégorier M, Vilar J, Ferrier ML, Moreau E, Freund N, Gilbert T (1998). Mild vitamin A deficiency leads to inborn nephron deficit in the rat. Kidney Int.

[35] Reynolds RM (2013). Glucocorticoid excess and the developmental origins of disease: two decades of testing the hypothesis–2012 Curt Richter award winner. Psychoneuroendocrinology.

[36] Currie J, Hyson R (1999). Is the impact of health shocks cushioned by socioeconomic status? The case of low birthweight. Am Econ Rev.

[37] Ye CJ, Kong LJ, Wang YY, Dou C, Zheng J, Xu M (2023). Mendelian randomization evidence for the causal effects of socio-economic inequality on human longevity among Europeans. Nat Hum Behav.

[38] Wang Y, Ye C, Kong L, Zheng J, Xu M, Xu Y (2023). Independent associations of education, intelligence, and cognition with hypertension and the mediating effects of cardiometabolic risk factors: a Mendelian randomization study. Hypertension.

[39] Schellong K, Schulz S, Harder T, Plagemann A (2012). Birth weight and long-term overweight risk: systematic review and a meta-analysis including 643,902 persons from 66 studies and 26 countries globally. PLoS ONE.

[40] Powell-Wiley TM, Poirier P, Burke LE, Després JP, Gordon-Larsen P, Lavie CJ (2021). Obesity and cardiovascular disease: a scientific statement from the American Heart Association. Circulation.

[41] Emdin CA, Khera AV, Kathiresan S (2017). Mendelian randomization. JAMA.

