# Supplementary material for: Opposite causal effects of birthweight on myocardial infarction and atrial fibrillation and the distinct mediating pathways: a Mendelian randomization study
Source: Cardiovasc Diabetol. 2023 Dec 12;22:338. doi: 10.1186/s12933-023-02062-5 (PMC10716951; doi:10.1186/s12933-023-02062-5)

Supplementary material

Table of contents

**Table S1.** STROBE-MR checklist of the study	…….….…….2
**Table S2.** 205 autosomal SNPs for birthweight, 63 SNPs with fetal-only effects for fetal-specific birthweight, and 31 SNPs with maternal-only effects for maternal-specific birthweight	……….….….5
**Table S3.** Evidence for the relationships between the 52 candidate mediators and MI or AF from previous literature	…………..….18
**Table S4.** UVMR pleiotropy and heterogeneity test for the causal associations of birthweight with MI and AF	………..…….22
**Table S5.** UVMR estimates for the causal associations between birthweight and candidate mediators	………..…….23
**Table S6.** UVMR pleiotropy and heterogeneity test for the causal associations between birthweight and candidate mediators	………..…….63
**Table S7.** MVMR estimates for the causal associations of candidate mediators with MI and AF with adjustment for birthweight	………..…….70
**Table S8.** Mediation analysis of the effect of fetal/maternal-specific birthweight on MI and AF via mediators	………..…….81
**Fig. S1.** Leave-one-out analysis for the associations of birthweight with MI and AF	………..…….83
**Fig. S2.** Leave-one-out analysis for the associations of fetal/maternal-specific birthweight with MI and AF	………..…….84

Table S1. STROBE-MR checklist of the study[1]

Section	Item No.	Checklist item	Page No.
**Title and abstract**	1	Indicate Mendelian randomization (MR) as the study's design in the title and/or the abstract if that is a main purpose of the study	1,3
**Introduction**			
Background	2	Explain the scientific background and rationale for the reported study. What is the exposure? Is a potential causal relationship between exposure and outcome plausible? Justify why MR is a helpful method to address the study question	5
Objectives	3	State specific objectives clearly, including pre-specified causal hypotheses (if any). State that MR is a method that, under specific assumptions, intends to estimate causal effects	6
**Methods**			
Study design and data sources	4	Present key elements of the study design early in the article. Consider including a table listing sources of data for all phases of the study. For each data source contributing to the analysis, describe the following:	
		a) Setting: Describe the study design and the underlying population, if possible. Describe the setting, locations, and relevant dates, including periods of recruitment, exposure, follow-up, and data collection, when available.	6, Figure 1
		b) Participants: Give the eligibility criteria, and the sources and methods of selection of participants. Report the sample size, and whether any power or sample size calculations were carried out prior to the main analysis	7-9
		c) Describe measurement, quality control and selection of genetic variants	7-9
		d) For each exposure, outcome, and other relevant variables, describe methods of assessment and diagnostic criteria for diseases	7-9
		e) Provide details of ethics committee approval and participant informed consent, if relevant	7
Assumptions	5	Explicitly state the three core IV assumptions for the main analysis (relevance, independence and exclusion restriction) as well assumptions for any additional or sensitivity analysis	7
Statistical methods: main analysis	6	Describe statistical methods and statistics used	
		a) Describe how quantitative variables were handled in the analyses (i.e., scale, units, model)	7-9, Table 1
		b) Describe how genetic variants were handled in the analyses and, if applicable, how their weights were selected	7-9
		c) Describe the MR estimator (e.g. two-stage least squares, Wald ratio) and related statistics. Detail the included covariates and, in case of two-sample MR, whether the same covariate set was used for adjustment in the two samples	10-12
		d) Explain how missing data were addressed	N/A
		e) If applicable, indicate how multiple testing was addressed	12
Assessment of assumptions	7	Describe any methods or prior knowledge used to assess the assumptions or justify their validity	10-12

Sensitivity analyses and additional analyses	8	Describe any sensitivity analyses or additional analyses performed (e.g. comparison of effect estimates from different approaches, independent replication, bias analytic techniques, validation of instruments, simulations)	10-12
Software and pre-registration	9	a) Name statistical software and package(s), including version and settings used	12
		b) State whether the study protocol and details were pre-registered (as well as when and where)	7
**Results**			
Descriptive data	10	a) Report the numbers of individuals at each stage of included studies and reasons for exclusion. Consider use of a flow diagram	Table 1
		b) Report summary statistics for phenotypic exposure(s), outcome(s), and other relevant variables (e.g. means, SDs, proportions)	7-9, Table 1
		c) If the data sources include meta-analyses of previous studies, provide the assessments of heterogeneity across these studies	9
		d) For two-sample MR:<br>i. Provide justification of the similarity of the genetic variant-exposure associations between the exposure and outcome samples<br>ii. Provide information on the number of individuals who overlap between the exposure and outcome studies	7-9
Main results	11	a) Report the associations between genetic variant and exposure, and between genetic variant and outcome, preferably on an interpretable scale	12-15
		b) Report MR estimates of the relationship between exposure and outcome, and the measures of uncertainty from the MR analysis, on an interpretable scale, such as odds ratio or relative risk per SD difference	12-15
		c) If relevant, consider translating estimates of relative risk into absolute risk for a meaningful time period	NA
		d) Consider plots to visualize results (e.g. forest plot, scatterplot of associations between genetic variants and outcome versus between genetic variants and exposure)	Figure 3
Assessment of assumptions	12	a) Report the assessment of the validity of the assumptions	12-15
		b) Report any additional statistics (e.g., assessments of heterogeneity across genetic variants, such as I2, Q statistic or E-value)	12-15, Table S4, S6
Sensitivity analyses and additional analyses	13	a) Report any sensitivity analyses to assess the robustness of the main results to violations of the assumptions	12-15, Table S5, S7
		b) Report results from other sensitivity analyses or additional analyses	12-15, Table S5, S7
		c) Report any assessment of direction of causal relationship (e.g., bidirectional MR)	N/A
		d) When relevant, report and compare with estimates from non-MR analyses	16-17
		e) Consider additional plots to visualize results (e.g., leave-one-out analyses)	Figure S1, S2
**Discussion**			
Key results	14	Summarize key results with reference to study objectives	15-16
Limitations	15	Discuss limitations of the study, taking into account the validity of the IV assumptions, other sources of potential bias, and imprecision. Discuss both direction and	18

		magnitude of any potential bias and any efforts to address them	
Interpretation	16	a) Meaning: Give a cautious overall interpretation of results in the context of their limitations in comparison with other studies	18
		b) Mechanism: Discuss underlying biological mechanisms that could drive a potential causal relationship between the investigated exposure and the outcome, and whether the gene-environment equivalence assumption is reasonable. Use causal language carefully, clarifying that IV estimates may provide causal effects only under certain assumptions	16-17
		c) Clinical relevance: Discuss whether the results have clinical or public policy relevance, and to what extent they inform effect sizes of possible interventions	16-17
Generalizability	17	Discuss the generalizability of the study results (a) to other populations, (b) across other exposure periods/timings, and (c) across other levels of exposure	18
**Other information**			
Funding	18	Describe sources of funding and the role of funders in the present study and, if applicable, sources of funding for the databases and original study or studies on which the present study is based	21
Data and data sharing	19	Provide the data used to perform all analyses or report where and how the data can be accessed, and reference these sources in the article. Provide the statistical code needed to reproduce the results in the article, or report whether the code is publicly accessible and if so, where	21
Conflicts of Interest	20	All authors should declare all potential conflicts of interest	21

Reference

1. Skrivankova VW, Richmond RC, Woolf BAR, et al. Strengthening the Reporting of Observational Studies in Epidemiology using Mendelian Randomisation (STROBE-MR): Explanation and Elaboration. BMJ. 2021;375:n2233.

Table S2. 205 autosomal SNPs for birthweight, 63 SNPs with fetal-only effects for fetal-specific birthweight, and 31 SNPs with maternal-only effects for maternal-specific birthweight

Phenotype	SNP	Chr	Pos	effect_allele	other_allele	eaf	beta	se	pval
birthweight	rs17367504	1	11862778	G	A	0.161	0.012	0.003	8.30E-04
birthweight	rs12401656	1	43456767	G	A	0.865	0.025	0.004	3.40E-11
birthweight	rs80278614	1	119412317	A	G	0.054	0.04	0.006	6.50E-12
birthweight	rs905938	1	154991389	C	T	0.262	0.026	0.003	2.80E-19
birthweight	rs670523	1	155878732	G	A	0.669	0.019	0.003	7.60E-12
birthweight	rs72480273	1	161644871	C	A	0.182	0.023	0.003	4.00E-11
birthweight	rs10913200	1	176521655	G	A	0.972	0.051	0.008	2.00E-10
birthweight	rs61830764	1	212289976	A	G	0.377	0.017	0.003	1.10E-09
birthweight	rs3806315	1	214724668	A	G	0.591	0.018	0.003	2.80E-11
birthweight	rs708122	1	228216997	C	A	0.681	0.017	0.003	2.50E-09
birthweight	rs10495563	2	9662210	A	G	0.664	0.022	0.003	2.10E-16
birthweight	rs11893688	2	9695282	T	C	0.661	0.022	0.003	1.30E-15
birthweight	rs2551347	2	23912401	T	C	0.749	0.024	0.003	1.90E-16
birthweight	rs1179494	2	36809496	G	C	0.676	0.01	0.003	1.50E-04
birthweight	rs754868	2	43185532	G	A	0.419	0.016	0.003	6.70E-10
birthweight	rs4952673	2	43423870	A	G	0.474	0.007	0.003	3.80E-03
birthweight	rs17034876	2	46484310	T	C	0.7	0.042	0.003	3.10E-47
birthweight	rs4953353	2	46567276	G	T	0.632	0.018	0.003	3.50E-11
birthweight	rs186606513	2	97482001	G	A	0.978	0.061	0.01	2.70E-09
birthweight	rs56188432	2	158406865	G	A	0.002	0.258	0.031	1.40E-16
birthweight	rs560887	2	169763148	C	T	0.7	-0.008	0.003	5.80E-03

birthweight	rs2280235	2	191843830	G	A	0.259	0.018	0.003	6.90E-10
birthweight	rs10181515	2	227019461	T	C	0.225	0.021	0.003	2.10E-12
birthweight	rs9855896	3	14287150	G	A	0.214	0.004	0.003	0.186
birthweight	rs2168443	3	46947087	T	A	0.379	0.017	0.003	3.90E-10
birthweight	rs11708067	3	123065778	G	A	0.238	0.041	0.003	1.60E-42
birthweight	rs9851257	3	123125711	T	A	0.733	0.02	0.003	2.40E-12
birthweight	rs6440006	3	141142691	A	G	0.446	0.01	0.003	7.30E-05
birthweight	rs2306700	3	142123841	T	C	0.136	0.023	0.004	1.80E-09
birthweight	rs10935733	3	148622968	T	C	0.399	0.019	0.003	2.30E-13
birthweight	rs4679760	3	155855418	G	C	0.581	0.009	0.003	3.20E-04
birthweight	rs1482852	3	156798294	A	G	0.599	0.05	0.003	1.60E-82
birthweight	rs11711420	3	183349010	T	G	0.747	0.019	0.003	3.20E-10
birthweight	rs4144829	4	17903654	C	T	0.267	0.036	0.003	4.30E-34
birthweight	rs2174633	4	17917781	A	C	0.27	0.035	0.003	7.10E-33
birthweight	rs2189234	4	106075498	G	T	0.618	0.015	0.003	1.20E-08
birthweight	rs6533183	4	106133184	C	T	0.352	0.022	0.003	6.80E-16
birthweight	rs116807401	4	135121721	C	T	0.018	0.077	0.01	2.20E-13
birthweight	rs6845999	4	145565826	T	C	0.431	0.026	0.003	1.50E-24
birthweight	rs2131354	4	145599908	A	G	0.527	0.026	0.003	3.50E-24
birthweight	rs4579095	4	174726635	G	A	0.402	0.003	0.003	0.319
birthweight	rs1818782	5	39424628	C	A	0.637	0.016	0.003	4.20E-09
birthweight	rs351930	5	52003397	T	A	0.801	0.019	0.003	2.90E-09
birthweight	rs854037	5	57091783	A	G	0.814	0.027	0.003	9.40E-16
birthweight	rs28365970	5	67585723	C	A	0.741	0.02	0.003	1.70E-11

birthweight	rs6871635	5	133830395	G	A	0.566	0.016	0.003	3.00E-09
birthweight	rs1981627	5	133838180	G	A	0.585	0.017	0.003	8.40E-11
birthweight	rs2946179	5	157886627	C	T	0.734	0.02	0.003	1.10E-11
birthweight	rs34471628	5	172196752	A	G	0.962	0.018	0.007	9.40E-03
birthweight	rs9379084	6	7231843	G	A	0.883	0.022	0.004	1.20E-07
birthweight	rs35261542	6	20675792	C	A	0.733	0.041	0.003	2.80E-45
birthweight	rs9379832	6	26186200	A	G	0.73	0.022	0.003	1.10E-13
birthweight	rs9366778	6	31269173	G	A	0.627	0.018	0.003	2.90E-11
birthweight	rs6911024	6	31368451	T	C	0.901	0.017	0.004	1.30E-04
birthweight	rs9267812	6	32128394	T	C	0.133	0.023	0.004	3.10E-09
birthweight	rs1547669	6	33775641	G	A	0.497	0.018	0.003	6.20E-12
birthweight	rs75104038	6	34190104	A	G	0.06	0.045	0.006	4.30E-16
birthweight	rs75034466	6	34199815	T	C	0.046	0.046	0.006	1.80E-13
birthweight	rs6911621	6	35529025	T	C	0.344	0.018	0.003	1.60E-11
birthweight	rs9348981	6	35687249	T	G	0.71	0.021	0.003	2.20E-13
birthweight	rs7744700	6	53349401	T	A	0.711	0.02	0.003	1.60E-11
birthweight	rs76094073	6	109288036	G	C	0.121	0.027	0.004	1.60E-11
birthweight	rs6568554	6	109290319	A	C	0.145	0.021	0.004	1.10E-08
birthweight	rs6925689	6	126865884	T	C	0.494	0.015	0.003	6.40E-09
birthweight	rs6569647	6	130337266	T	C	0.802	0.02	0.003	6.30E-10
birthweight	rs1415701	6	130345835	G	A	0.736	0.018	0.003	1.40E-09
birthweight	rs6930558	6	141878920	T	G	0.747	0.022	0.003	3.40E-13
birthweight	rs962554	6	142734204	T	C	0.715	0.017	0.003	3.80E-09
birthweight	rs10872678	6	152039964	T	C	0.724	0.032	0.003	9.80E-29

birthweight	rs7772579	6	152042502	A	C	0.721	0.031	0.003	6.40E-28
birthweight	rs2934844	6	166142456	T	A	0.672	0.021	0.003	1.80E-13
birthweight	rs1724889	7	2741021	G	A	0.735	0.016	0.003	1.50E-07
birthweight	rs4719648	7	2756832	C	T	0.577	0.019	0.003	2.60E-13
birthweight	rs59084784	7	22739562	A	C	0.323	0.017	0.003	2.40E-09
birthweight	rs7808457	7	22798265	A	T	0.586	0.01	0.003	7.10E-05
birthweight	rs34776209	7	23513093	C	T	0.755	0.023	0.003	8.50E-15
birthweight	rs2908279	7	44174857	T	G	0.495	0.011	0.003	2.20E-05
birthweight	rs2971669	7	44231778	T	C	0.214	0.011	0.003	4.20E-04
birthweight	rs138715366	7	44246271	C	T	0.991	0.24	0.015	4.00E-61
birthweight	rs10265133	7	45895604	G	T	0.859	0.001	0.004	0.859
birthweight	rs11983722	7	46298647	A	T	0.938	0.032	0.005	3.10E-09
birthweight	rs10265057	7	47275737	G	A	0.092	0.027	0.004	1.30E-09
birthweight	rs2237467	7	50733316	A	G	0.221	0.018	0.003	5.30E-09
birthweight	rs112139215	7	73034559	A	C	0.068	0.047	0.005	2.80E-20
birthweight	rs2282978	7	92264410	C	T	0.326	0.018	0.003	1.70E-11
birthweight	rs45446698	7	99332948	G	T	0.041	0.025	0.007	1.70E-04
birthweight	rs13231367	7	127509070	G	A	0.714	0.017	0.003	4.40E-09
birthweight	rs6467157	7	127660763	T	C	0.713	0.02	0.003	1.50E-11
birthweight	rs3918226	7	150690176	C	T	0.919	0.015	0.005	1.90E-03
birthweight	rs62496903	8	6446938	T	C	0.083	0.033	0.005	6.70E-12
birthweight	rs732563	8	23345526	C	T	0.504	0.017	0.003	1.30E-11
birthweight	rs11778247	8	23403378	G	A	0.835	0.014	0.003	8.20E-05
birthweight	rs34036147	8	38366249	T	C	0.688	0.018	0.003	8.40E-11

birthweight	rs13266210	8	41533514	A	G	0.786	0.027	0.003	1.50E-17
birthweight	rs72656010	8	57122215	T	C	0.868	0.028	0.004	1.40E-13
birthweight	rs6995390	8	77611012	T	A	0.163	0.006	0.003	0.079
birthweight	rs7819593	8	106115172	C	T	0.243	0.022	0.003	6.20E-13
birthweight	rs10283100	8	120596023	G	A	0.946	0.042	0.006	7.00E-13
birthweight	rs13271368	8	126506140	C	T	0.761	0.02	0.003	2.30E-11
birthweight	rs13257363	8	142252580	G	A	0.591	0.018	0.003	2.00E-11
birthweight	rs9657468	8	142362391	G	T	0.334	0.015	0.003	7.90E-08
birthweight	rs7854962	9	96900505	C	G	0.785	0.022	0.003	1.00E-11
birthweight	rs28457693	9	98217348	G	A	0.109	0.044	0.004	9.90E-26
birthweight	rs1411424	9	113892963	A	G	0.523	0.02	0.003	1.50E-14
birthweight	rs2418135	9	113901309	A	G	0.522	0.02	0.003	1.50E-14
birthweight	rs72760655	9	116916214	C	A	0.681	0.007	0.003	0.01
birthweight	rs1323438	9	119115531	C	T	0.718	0.019	0.003	5.60E-11
birthweight	rs3933326	9	123633948	G	A	0.676	0.021	0.003	2.30E-14
birthweight	rs10985827	9	125701608	G	T	0.141	0.03	0.004	6.10E-16
birthweight	rs28505901	9	139241030	A	G	0.249	0.024	0.003	2.50E-15
birthweight	rs4350272	10	25056118	A	G	0.269	0.017	0.003	3.60E-09
birthweight	rs5030938	10	70975916	T	C	0.686	0.024	0.003	1.20E-17
birthweight	rs9645500	10	70986723	G	T	0.694	0.024	0.003	1.80E-18
birthweight	rs1112718	10	94479107	G	A	0.404	0.026	0.003	3.80E-23
birthweight	rs10509669	10	95969913	A	T	0.746	-0.001	0.003	0.685
birthweight	rs3740360	10	96025491	C	A	0.109	0.026	0.004	4.00E-10
birthweight	rs2274224	10	96039597	C	G	0.434	0.021	0.003	9.80E-17

birthweight	rs562974282	10	104201070	G	T	0.002	0.021	0.034	0.532
birthweight	rs10883846	10	104958244	C	T	0.615	0.017	0.003	1.30E-10
birthweight	rs7903146	10	114758349	T	C	0.285	0.011	0.003	5.30E-05
birthweight	rs7076938	10	115789375	T	C	0.735	0.032	0.003	2.10E-28
birthweight	rs1801253	10	115805056	C	G	0.727	0.031	0.003	1.40E-25
birthweight	rs71486610	10	124134803	C	G	0.477	0.02	0.003	3.20E-15
birthweight	rs11042596	11	2118860	T	G	0.336	0.027	0.003	4.30E-22
birthweight	rs234864	11	2857297	A	G	0.547	0.016	0.003	1.70E-09
birthweight	rs2168101	11	8255408	C	A	0.689	0.007	0.003	0.011
birthweight	rs4444073	11	10331664	A	C	0.52	0.02	0.003	2.70E-15
birthweight	rs12574749	11	32405355	C	A	0.723	0.015	0.003	1.10E-07
birthweight	rs5030317	11	32410337	C	G	0.733	0.017	0.003	2.70E-09
birthweight	rs10437653	11	46297631	A	C	0.503	0.01	0.003	1.50E-04
birthweight	rs10734564	11	48160429	A	G	0.181	0.005	0.003	0.106
birthweight	rs667515	11	69449076	G	C	0.618	0.018	0.003	9.30E-12
birthweight	rs61885091	11	69791952	A	G	0.169	0.023	0.004	4.80E-10
birthweight	rs10830963*	11	92708710	G	C	0.277	0.019	0.003	2.80E-11
birthweight	rs10895278	11	102095335	C	T	0.338	0.011	0.003	6.60E-05
birthweight	rs76895963	12	4384844	G	T	0.021	0.076	0.01	2.50E-13
birthweight	rs11055030	12	12878349	G	C	0.718	0.02	0.003	3.90E-12
birthweight	rs2306547	12	26877885	C	T	0.534	0.019	0.003	4.40E-13
birthweight	rs11051061	12	30914668	A	G	0.266	0.011	0.003	1.10E-04
birthweight	rs6582623	12	46613394	C	T	0.869	0.024	0.004	1.10E-09
birthweight	rs180438	12	47187260	G	A	0.192	0.011	0.003	7.50E-04

birthweight	rs8756	12	66359752	C	A	0.487	0.041	0.003	2.40E-59
birthweight	rs7968682	12	66371880	G	T	0.486	0.042	0.003	4.20E-60
birthweight	rs1480470	12	66412130	G	A	0.631	0.024	0.003	1.40E-19
birthweight	rs1533688	12	102772745	C	T	0.769	0.005	0.003	0.09
birthweight	rs2647873	12	103081192	A	G	0.52	0.018	0.003	2.90E-12
birthweight	rs17033114	12	103123339	T	C	0.94	0.016	0.006	7.70E-03
birthweight	rs3184504	12	111884608	C	T	0.521	0.023	0.003	2.60E-19
birthweight	rs9549046	13	40647206	A	G	0.118	0.029	0.004	8.00E-13
birthweight	rs34217484	13	48854550	A	T	0.264	0.019	0.003	6.80E-11
birthweight	rs9318511	13	78601413	C	A	0.873	0.027	0.004	6.00E-12
birthweight	rs72681869	14	50655357	C	G	0.011	0.084	0.013	5.90E-10
birthweight	rs6575803	14	101257755	C	T	0.895	0.032	0.004	1.30E-12
birthweight	rs75844534	15	38667117	A	C	0.124	0.026	0.004	4.90E-11
birthweight	rs2928148	15	41401550	A	G	0.526	0.006	0.003	0.018
birthweight	rs339969	15	60883281	A	C	0.619	0.017	0.003	2.20E-10
birthweight	rs3784789	15	75082552	G	C	0.659	-0.004	0.003	0.152
birthweight	rs12909648	15	86224570	G	A	0.524	0.012	0.003	1.70E-06
birthweight	rs12443252	15	91064690	T	C	0.548	0.006	0.003	0.017
birthweight	rs7183988	15	91428589	G	T	0.529	0.018	0.003	1.70E-12
birthweight	rs4932373	15	91429287	A	C	0.68	0.02	0.003	3.00E-13
birthweight	rs55958435	15	96852638	A	G	0.748	0.025	0.003	1.60E-16
birthweight	rs7402983	15	99193276	A	C	0.405	0.024	0.003	2.60E-19
birthweight	rs11630479	15	99240481	G	A	0.703	0.014	0.003	8.90E-07
birthweight	rs2045457	16	20046115	G	A	0.311	0.016	0.003	6.30E-09

birthweight	rs40434	16	55699525	G	A	0.391	0.017	0.003	3.00E-10
birthweight	rs28544888	16	55741204	C	T	0.911	0.026	0.005	1.60E-08
birthweight	rs11641308	16	75312023	T	C	0.35	0.007	0.003	0.013
birthweight	rs222857	17	7164563	T	C	0.575	0.026	0.003	1.10E-24
birthweight	rs2428362?	17	7180274	T	C	0.576	0.025	0.003	1.80E-22
birthweight	rs4511593	17	7455536	T	C	0.65	0.017	0.003	1.10E-10
birthweight	rs78378222	17	7571752	G	T	0.013	0.079	0.012	1.80E-11
birthweight	rs9909342	17	25652275	A	G	0.381	0.018	0.003	2.20E-11
birthweight	rs7223535	17	29211667	G	A	0.732	0.021	0.003	2.10E-13
birthweight	rs11867479	17	68090207	T	C	0.353	0.017	0.003	1.10E-10
birthweight	rs10221267	17	68464662	T	C	0.512	0.017	0.003	6.50E-11
birthweight	rs73354194	17	79905947	C	T	0.025	0.061	0.009	1.00E-11
birthweight	rs9912553	17	79959703	G	C	0.726	0.014	0.003	1.70E-06
birthweight	rs11082304	18	20720973	T	G	0.508	0.016	0.003	4.20E-10
birthweight	rs2779165	19	4915447	G	C	0.184	0.022	0.003	7.60E-11
birthweight	rs8106042	19	7161849	G	C	0.281	0.02	0.003	2.20E-12
birthweight	rs2967676	19	8789666	A	C	0.845	0.021	0.004	1.10E-08
birthweight	rs41355649	19	33790556	G	A	0.934	0.034	0.005	1.20E-10
birthweight	rs1129156	19	40719076	T	C	0.268	0.017	0.003	2.50E-09
birthweight	rs147957154	19	43431040	T	C	0.132	0.023	0.004	2.80E-09
birthweight	rs516246	19	49206172	C	T	0.506	0.018	0.003	9.30E-12
birthweight	rs255773	19	54723546	C	T	0.536	0.018	0.003	1.30E-11
birthweight	rs147110934	19	55993436	G	T	0.975	0.052	0.009	1.60E-09
birthweight	rs12461110	19	56320663	A	G	0.358	0.006	0.003	0.028

birthweight	rs304001	19	56423668	G	A	0.395	0.009	0.003	8.50E-04
birthweight	rs6040076	20	10658882	C	G	0.5	0.019	0.003	4.40E-13
birthweight	rs6033062	20	11207419	A	T	0.46	0.016	0.003	5.20E-10
birthweight	rs1203876	20	22540915	C	A	0.046	0.038	0.006	9.40E-10
birthweight	rs11698914	20	31327144	C	G	0.233	0.032	0.003	1.20E-24
birthweight	rs181451002	20	32466219	G	A	0.979	0.02	0.009	0.026
birthweight	rs2889874	20	33715777	G	T	0.452	0.016	0.003	9.40E-10
birthweight	rs1012167	20	39159119	C	T	0.401	0.024	0.003	1.20E-19
birthweight	rs753381	20	39797465	T	C	0.451	0.015	0.003	3.40E-09
birthweight	rs6026449	20	57272617	C	T	0.627	0.017	0.003	2.50E-10
birthweight	rs73143584	20	62445702	A	G	0.11	0.029	0.004	1.80E-11
birthweight	rs2229742	21	16339172	G	C	0.881	0.027	0.004	7.40E-11
birthweight	rs220193	21	43581308	A	G	0.225	0.021	0.003	4.10E-11
birthweight	rs134594	22	29468456	C	T	0.351	0.017	0.003	5.80E-10
birthweight	rs41311445	22	42070374	A	C	0.903	0.033	0.004	3.30E-13
birthweight	rs7285579	22	46441980	C	T	0.698	0.017	0.003	2.70E-09
fetal-only birthweight	rs12401656	1	43456767	G	A	0.865	0.029	0.006	1.60E-06
fetal-only birthweight	rs80278614	1	119412317	A	G	0.054	0.052	0.009	4.00E-08
fetal-only birthweight	rs61830764	1	212289976	A	G	0.377	0.018	0.004	3.00E-05
fetal-only birthweight	rs2551347	2	23912401	T	C	0.749	0.029	0.005	2.20E-09
fetal-only birthweight	rs754868	2	43185532	G	A	0.419	0.019	0.004	4.70E-06
fetal-only birthweight	rs4953353	2	46567276	G	T	0.632	0.019	0.004	6.50E-06
fetal-only birthweight	rs56188432	2	158406865	G	A	0.002	0.25	0.049	2.60E-07
fetal-only birthweight	rs10181515	2	227019461	T	C	0.225	0.021	0.005	1.30E-05

fetal-only birthweight	rs10935733	3	148622968	T	C	0.399	0.021	0.004	5.00E-07
fetal-only birthweight	rs1482852	3	156798294	A	G	0.599	0.054	0.004	7.60E-39
fetal-only birthweight	rs11711420	3	183349010	T	G	0.747	0.022	0.005	2.70E-06
fetal-only birthweight	rs4144829	4	17903654	C	T	0.267	0.032	0.005	1.10E-11
fetal-only birthweight	rs116807401	4	135121721	C	T	0.018	0.088	0.016	7.00E-08
fetal-only birthweight	rs351930	5	52003397	T	A	0.801	0.02	0.005	8.10E-05
fetal-only birthweight	rs1547669	6	33775641	G	A	0.497	0.018	0.004	9.30E-06
fetal-only birthweight	rs6925689	6	126865884	T	C	0.494	0.018	0.004	2.10E-05
fetal-only birthweight	rs6930558	6	141878920	T	G	0.747	0.022	0.005	3.30E-06
fetal-only birthweight	rs10872678	6	152039964	T	C	0.724	0.028	0.005	8.20E-10
fetal-only birthweight	rs7772579	6	152042502	A	C	0.721	0.027	0.005	5.80E-09
fetal-only birthweight	rs138715366	7	44246271	C	T	0.991	0.235	0.022	1.40E-25
fetal-only birthweight	rs10265057	7	47275737	G	A	0.092	0.036	0.007	4.60E-07
fetal-only birthweight	rs112139215	7	73034559	A	C	0.068	0.056	0.008	1.20E-11
fetal-only birthweight	rs2282978	7	92264410	C	T	0.326	0.021	0.004	1.60E-06
fetal-only birthweight	rs732563	8	23345526	C	T	0.504	0.019	0.004	6.10E-06
fetal-only birthweight	rs34036147	8	38366249	T	C	0.688	0.019	0.004	1.60E-05
fetal-only birthweight	rs13266210	8	41533514	A	G	0.786	0.03	0.005	3.10E-09
fetal-only birthweight	rs72656010	8	57122215	T	C	0.868	0.026	0.006	1.60E-05
fetal-only birthweight	rs7819593	8	106115172	C	T	0.243	0.023	0.005	2.10E-06
fetal-only birthweight	rs9657468	8	142362391	G	T	0.334	0.018	0.004	3.60E-05
fetal-only birthweight	rs28457693	9	98217348	G	A	0.109	0.04	0.007	1.70E-09
fetal-only birthweight	rs1323438	9	119115531	C	T	0.718	0.02	0.005	1.30E-05
fetal-only birthweight	rs3933326	9	123633948	G	A	0.676	0.023	0.004	2.20E-07

fetal-only birthweight	rs28505901	9	139241030	A	G	0.249	0.024	0.005	4.20E-07
fetal-only birthweight	rs7076938	10	115789375	T	C	0.735	0.029	0.005	2.90E-10
fetal-only birthweight	rs11042596	11	2118860	T	G	0.336	0.027	0.004	1.60E-09
fetal-only birthweight	rs234864	11	2857297	A	G	0.547	0.017	0.004	4.90E-05
fetal-only birthweight	rs4444073	11	10331664	A	C	0.52	0.023	0.004	2.20E-08
fetal-only birthweight	rs11055030	12	12878349	G	C	0.718	0.022	0.005	1.00E-06
fetal-only birthweight	rs8756	12	66359752	C	A	0.487	0.037	0.004	1.70E-19
fetal-only birthweight	rs7968682	12	66371880	G	T	0.486	0.037	0.004	4.90E-20
fetal-only birthweight	rs1480470	12	66412130	G	A	0.631	0.028	0.004	1.10E-10
fetal-only birthweight	rs72681869	14	50655357	C	G	0.011	0.108	0.021	2.70E-07
fetal-only birthweight	rs6575803	14	101257755	C	T	0.895	0.034	0.007	9.90E-07
fetal-only birthweight	rs7402983	15	99193276	A	C	0.405	0.027	0.004	4.60E-10
fetal-only birthweight	rs40434	16	55699525	G	A	0.391	0.017	0.004	4.80E-05
fetal-only birthweight	rs222857	17	7164563	T	C	0.575	0.026	0.004	5.80E-10
fetal-only birthweight	rs4511593	17	7455536	T	C	0.65	0.019	0.004	7.40E-06
fetal-only birthweight	rs9909342	17	25652275	A	G	0.381	0.019	0.004	6.70E-06
fetal-only birthweight	rs11867479	17	68090207	T	C	0.353	0.018	0.004	2.20E-05
fetal-only birthweight	rs10221267	17	68464662	T	C	0.512	0.018	0.004	1.90E-05
fetal-only birthweight	rs73354194	17	79905947	C	T	0.025	0.06	0.014	1.70E-05
fetal-only birthweight	rs8106042	19	7161849	G	C	0.281	0.023	0.005	6.60E-07
fetal-only birthweight	rs41355649	19	33790556	G	A	0.934	0.042	0.008	4.50E-07
fetal-only birthweight	rs1129156	19	40719076	T	C	0.268	0.022	0.005	1.90E-06
fetal-only birthweight	rs147957154	19	43431040	T	C	0.132	0.026	0.006	2.40E-05
fetal-only birthweight	rs11698914	20	31327144	C	G	0.233	0.029	0.005	2.80E-09

fetal-only birthweight	rs1012167	20	39159119	C	T	0.401	0.024	0.004	1.90E-08
fetal-only birthweight	rs753381	20	39797465	T	C	0.451	0.018	0.004	9.10E-06
fetal-only birthweight	rs6026449	20	57272617	C	T	0.627	0.018	0.004	3.20E-05
fetal-only birthweight	rs73143584	20	62445702	A	G	0.11	0.031	0.007	3.30E-06
fetal-only birthweight	rs134594	22	29468456	C	T	0.351	0.022	0.004	6.00E-07
fetal-only birthweight	rs41311445	22	42070374	A	C	0.903	0.034	0.007	1.30E-06
fetal-only birthweight	rs7285579	22	46441980	C	T	0.698	0.018	0.005	1.10E-04
maternal-only birthweight	rs17367504	1	11862778	G	A	0.167	0.032	0.006	2.50E-07
maternal-only birthweight	rs4952673	2	43423870	A	G	0.474	0.025	0.005	1.20E-07
maternal-only birthweight	rs9851257	3	123125711	T	A	0.744	0.027	0.005	1.10E-06
maternal-only birthweight	rs6440006	3	141142691	A	G	0.448	0.02	0.005	1.30E-05
maternal-only birthweight	rs2189234	4	106075498	G	T	0.616	0.026	0.005	7.30E-08
maternal-only birthweight	rs4579095	4	174726635	G	A	0.407	0.023	0.005	1.50E-06
maternal-only birthweight	rs2946179	5	157886627	C	T	0.735	0.045	0.005	3.70E-17
maternal-only birthweight	rs34471628	5	172196752	A	G	0.962	0.067	0.012	5.20E-08
maternal-only birthweight	rs9379084	6	7231843	G	A	0.883	0.04	0.007	4.70E-08
maternal-only birthweight	rs6911024	6	31368451	T	C	0.902	0.04	0.008	2.20E-07
maternal-only birthweight	rs2971669	7	44231778	T	C	0.219	0.028	0.006	4.90E-07
maternal-only birthweight	rs45446698	7	99332948	G	T	0.042	0.077	0.012	1.10E-10
maternal-only birthweight	rs3918226	7	150690176	C	T	0.919	0.04	0.009	7.40E-06
maternal-only birthweight	rs11778247	8	23403378	G	A	0.834	0.026	0.006	6.20E-05
maternal-only birthweight	rs3740360	10	96025491	C	A	0.114	0.044	0.007	1.60E-09
maternal-only birthweight	rs10734564	11	48160429	A	G	0.179	0.033	0.006	1.20E-07
maternal-only birthweight	rs10830963	11	92708710	G	C	0.279	0.046	0.005	4.60E-19

maternal-only birthweight	rs10895278	11	102095335	C	T	0.34	0.025	0.005	3.30E-07
maternal-only birthweight	rs11051061	12	30914668	A	G	0.268	0.026	0.005	1.10E-06
maternal-only birthweight	rs180438	12	47187260	G	A	0.195	0.039	0.006	4.10E-11
maternal-only birthweight	rs1533688	12	102772745	C	T	0.774	0.025	0.006	1.60E-05
maternal-only birthweight	rs17033114	12	103123339	T	C	0.934	0.053	0.01	6.20E-08
maternal-only birthweight	rs3184504	12	111884608	C	T	0.518	0.034	0.005	1.80E-13
maternal-only birthweight	rs2928148	15	41401550	A	G	0.523	0.02	0.005	1.60E-05
maternal-only birthweight	rs12909648	15	86224570	G	A	0.523	0.027	0.005	3.40E-09
maternal-only birthweight	rs12443252	15	91064690	T	C	0.55	0.023	0.005	1.70E-06
maternal-only birthweight	rs11641308	16	75312023	T	C	0.346	0.023	0.005	7.90E-06
maternal-only birthweight	rs2967676	19	8789666	A	C	0.842	0.048	0.006	4.40E-14
maternal-only birthweight	rs12461110	19	56320663	A	G	0.364	0.022	0.005	3.40E-06
maternal-only birthweight	rs304001	19	56423668	G	A	0.394	0.023	0.005	1.90E-06
maternal-only birthweight	rs181451002	20	32466219	G	A	0.979	0.059	0.016	3.10E-04

Table S3. Evidence for the relationships between the 52 candidate mediators and MI or AF from previous literature

Candidate mediators	Evidence from previous literature	PMID
Socioeconomic indicator		
Education<br>Household income<br>Occupational attainment<br>Townsend deprivation index	Low levels of education and wealth have been shown to be independent predictors of myocardial infarction (MI), and the risk of incident and prevalent atrial fibrillation (AF) has been reported to be greater in individuals with lower levels of education, income and employment.	34079095; 26585783
Psychological well-being		
Positive affect<br>Life satisfaction<br>Neuroticism<br>Depressive symptoms	In a prospective cohort study, individuals who were rated by trained observers as displaying a more positive affect had a 22% lower risk of incident coronary heart disease (HR, 0.78 [95% CI, 0.63–0.96]).<br>Negative psychological factors, personality traits, and mental health disorders can affect cardiovascular health. A meta-analysis from 2014 including 893 850 participants showed that depression was associated with an increased risk of MI (RR, 1.30 [95% CI, 1.22–1.40]).	33486973
Lifestyle behaviours		
Cigarettes smoked per day<br>Alcoholic drinks per week<br>Coffee consumption<br>Long sleep<br>Short sleep<br>Chronotype<br>MPA (device-measured)	Cigarette smoking, diet, physical activity, and sleep are components of cardiovascular health (CVH) proposed by the American Heart Association.	35766027
Body composition and strength		
Childhood obesity	In a cohort of children born in Denmark a higher body mass index (BMI) in childhood was associated with increased risk for cardiovascular disease (CVD) in adulthood.	25810456
BMI<br>WC<br>WHR	Higher BMI, waist circumference (WC), and waist-to-hip ratio (WHR) were associated with a greater risk of MI and AF.	33882682
Height	A Korean cohort study of 16,528,128 individuals found that shorter height in adulthood was strongly related to an increased risk of MI. Additionally, there is a primary association between a genetically determined shorter height and an increased risk of coronary artery disease in European.<br>The Cardiovascular Health Study consisted of 5,860 US	29025084; 25853659; 22977225

	adults showed that increased height is significantly associated with the risk of AF, independently from sex.	
Appendicular lean mass	The ATTICA study of 1,019 participants aged 45 years or older showed that individuals in the highest tertile of skeletal muscle mass measured by appendicular lean mass standardized by BMI had a 81% lower risk for 10-year cardiovascular disease compared with those in the lowest tertile.<br>Greater lean body mass is a strong independent risk factor for AF. After adjusting for obesity-related risk factors, the risk of AF conferred by higher BMI is primarily driven by the association between lean body mass and AF.	31712252; 26371115
Grip strength	A cohort of 502,635 individuals from the UK Biobank reported that grip strength showed inverse associations with incident coronary heart disease and AF.	29632216
Cardiometabolic traits		
**Blood pressure**		
Hypertension	Blood pressure is one component of the concept of CVH. Elevated blood pressure is a major risk factor for both AF and MI. In the Systolic Blood Pressure Intervention Trial trial, intensive blood pressure lowering led to a significant reduction in the risk of new-onset AF (HR 0.74, 95% CI 0.56–0.98) and a composite outcome including MI, acute coronary syndrome, stroke, HF or death from cardiovascular disease (HR 0.75, 95% CI 0.64–0.89).	26551272; 32362229
SBP		
DBP		
**Glucose metabolism**		
Type 2 diabetes	Blood glucose is one component of the concept of CVH.	35766027
Fasting glucose		
2-h glucose		
HbA1c		
Fasting insulin		
**Lipid traits**		
Total cholesterol	Blood lipids is one component of the concept of CVH.	35766027
HDL-C		
LDL-C		
Triglycerides		
ApoA-I		

ApoB		
**Metabolites**		
Omega-3 fatty acids<br>Omega-6 fatty acids<br>DHA<br>Linoleic acid	Previous metanalyses of classical observational studies indicate that higher circulating long-chain omega-3 and omega-6 polyunsaturated fatty acids are not associated with lower risk of cerebrovascular disease. However, a series of systematic reviews of RCTs with the overall conclusion that increasing omega-3, omega-6 or total polyunsaturated fatty acids intake, via supplementation or diet, has modest to no effect on CVD events.	23112118; 30484282
Isoleucine<br>Leucine<br>Valine	A meta-analysis indicated that elevated concentrations of circulating Isoleucine were associated with increased risks of CVD, independent of traditional risk factors.<br>The Women's Health Study prospective cohort showed that circulating plasma branched-chain amino acids were positively associated with incident CVD in women.	35576716; 29572205
Phenylalanine<br>Tyrosine<br>Alanine<br>Glutamine	A 12-year follow-up study of 9,584 Finnish men showed alanine, phenylalanine, tyrosine, and glutamine were associated with CVD.	34346487
Glycine	An observational study of 4109 participants reported that plasma glycine was inversely associated with risk of acute myocardial infarction in patients with suspected stable angina pectoris.	26722126
**Kidney function**		
CKD<br>eGFR<br>UACR	Chronic kidney disease (CKD) is a major risk factor for MI and AF. The incidence and prevalence of cardiovascular events is significantly higher in patients with early CKD stages compared with the general population.<br>A meta-analysis of three cohorts indicated that reduced eGFR and elevated urine albumin-to-creatinine ratio (UACR) were significantly associated with greater risk of incident AF.	33720773; 28798221
**Inflammation marker**		
CRP	C-reactive protein (CRP), a marker of systemic inflammation, has been evaluated as a risk predictor for future MI.<br>CRP is elevated in AF patients.	10733371; 11739301; 9077376

Abbreviations: AF=atrial fibrillation; ApoA-I=Apolipoprotein A-I; ApoB=Apolipoprotein B; BMI=body mass index; CKD=Chronic kidney disease; CI=confidence interval; CRP=C-

reactive protein; CVH=cardiovascular health; DBP=diastolic blood pressure; DHA=docosahexaenoic acid; eGFR=estimated glomerular filtration rate; HbA1c=Glycated hemoglobin; HDL-C=high-density lipoprotein cholesterol; HR=hazard ratio; LDL-C=low-density lipoprotein cholesterol; MI=myocardial infarction; MR=Mendelian randomization; SBP=systolic blood pressure; UACR=urinary albumin-to-creatinine ratio; WC=Waist circumference; WHR=waist-to-hip ratio.

Table S4. UVMR pleiotropy and heterogeneity test for the causal associations of birthweight with MI and AF

Outcome	Exposure	Pleiotropy test			Heterogeneity test		
		**Egger_intercept**	**Intercept_se**	**$P_{intercept}$**	**Q statistic**	**Q_df**	**$P_{heterogeneity}$**
MI	Birthweight	-0.005	0.004	2.32E-01	477	120	4.51E-44
	Fetal-specific birthweight	-0.001	0.005	8.05E-01	124	49	1.89E-08
	Maternal-specific birthweight	-0.005	0.014	7.21E-01	179	28	5.02E-24
AF	Birthweight	-0.004	0.003	2.43E-01	378	122	5.08E-28
	Fetal-specific birthweight	-0.007	0.005	2.16E-01	150	50	5.81E-12
	Maternal-specific birthweight	0.015	0.008	7.26E-02	79	28	8.59E-07

Abbreviations: AF=atrial fibrillation; MI=myocardial infarction; UVMR=univariable Mendelian randomization.

Table S5. UVMR estimates for the causal associations between birthweight and candidate mediators

Exposure	Outcome	Method	No. of SNP	F-statistic	β (95% CI)[a]	OR (95% CI)[b]	P value	FDR q-value
Association between higher birthweight and candidate mediator								
Birthweight	Education	IVW	122	52	0.046 (0.017, 0.075)	/	2.70E-03	6.50E-03
		MR Egger			0.055 (-0.016, 0.126)	/	1.29E-01	/
		Simple mode			0.053 (-0.010, 0.116)	/	1.08E-01	/
		Weighted median			0.038 (0.009, 0.067)	/	9.35E-03	/
		Weighted mode			0.031 (-0.014, 0.076)	/	1.87E-01	/
		MR PRESSO			0.035 (0.011, 0.059)	/	4.30E-03	/
Birthweight	Household income	IVW	123	52	0.047 (0.012, 0.082)	/	9.10E-03	1.68E-02
		MR Egger			0.050 (-0.030, 0.130)	/	2.26E-01	/
		Simple mode			0.049 (-0.061, 0.159)	/	3.91E-01	/
		Weighted median			0.049 (0.010, 0.088)	/	1.69E-02	/
		Weighted mode			0.015 (-0.075, 0.105)	/	7.45E-01	/
		MR PRESSO			0.042 (0.009, 0.075)	/	1.40E-02	/
Birthweight	Occupational attainment	IVW	104	51	0.164 (0.076, 0.252)	/	2.49E-04	1.02E-03
		MR Egger			0.161 (-0.035, 0.357)	/	1.11E-01	/
		Simple mode			0.204 (-0.037, 0.445)	/	1.00E-01	/
		Weighted median			0.145 (0.033, 0.257)	/	1.11E-02	/
		Weighted mode			0.125 (-0.063, 0.313)	/	1.94E-01	/
		MR PRESSO			0.151 (0.065, 0.237)	/	7.63E-04	/
Birthweight	Townsend deprivation index	IVW	123	52	-0.019 (-0.043, 0.005)	/	1.06E-01	1.48E-01
		MR Egger			-0.001 (-0.052, 0.050)	/	9.56E-01	/

		Simple mode			-0.040 (-0.113, 0.033)	/	2.77E-01	/
		Weighted median			-0.026 (-0.055, 0.003)	/	9.42E-02	/
		Weighted mode			-0.024 (-0.085, 0.037)	/	4.45E-01	/
		MR PRESSO			-0.020 (-0.042, 0.002)	/	6.42E-02	/
Birthweight	Positive affect	IVW	96	52	0.002 (-0.020, 0.024)	1.00 (0.98, 1.02)	8.67E-01	9.19E-01
		MR Egger			0.000 (-0.053, 0.053)	1.00 (0.95, 1.05)	9.88E-01	/
		Simple mode			0.005 (-0.044, 0.054)	1.01 (0.96, 1.06)	8.30E-01	/
		Weighted median			-0.007 (-0.031, 0.017)	0.99 (0.97, 1.02)	5.45E-01	/
		Weighted mode			-0.008 (-0.041, 0.025)	0.99 (0.96, 1.03)	6.30E-01	/
		MR PRESSO			0.006 (-0.014, 0.026)	1.01 (0.99, 1.03)	5.19E-01	/
Birthweight	Life satisfaction	IVW	96	52	-0.001 (-0.023, 0.021)	1.00 (0.98, 1.02)	9.34E-01	9.34E-01
		MR Egger			-0.012 (-0.069, 0.045)	0.99 (0.93, 1.05)	6.76E-01	/
		Simple mode			0.009 (-0.042, 0.060)	1.01 (0.96, 1.06)	7.24E-01	/
		Weighted median			-0.007 (-0.032, 0.018)	0.99 (0.97, 1.02)	5.82E-01	/
		Weighted mode			-0.005 (-0.040, 0.030)	1.00 (0.96, 1.03)	7.96E-01	/
		MR PRESSO			0.003 (-0.017, 0.023)	1.00 (0.98, 1.02)	7.70E-01	/
Birthweight	Neuroticism	IVW	96	52	0.004 (-0.021, 0.029)	1.00 (0.98, 1.03)	7.93E-01	8.94E-01
		MR Egger			0.002 (-0.065, 0.069)	1.00 (0.94, 1.07)	9.61E-01	/
		Simple mode			0.012 (-0.049, 0.073)	1.01 (0.95, 1.08)	7.07E-01	/
		Weighted median			-0.010 (-0.037, 0.017)	0.99 (0.96, 1.02)	5.08E-01	/
		Weighted mode			-0.013 (-0.060, 0.034)	0.99 (0.94, 1.03)	5.88E-01	/
		MR PRESSO			0.000 (-0.024, 0.024)	1.00 (0.98, 1.02)	9.99E-01	/
Birthweight	Depressive symptoms	IVW	96	52	0.002 (-0.016, 0.020)	1.00 (0.98, 1.02)	8.46E-01	9.15E-01
		MR Egger			0.001 (-0.040, 0.042)	1.00 (0.96, 1.04)	9.79E-01	/

		Simple mode			0.005 (-0.036, 0.046)	1.01 (0.96, 1.05)	8.03E-01	/
		Weighted median			-0.003 (-0.021, 0.015)	1.00 (0.98, 1.02)	7.09E-01	/
		Weighted mode			-0.006 (-0.035, 0.023)	0.99 (0.97, 1.02)	6.96E-01	/
		MR PRESSO			-0.004 (-0.020, 0.012)	1.00 (0.98, 1.01)	5.78E-01	/
Birthweight	Cigarettes smoked per day	IVW	118	52	0.031 (-0.034, 0.096)	/	3.37E-01	4.47E-01
		MR Egger			-0.038 (-0.185, 0.109)	/	6.14E-01	/
		Simple mode			-0.002 (-0.180, 0.176)	/	9.84E-01	/
		Weighted median			0.025 (-0.055, 0.105)	/	5.44E-01	/
		Weighted mode			0.008 (-0.114, 0.130)	/	8.95E-01	/
		MR PRESSO			0.039 (-0.024, 0.102)	/	2.22E-01	/
Birthweight	Alcoholic drinks per week	IVW	117	52	0.025 (-0.004, 0.054)	/	9.18E-02	1.31E-01
		MR Egger			0.019 (-0.048, 0.086)	/	5.66E-01	/
		Simple mode			0.040 (-0.025, 0.105)	/	2.30E-01	/
		Weighted median			0.022 (-0.007, 0.051)	/	1.58E-01	/
		Weighted mode			0.021 (-0.020, 0.062)	/	3.14E-01	/
		MR PRESSO			0.022 (-0.002, 0.046)	/	7.56E-02	/
Birthweight	Coffee consumption	IVW	122	50	0.052 (0.025, 0.079)	/	1.41E-04	8.30E-04
		MR Egger			0.109 (0.044, 0.174)	/	1.26E-03	/
		Simple mode			0.039 (-0.022, 0.100)	/	2.08E-01	/
		Weighted median			0.039 (0.014, 0.064)	/	1.85E-03	/
		Weighted mode			0.015 (-0.038, 0.068)	/	5.76E-01	/
		MR PRESSO			0.055 (0.035, 0.075)	/	1.86E-07	/
Birthweight	Long sleep (≥9h per night)	IVW	123	52	-0.001 (-0.009, 0.007)	1.00 (0.99, 1.01)	7.21E-01	8.31E-01
		MR Egger			0.004 (-0.012, 0.020)	1.00 (0.99, 1.02)	6.01E-01	/

		Simple mode			0.000 (-0.024, 0.024)	1.00 (0.98, 1.02)	9.93E-01	/
		Weighted median			-0.001 (-0.011, 0.009)	1.00 (0.99, 1.01)	8.87E-01	/
		Weighted mode			0.002 (-0.018, 0.022)	1.00 (0.98, 1.02)	8.77E-01	/
		MR PRESSO			-0.003 (-0.011, 0.005)	1.00 (0.99, 1.01)	3.28E-01	/
Birthweight	Short sleep (<7h per night)	IVW	123	52	0.001 (-0.011, 0.013)	1.00 (0.99, 1.01)	9.21E-01	9.34E-01
		MR Egger			0.004 (-0.023, 0.031)	1.00 (0.98, 1.03)	7.64E-01	/
		Simple mode			0.000 (-0.033, 0.033)	1.00 (0.97, 1.03)	9.92E-01	/
		Weighted median			-0.002 (-0.016, 0.012)	1.00 (0.98, 1.01)	7.99E-01	/
		Weighted mode			-0.003 (-0.028, 0.022)	1.00 (0.97, 1.02)	8.32E-01	/
		MR PRESSO			0.000 (-0.012, 0.012)	1.00 (0.99, 1.01)	9.37E-01	/
Birthweight	Chronotype	IVW	123	52	-0.009 (-0.038, 0.020)	/	5.50E-01	6.78E-01
		MR Egger			0.039 (-0.026, 0.104)	/	2.40E-01	/
		Simple mode			-0.002 (-0.075, 0.071)	/	9.64E-01	/
		Weighted median			-0.003 (-0.034, 0.028)	/	8.56E-01	/
		Weighted mode			0.005 (-0.044, 0.054)	/	8.42E-01	/
		MR PRESSO			-0.005 (-0.030, 0.020)	/	6.80E-01	/
Birthweight	MPA (device-measured)	IVW	123	52	-0.423 (-0.811, -0.035)	/	3.27E-02	5.10E-02
		MR Egger			-1.093 (-1.973, -0.213)	/	1.63E-02	/
		Simple mode			-0.662 (-1.865, 0.541)	/	2.83E-01	/
		Weighted median			-0.824 (-1.345, -0.303)	/	1.90E-03	/
		Weighted mode			-1.120 (-2.067, -0.173)	/	2.20E-02	/
		MR PRESSO			-0.478 (-0.850, -0.106)	/	1.31E-02	/
Birthweight	Childhood obesity	IVW	77	53	0.525 (0.198, 0.852)	1.69 (1.22, 2.34)	1.71E-03	4.77E-03
		MR Egger			-0.233 (-1.023, 0.557)	0.79 (0.36, 1.75)	5.65E-01	/

		Simple mode			0.016 (-0.911, 0.943)	1.02 (0.40, 2.57)	9.72E-01	/
		Weighted median			0.153 (-0.323, 0.629)	1.17 (0.72, 1.88)	5.31E-01	/
		Weighted mode			0.054 (-0.656, 0.764)	1.06 (0.52, 2.15)	8.82E-01	/
		MR-PRESSO			0.525 (0.198, 0.852)	1.69 (1.22, 2.34)	2.43E-03	/
Birthweight	BMI	IVW	90	53	0.123 (0.062, 0.184)	/	8.88E-05	6.72E-04
		MR Egger			0.190 (0.039, 0.341)	/	1.57E-02	/
		Simple mode			0.036 (-0.095, 0.167)	/	5.98E-01	/
		Weighted median			0.080 (0.017, 0.143)	/	1.25E-02	/
		Weighted mode			0.078 (-0.004, 0.160)	/	6.70E-02	/
		MR PRESSO			0.117 (0.066, 0.168)	/	2.00E-05	/
Birthweight	WC	IVW	90	53	0.182 (0.117, 0.247)	/	4.11E-08	4.36E-07
		MR Egger			0.349 (0.194, 0.504)	/	3.06E-05	/
		Simple mode			0.293 (0.140, 0.446)	/	2.81E-04	/
		Weighted median			0.247 (0.176, 0.318)	/	5.06E-12	/
		Weighted mode			0.270 (0.178, 0.362)	/	1.25E-07	/
		MR-PRESSO			0.194 (0.137, 0.251)	/	1.54E-09	/
Birthweight	WHR	IVW	90	53	0.004 (-0.063, 0.071)	/	8.99E-01	9.34E-01
		MR Egger			0.210 (0.053, 0.367)	/	1.01E-02	/
		Simple mode			0.137 (-0.010, 0.284)	/	7.00E-02	/
		Weighted median			0.042 (-0.027, 0.111)	/	2.28E-01	/
		Weighted mode			0.055 (-0.051, 0.161)	/	3.13E-01	/
		MR-PRESSO			-0.007 (-0.062, 0.048)	/	8.06E-01	/
Birthweight	Height	IVW	89	53	0.457 (0.318, 0.596)	/	9.53E-11	1.68E-09
		MR Egger			0.442 (0.103, 0.781)	/	1.25E-02	/

		Simple mode			0.228 (0.093, 0.363)	/	1.44E-03	/
		Weighted median			0.203 (0.132, 0.274)	/	2.59E-08	/
		Weighted mode			0.152 (0.068, 0.236)	/	5.77E-04	/
		MR PRESSO			0.342 (0.268, 0.416)	/	4.87E-13	/
Birthweight	Appendicular lean mass	IVW	123	52	0.488 (0.372, 0.604)	/	2.06E-16	1.09E-14
		MR Egger			0.363 (0.098, 0.628)	/	8.31E-03	/
		Simple mode			0.262 (0.174, 0.350)	/	5.10E-08	/
		Weighted median			0.132 (0.081, 0.183)	/	3.67E-07	/
		Weighted mode			0.116 (0.065, 0.167)	/	1.53E-05	/
		MR PRESSO			0.434 (0.373, 0.495)	/	6.62E-20	/
Birthweight	Grip strength	IVW	123	52	0.117 (0.088, 0.146)	/	3.06E-14	8.11E-13
		MR Egger			0.084 (0.015, 0.153)	/	1.91E-02	/
		Simple mode			0.060 (-0.011, 0.131)	/	1.04E-01	/
		Weighted median			0.070 (0.043, 0.097)	/	4.52E-07	/
		Weighted mode			0.024 (-0.013, 0.061)	/	2.14E-01	/
		MR-PRESSO			0.122 (0.097, 0.147)	/	2.13E-15	/
Birthweight	Hypertension	IVW	119	53	-0.056 (-0.254, 0.142)	0.95 (0.78, 1.15)	5.77E-01	6.95E-01
		MR Egger			0.369 (-0.111, 0.849)	1.45 (0.89, 2.34)	1.34E-01	/
		Simple mode			-0.078 (-0.429, 0.273)	0.92 (0.65, 1.31)	6.64E-01	/
		Weighted median			0.000 (-0.161, 0.161)	1.00 (0.85, 1.17)	1.00E+00	/
		Weighted mode			-0.039 (-0.337, 0.259)	0.96 (0.71, 1.30)	8.00E-01	/
		MR PRESSO			-0.043 (-0.170, 0.084)	0.96 (0.84, 1.09)	5.13E-01	/
Birthweight	SBP	IVW	116	50	-2.305 (-3.810, -0.800)	/	2.67E-03	6.50E-03
		MR Egger			0.823 (-2.840, 4.486)	/	6.61E-01	/

		Simple mode			-1.494 (-3.717, 0.729)	/	1.91E-01	/
		Weighted median			-0.801 (-1.497, -0.105)	/	2.42E-02	/
		Weighted mode			0.188 (-1.255, 1.631)	/	7.99E-01	/
		MR PRESSO			-1.841 (-2.549, -1.133)	/	2.22E-06	/
Birthweight	DBP	IVW	117	50	-0.915 (-1.848, 0.018)	/	5.48E-02	8.07E-02
		MR Egger			0.255 (-2.028, 2.538)	/	8.27E-01	/
		Simple mode			-0.622 (-2.063, 0.819)	/	3.99E-01	/
		Weighted median			-0.334 (-0.738, 0.070)	/	1.05E-01	/
		Weighted mode			0.238 (-0.660, 1.136)	/	6.05E-01	/
		MR PRESSO			-0.851 (-1.231, -0.471)	/	3.40E-05	/
Birthweight	Type 2 diabetes	IVW	84	52	-0.487 (-0.928, -0.046)	0.61 (0.40, 0.96)	3.02E-02	4.85E-02
		MR Egger			-1.844 (-2.908, -0.780)	0.16 (0.05, 0.46)	1.06E-03	/
		Simple mode			-0.466 (-0.870, -0.062)	0.63 (0.42, 0.94)	2.59E-02	/
		Weighted median			-0.474 (-0.654, -0.294)	0.62 (0.52, 0.75)	2.48E-07	/
		Weighted mode			-0.466 (-0.725, -0.207)	0.63 (0.48, 0.81)	6.66E-04	/
		MR-PRESSO			-0.343 (-0.492, -0.194)	0.71 (0.61, 0.82)	2.27E-05	/
Birthweight	Fasting glucose	IVW	122	52	-0.092 (-0.166, -0.018)	/	1.50E-02	2.48E-02
		MR Egger			-0.100 (-0.276, 0.076)	/	2.66E-01	/
		Simple mode			-0.013 (-0.080, 0.054)	/	7.05E-01	/
		Weighted median			-0.050 (-0.081, -0.019)	/	1.69E-03	/
		Weighted mode			-0.037 (-0.096, 0.022)	/	2.21E-01	/
		MR PRESSO			-0.062 (-0.086, -0.038)	/	1.64E-06	/
Birthweight	2-h glucose	IVW	123	52	-0.208 (-0.351, -0.065)	/	4.63E-03	1.02E-02
		MR Egger			-0.400 (-0.733, -0.067)	/	2.00E-02	/

		Simple mode			-0.245 (-0.600, 0.110)	/	1.79E-01	/
		Weighted median			-0.221 (-0.360, -0.082)	/	1.84E-03	/
		Weighted mode			-0.245 (-0.680, 0.190)	/	2.71E-01	/
		MR PRESSO			-0.185 (-0.285, -0.085)	/	4.49E-04	/
Birthweight	HbA1c	IVW	123	52	-0.049 (-0.084, -0.014)	/	5.94E-03	1.21E-02
		MR Egger			-0.080 (-0.162, 0.002)	/	5.85E-02	/
		Simple mode			-0.031 (-0.080, 0.018)	/	2.16E-01	/
		Weighted median			-0.033 (-0.057, -0.009)	/	3.99E-03	/
		Weighted mode			-0.020 (-0.053, 0.013)	/	2.34E-01	/
		MR PRESSO			-0.032 (-0.048, -0.016)	/	2.38E-04	/
Birthweight	Fasting insulin	IVW	123	52	-0.102 (-0.137, -0.067)	/	2.40E-08	3.18E-07
		MR Egger			-0.061 (-0.145, 0.023)	/	1.59E-01	/
		Simple mode			-0.033 (-0.119, 0.053)	/	4.58E-01	/
		Weighted median			-0.108 (-0.141, -0.075)	/	6.02E-10	/
		Weighted mode			-0.156 (-0.223, -0.089)	/	1.44E-05	/
		MR PRESSO			-0.104 (-0.131, -0.077)	/	6.27E-12	/
Birthweight	Total cholesterol	IVW	86	52	-0.181 (-0.291, -0.071)	/	1.31E-03	4.34E-03
		MR Egger			-0.003 (-0.273, 0.267)	/	9.80E-01	/
		Simple mode			-0.072 (-0.213, 0.069)	/	3.17E-01	/
		Weighted median			-0.111 (-0.189, -0.033)	/	5.89E-03	/
		Weighted mode			-0.072 (-0.158, 0.014)	/	1.01E-01	/
		MR PRESSO			-0.152 (-0.217, -0.087)	/	1.33E-05	/
Birthweight	HDL-C	IVW	86	52	-0.022 (-0.108, 0.064)	/	6.24E-01	7.35E-01
		MR Egger			-0.278 (-0.482, -0.074)	/	9.19E-03	/

		Simple mode			-0.042 (-0.201, 0.117)	/	6.10E-01	/
		Weighted median			-0.044 (-0.126, 0.038)	/	3.02E-01	/
		Weighted mode			-0.025 (-0.164, 0.114)	/	7.29E-01	/
		MR-PRESSO			0.020 (-0.045, 0.085)	/	5.51E-01	/
Birthweight	LDL-C	IVW	85	52	-0.139 (-0.243, -0.035)	/	9.19E-03	1.68E-02
		MR Egger			0.083 (-0.172, 0.338)	/	5.24E-01	/
		Simple mode			-0.044 (-0.232, 0.144)	/	6.49E-01	/
		Weighted median			-0.025 (-0.105, 0.055)	/	5.33E-01	/
		Weighted mode			0.004 (-0.086, 0.094)	/	9.24E-01	/
		MR PRESSO			-0.097 (-0.158, -0.036)	/	2.74E-03	/
Birthweight	Triglycerides	IVW	85	52	-0.127 (-0.205, -0.049)	/	1.69E-03	4.77E-03
		MR Egger			0.149 (-0.035, 0.333)	/	1.19E-01	/
		Simple mode			-0.021 (-0.242, 0.200)	/	8.53E-01	/
		Weighted median			-0.064 (-0.148, 0.020)	/	1.33E-01	/
		Weighted mode			0.033 (-0.108, 0.174)	/	6.48E-01	/
		MR-PRESSO			-0.141 (-0.204, -0.078)	/	2.92E-05	/
Birthweight	ApoA-I	IVW	122	52	-0.013 (-0.125, 0.099)	/	8.22E-01	9.08E-01
		MR Egger			-0.202 (-0.463, 0.059)	/	1.32E-01	/
		Simple mode			-0.140 (-0.493, 0.213)	/	4.37E-01	/
		Weighted median			-0.070 (-0.219, 0.079)	/	3.51E-01	/
		Weighted mode			-0.105 (-0.352, 0.142)	/	4.09E-01	/
		MR PRESSO			-0.025 (-0.135, 0.085)	/	6.62E-01	/
Birthweight	ApoB	IVW	123	52	-0.198 (-0.302, -0.094)	/	1.95E-04	9.36E-04
		MR Egger			-0.108 (-0.349, 0.133)	/	3.81E-01	/

		Simple mode			-0.066 (-0.364, 0.232)	/	6.67E-01	/
		Weighted median			-0.145 (-0.292, 0.002)	/	5.44E-02	/
		Weighted mode			-0.050 (-0.271, 0.171)	/	6.58E-01	/
		MR PRESSO			-0.164 (-0.260, -0.068)	/	1.13E-03	/
Birthweight	Omega-3 fatty acids	IVW	123	52	-0.085 (-0.146, -0.024)	/	6.83E-03	1.34E-02
		MR Egger			-0.007 (-0.146, 0.132)	/	9.17E-01	/
		Simple mode			-0.193 (-0.365, -0.021)	/	2.95E-02	/
		Weighted median			-0.022 (-0.085, 0.041)	/	4.97E-01	/
		Weighted mode			-0.029 (-0.166, 0.108)	/	6.79E-01	/
		MR PRESSO			-0.050 (-0.095, -0.005)	/	3.08E-02	/
Birthweight	Omega-6 fatty acids	IVW	123	52	-0.109 (-0.178, -0.040)	/	1.67E-03	4.77E-03
		MR Egger			-0.087 (-0.242, 0.068)	/	2.73E-01	/
		Simple mode			-0.036 (-0.197, 0.125)	/	6.60E-01	/
		Weighted median			-0.089 (-0.152, -0.026)	/	5.88E-03	/
		Weighted mode			-0.074 (-0.164, 0.016)	/	1.08E-01	/
		MR PRESSO			-0.106 (-0.157, -0.055)	/	1.09E-04	/
Birthweight	DHA	IVW	123	52	-0.040 (-0.091, 0.011)	/	1.20E-01	1.63E-01
		MR Egger			-0.019 (-0.135, 0.097)	/	7.52E-01	/
		Simple mode			0.046 (-0.095, 0.187)	/	5.24E-01	/
		Weighted median			-0.027 (-0.084, 0.030)	/	3.57E-01	/
		Weighted mode			0.011 (-0.085, 0.107)	/	8.20E-01	/
		MR PRESSO			-0.025 (-0.068, 0.018)	/	2.63E-01	/
Birthweight	Linoleic acid	IVW	123	52	-0.093 (-0.158, -0.028)	/	4.81E-03	1.02E-02
		MR Egger			-0.073 (-0.220, 0.074)	/	3.38E-01	/

		Simple mode			-0.083 (-0.240, 0.074)	/	2.98E-01	/
		Weighted median			-0.081 (-0.144, -0.018)	/	1.20E-02	/
		Weighted mode			-0.083 (-0.181, 0.015)	/	9.52E-02	/
		MR PRESSO			-0.087 (-0.140, -0.034)	/	1.61E-03	/
Birthweight	Isoleucine	IVW	123	52	-0.077 (-0.122, -0.032)	/	9.45E-04	3.34E-03
		MR Egger			-0.066 (-0.172, 0.040)	/	2.20E-01	/
		Simple mode			-0.127 (-0.254, 0.000)	/	5.42E-02	/
		Weighted median			-0.103 (-0.164, -0.042)	/	1.12E-03	/
		Weighted mode			-0.103 (-0.195, -0.011)	/	2.94E-02	/
		MR PRESSO			-0.080 (-0.123, -0.037)	/	3.19E-04	/
Birthweight	Leucine	IVW	123	52	-0.087 (-0.136, -0.038)	/	5.71E-04	2.16E-03
		MR Egger			-0.110 (-0.224, 0.004)	/	6.08E-02	/
		Simple mode			-0.001 (-0.132, 0.130)	/	9.85E-01	/
		Weighted median			-0.060 (-0.117, -0.003)	/	3.90E-02	/
		Weighted mode			-0.006 (-0.110, 0.098)	/	9.04E-01	/
		MR PRESSO			-0.079 (-0.122, -0.036)	/	4.79E-04	/
Birthweight	Valine	IVW	123	52	-0.083 (-0.140, -0.026)	/	4.76E-03	1.02E-02
		MR Egger			-0.072 (-0.203, 0.059)	/	2.84E-01	/
		Simple mode			-0.191 (-0.332, -0.050)	/	8.98E-03	/
		Weighted median			-0.084 (-0.149, -0.019)	/	9.85E-03	/
		Weighted mode			-0.004 (-0.114, 0.106)	/	9.43E-01	/
		MR PRESSO			-0.097 (-0.142, -0.052)	/	3.99E-05	/
Birthweight	Phenylalanine	IVW	123	52	-0.071 (-0.118, -0.024)	/	2.67E-03	6.50E-03
		MR Egger			-0.100 (-0.206, 0.006)	/	6.76E-02	/

		Simple mode			-0.053 (-0.194, 0.088)	/	4.64E-01	/
		Weighted median			-0.057 (-0.118, 0.004)	/	6.09E-02	/
		Weighted mode			-0.069 (-0.175, 0.037)	/	2.02E-01	/
		MR PRESSO			-0.072 (-0.115, -0.029)	/	1.15E-03	/
Birthweight	Tyrosine	IVW	123	52	-0.100 (-0.153, -0.047)	/	2.12E-04	9.36E-04
		MR Egger			-0.062 (-0.184, 0.060)	/	3.19E-01	/
		Simple mode			-0.119 (-0.282, 0.044)	/	1.54E-01	/
		Weighted median			-0.075 (-0.138, -0.012)	/	1.82E-02	/
		Weighted mode			-0.081 (-0.199, 0.037)	/	1.81E-01	/
		MR PRESSO			-0.089 (-0.138, -0.040)	/	6.49E-04	/
Birthweight	Alanine	IVW	123	52	-0.116 (-0.177, -0.055)	/	2.09E-04	9.36E-04
		MR Egger			-0.179 (-0.318, -0.040)	/	1.36E-02	/
		Simple mode			0.008 (-0.157, 0.173)	/	9.26E-01	/
		Weighted median			-0.047 (-0.110, 0.016)	/	1.50E-01	/
		Weighted mode			0.036 (-0.068, 0.140)	/	5.02E-01	/
		MR PRESSO			-0.084 (-0.133, -0.035)	/	9.40E-04	/
Birthweight	Glutamine	IVW	123	52	0.026 (-0.054, 0.106)	/	5.26E-01	6.64E-01
		MR Egger			-0.010 (-0.196, 0.176)	/	9.15E-01	/
		Simple mode			-0.013 (-0.187, 0.161)	/	8.86E-01	/
		Weighted median			0.021 (-0.044, 0.086)	/	5.21E-01	/
		Weighted mode			-0.039 (-0.151, 0.073)	/	4.90E-01	/
		MR PRESSO			0.061 (0.006, 0.116)	/	3.07E-02	/
Birthweight	Glycine	IVW	123	52	0.116 (0.059, 0.173)	/	5.39E-05	4.76E-04
		MR Egger			0.073 (-0.056, 0.202)	/	2.70E-01	/

		Simple mode			0.100 (-0.065, 0.265)	/	2.33E-01	/
		Weighted median			0.067 (0.004, 0.130)	/	3.31E-02	/
		Weighted mode			0.043 (-0.063, 0.149)	/	4.23E-01	/
		MR PRESSO			0.104 (0.055, 0.153)	/	7.43E-05	/
Birthweight	CKD	IVW	122	52	-0.134 (-0.261, -0.007)	0.87 (0.77, 0.99)	3.88E-02	5.88E-02
		MR Egger			-0.327 (-0.631, -0.023)	0.72 (0.53, 0.98)	3.74E-02	/
		Simple mode			-0.119 (-0.437, 0.199)	0.89 (0.65, 1.22)	4.62E-01	/
		Weighted median			-0.139 (-0.280, 0.002)	0.87 (0.76, 1.00)	5.29E-02	/
		Weighted mode			-0.095 (-0.340, 0.150)	0.91 (0.71, 1.16)	4.46E-01	/
		MR PRESSO			-0.140 (-0.250, -0.030)	0.87 (0.78, 0.97)	1.36E-02	/
Birthweight	eGFR	IVW	123	52	0.003 (-0.005, 0.011)	/	4.68E-01	6.05E-01
		MR Egger			0.017 (-0.001, 0.035)	/	7.19E-02	/
		Simple mode			-0.008 (-0.022, 0.006)	/	2.82E-01	/
		Weighted median			0.009 (0.003, 0.015)	/	2.64E-03	/
		Weighted mode			0.012 (0.004, 0.020)	/	2.95E-03	/
		MR PRESSO			0.002 (-0.004, 0.008)	/	5.48E-01	/
Birthweight	UACR	IVW	122	52	-0.065 (-0.098, -0.032)	/	1.12E-04	7.42E-04
		MR Egger			-0.041 (-0.117, 0.035)	/	2.92E-01	/
		Simple mode			-0.074 (-0.160, 0.012)	/	9.83E-02	/
		Weighted median			-0.046 (-0.077, -0.015)	/	4.49E-03	/
		Weighted mode			-0.031 (-0.092, 0.030)	/	3.09E-01	/
		MR PRESSO			-0.072 (-0.097, -0.047)	/	3.50E-07	/
Birthweight	CRP	IVW	107	52	-0.082 (-0.145, -0.019)	/	1.02E-02	1.80E-02
		MR Egger			-0.064 (-0.217, 0.089)	/	4.18E-01	/

		Simple mode			-0.084 (-0.164, -0.004)	/	4.23E-02	/
		Weighted median			-0.079 (-0.114, -0.044)	/	1.42E-05	/
		Weighted mode			-0.104 (-0.157, -0.051)	/	2.68E-04	/
		MR PRESSO			-0.082 (-0.115, -0.049)	/	4.52E-06	/
Association between higher fetal-specific birthweight and candidate mediator								
Fetal-specific birthweight	Education	IVW	51	33	0.033 (-0.006, 0.072)	/	1.04E-01	2.04E-01
		MR Egger			0.063 (-0.041, 0.167)	/	2.38E-01	/
		Simple mode			0.034 (-0.037, 0.105)	/	3.47E-01	/
		Weighted median			0.020 (-0.015, 0.055)	/	2.79E-01	/
		Weighted mode			0.026 (-0.025, 0.077)	/	3.11E-01	/
		MR PRESSO			0.027 (-0.006, 0.060)	/	1.09E-01	/
Fetal-specific birthweight	Household income	IVW	51	33	0.024 (-0.027, 0.075)	/	3.57E-01	4.61E-01
		MR Egger			-0.020 (-0.149, 0.109)	/	7.65E-01	/
		Simple mode			0.002 (-0.125, 0.129)	/	9.78E-01	/
		Weighted median			-0.011 (-0.064, 0.042)	/	6.84E-01	/
		Weighted mode			-0.044 (-0.128, 0.040)	/	3.11E-01	/
		MR PRESSO			0.024 (-0.017, 0.065)	/	2.67E-01	/
Fetal-specific birthweight	Occupational attainment	IVW	44	32	0.136 (0.009, 0.263)	/	3.51E-02	9.79E-02
		MR Egger			0.201 (-0.103, 0.505)	/	2.02E-01	/
		Simple mode			0.229 (-0.075, 0.533)	/	1.49E-01	/
		Weighted median			0.164 (0.021, 0.307)	/	2.44E-02	/
		Weighted mode			0.180 (-0.053, 0.413)	/	1.37E-01	/
		MR PRESSO			0.179 (0.071, 0.287)	/	2.16E-03	/
		IVW	51	33	-0.013 (-0.040, 0.014)	/	3.57E-01	4.61E-01

Fetal-specific birthweight	Townsend deprivation index	MR Egger			-0.015 (-0.086, 0.056)	/	6.71E-01	/
		Simple mode			0.003 (-0.081, 0.087)	/	9.47E-01	/
		Weighted median			-0.010 (-0.047, 0.027)	/	6.15E-01	/
		Weighted mode			0.006 (-0.063, 0.075)	/	8.66E-01	/
		MR PRESSO			-0.013 (-0.040, 0.014)	/	3.62E-01	/
Fetal-specific birthweight	Positive affect	IVW	38	33	-0.004 (-0.041, 0.033)	1.00 (0.96, 1.03)	8.34E-01	8.84E-01
		MR Egger			-0.029 (-0.147, 0.089)	0.97 (0.86, 1.09)	6.31E-01	/
		Simple mode			0.013 (-0.067, 0.093)	1.01 (0.94, 1.10)	7.60E-01	/
		Weighted median			-0.014 (-0.043, 0.015)	0.99 (0.96, 1.02)	3.51E-01	/
		Weighted mode			-0.029 (-0.078, 0.020)	0.97 (0.92, 1.02)	2.39E-01	/
		MR PRESSO			-0.019 (-0.046, 0.008)	0.98 (0.96, 1.01)	1.67E-01	/
Fetal-specific birthweight	Life satisfaction	IVW	38	33	-0.010 (-0.049, 0.029)	0.99 (0.95, 1.03)	6.24E-01	6.89E-01
		MR Egger			-0.053 (-0.175, 0.069)	0.95 (0.84, 1.07)	3.97E-01	/
		Simple mode			0.007 (-0.073, 0.087)	1.01 (0.93, 1.09)	8.71E-01	/
		Weighted median			-0.014 (-0.047, 0.019)	0.99 (0.95, 1.02)	3.97E-01	/
		Weighted mode			-0.020 (-0.063, 0.023)	0.98 (0.94, 1.02)	3.61E-01	/
		MR PRESSO			-0.003 (-0.034, 0.028)	1.00 (0.97, 1.03)	8.36E-01	/
Fetal-specific birthweight	Neuroticism	IVW	38	33	-0.004 (-0.053, 0.045)	1.00 (0.95, 1.05)	8.85E-01	9.07E-01
		MR Egger			-0.030 (-0.179, 0.119)	0.97 (0.84, 1.13)	6.94E-01	/
		Simple mode			0.037 (-0.067, 0.141)	1.04 (0.94, 1.15)	4.90E-01	/
		Weighted median			-0.016 (-0.055, 0.023)	0.98 (0.95, 1.02)	4.24E-01	/
		Weighted mode			-0.036 (-0.095, 0.023)	0.96 (0.91, 1.02)	2.48E-01	/
		MR PRESSO			-0.021 (-0.054, 0.012)	0.98 (0.95, 1.01)	2.28E-01	/
		IVW	38	33	-0.002 (-0.033, 0.029)	1.00 (0.97, 1.03)	8.90E-01	9.07E-01

Fetal-specific birthweight	Depressive symptoms	MR Egger			-0.020 (-0.116, 0.076)	0.98 (0.89, 1.08)	6.87E-01	/
		Simple mode			0.026 (-0.043, 0.095)	1.03 (0.96, 1.10)	4.76E-01	/
		Weighted median			-0.009 (-0.034, 0.016)	0.99 (0.97, 1.02)	4.84E-01	/
		Weighted mode			-0.022 (-0.059, 0.015)	0.98 (0.94, 1.02)	2.52E-01	/
		MR PRESSO			-0.005 (-0.029, 0.019)	1.00 (0.97, 1.02)	6.82E-01	/
Fetal-specific birthweight	Cigarettes smoked per day	IVW	47	32	0.049 (-0.033, 0.131)	/	2.43E-01	3.48E-01
		MR Egger			0.070 (-0.138, 0.278)	/	5.11E-01	/
		Simple mode			-0.054 (-0.283, 0.175)	/	6.51E-01	/
		Weighted median			0.056 (-0.056, 0.168)	/	3.28E-01	/
		Weighted mode			0.020 (-0.141, 0.181)	/	8.08E-01	/
		MR PRESSO			0.038 (-0.042, 0.118)	/	3.67E-01	/
Fetal-specific birthweight	Alcoholic drinks per week	IVW	46	33	0.033 (0.002, 0.064)	/	4.18E-02	1.05E-01
		MR Egger			0.019 (-0.059, 0.097)	/	6.46E-01	/
		Simple mode			0.033 (-0.047, 0.113)	/	4.17E-01	/
		Weighted median			0.025 (-0.014, 0.064)	/	2.17E-01	/
		Weighted mode			0.027 (-0.026, 0.080)	/	3.21E-01	/
		MR PRESSO			0.026 (-0.003, 0.055)	/	9.50E-02	/
Fetal-specific birthweight	Coffee consumption	IVW	50	33	0.048 (0.021, 0.075)	/	9.04E-04	6.13E-03
		MR Egger			0.056 (-0.030, 0.142)	/	2.13E-01	/
		Simple mode			0.048 (-0.015, 0.111)	/	1.43E-01	/
		Weighted median			0.037 (0.006, 0.068)	/	2.03E-02	/
		Weighted mode			0.030 (-0.021, 0.081)	/	2.53E-01	/
		MR PRESSO			0.048 (0.023, 0.073)	/	6.23E-04	/
		IVW	51	33	-0.007 (-0.017, 0.003)	0.99 (0.98, 1.00)	1.52E-01	2.62E-01

Fetal-specific birthweight	Long sleep (≥9h per night)	MR Egger			0.013 (-0.009, 0.035)	1.01 (0.99, 1.04)	2.65E-01	/
		Simple mode			-0.002 (-0.029, 0.025)	1.00 (0.97, 1.03)	9.12E-01	/
		Weighted median			-0.003 (-0.017, 0.011)	1.00 (0.98, 1.01)	6.44E-01	/
		Weighted mode			0.002 (-0.022, 0.026)	1.00 (0.98, 1.03)	8.44E-01	/
		MR PRESSO			-0.007 (-0.017, 0.003)	0.99 (0.98, 1.00)	1.58E-01	/
Fetal-specific birthweight	Short sleep (<7h per night)	IVW	51	33	0.005 (-0.009, 0.019)	1.01 (0.99, 1.02)	4.86E-01	5.72E-01
		MR Egger			-0.015 (-0.048, 0.018)	0.99 (0.95, 1.02)	3.79E-01	/
		Simple mode			-0.001 (-0.036, 0.034)	1.00 (0.96, 1.03)	9.60E-01	/
		Weighted median			-0.002 (-0.020, 0.016)	1.00 (0.98, 1.02)	8.55E-01	/
		Weighted mode			-0.003 (-0.030, 0.024)	1.00 (0.97, 1.02)	8.24E-01	/
		MR PRESSO			0.005 (-0.009, 0.019)	1.01 (0.99, 1.02)	4.89E-01	/
Fetal-specific birthweight	Chronotype	IVW	51	33	0.013 (-0.022, 0.048)	/	4.73E-01	5.70E-01
		MR Egger			-0.004 (-0.094, 0.086)	/	9.31E-01	/
		Simple mode			0.019 (-0.059, 0.097)	/	6.35E-01	/
		Weighted median			0.003 (-0.038, 0.044)	/	8.82E-01	/
		Weighted mode			0.019 (-0.034, 0.072)	/	4.81E-01	/
		MR PRESSO			0.005 (-0.028, 0.038)	/	7.86E-01	/
Fetal-specific birthweight	MPA (device-measured)	IVW	51	33	-0.730 (-1.163, -0.297)	/	9.25E-04	6.13E-03
		MR Egger			-1.059 (-2.161, 0.043)	/	6.55E-02	/
		Simple mode			-1.198 (-2.558, 0.162)	/	9.04E-02	/
		Weighted median			-0.984 (-1.641, -0.327)	/	3.35E-03	/
		Weighted mode			-1.248 (-2.261, -0.235)	/	1.95E-02	/
		MR PRESSO			-0.730 (-1.163, -0.297)		1.72E-03	/
	Childhood obesity	IVW	32	33	0.672 (0.256, 1.088)	1.96 (1.29, 2.97)	1.53E-03	8.69E-03

Fetal-specific birthweight		MR Egger			0.557 (-0.752, 1.866)	1.75 (0.47, 6.46)	4.11E-01	/
		Simple mode			0.812 (-0.452, 2.076)	2.25 (0.64, 7.97)	2.18E-01	/
		Weighted median			0.436 (-0.166, 1.038)	1.55 (0.85, 2.82)	1.56E-01	/
		Weighted mode			0.304 (-0.717, 1.325)	1.36 (0.49, 3.76)	5.63E-01	/
		MR PRESSO			0.667 (0.251, 1.083)	1.95 (1.29, 2.95)	3.71E-03	/
Fetal-specific birthweight	BMI	IVW	35	33	0.102 (0.043, 0.161)	/	7.44E-04	6.13E-03
		MR Egger			0.047 (-0.133, 0.227)	/	6.11E-01	/
		Simple mode			0.059 (-0.076, 0.194)	/	4.01E-01	/
		Weighted median			0.068 (-0.010, 0.146)	/	8.47E-02	/
		Weighted mode			0.073 (-0.023, 0.169)	/	1.49E-01	/
		MR PRESSO			0.084 (0.029, 0.139)	/	4.40E-03	/
Fetal-specific birthweight	WC	IVW	35	33	0.191 (0.109, 0.273)	/	4.28E-06	1.13E-04
		MR Egger			0.388 (0.143, 0.633)	/	3.98E-03	/
		Simple mode			0.281 (0.054, 0.508)	/	2.06E-02	/
		Weighted median			0.259 (0.163, 0.355)	/	9.31E-08	/
		Weighted mode			0.289 (0.177, 0.401)	/	1.45E-05	/
		MR PRESSO			0.191 (0.109, 0.273)	/	5.68E-05	/
Fetal-specific birthweight	WHR	IVW	35	33	0.038 (-0.064, 0.140)	/	4.72E-01	5.70E-01
		MR Egger			0.454 (0.178, 0.730)	/	2.90E-03	/
		Simple mode			-0.111 (-0.342, 0.120)	/	3.55E-01	/
		Weighted median			0.038 (-0.062, 0.138)	/	4.62E-01	/
		Weighted mode			0.016 (-0.233, 0.265)	/	8.98E-01	/
		MR-PRESSO			0.019 (-0.059, 0.097)	/	6.45E-01	/
	Height	IVW	35	33	0.397 (0.146, 0.648)	/	1.95E-03	9.10E-03

Fetal-specific birthweight		MR Egger			0.480 (-0.324, 1.284)	/	2.51E-01	/
		Simple mode			0.143 (0.023, 0.263)	/	2.40E-02	/
		Weighted median			0.111 (0.023, 0.199)	/	1.30E-02	/
		Weighted mode			0.071 (-0.013, 0.155)	/	1.05E-01	/
		MR PRESSO			0.282 (0.182, 0.382)	/	1.63E-05	/
Fetal-specific birthweight	Appendicular lean mass	IVW	51	33	0.353 (0.167, 0.539)	/	2.02E-04	2.68E-03
		MR Egger			0.165 (-0.307, 0.637)	/	4.95E-01	/
		Simple mode			0.256 (0.176, 0.336)	/	7.13E-08	/
		Weighted median			0.132 (0.073, 0.191)	/	9.25E-06	/
		Weighted mode			0.166 (0.084, 0.248)	/	2.09E-04	/
		MR PRESSO			0.274 (0.215, 0.333)	/	5.40E-10	/
Fetal-specific birthweight	Grip strength	IVW	51	33	0.076 (0.035, 0.117)	/	2.54E-04	2.69E-03
		MR Egger			0.067 (-0.037, 0.171)	/	2.13E-01	/
		Simple mode			0.022 (-0.035, 0.079)	/	4.41E-01	/
		Weighted median			0.019 (-0.014, 0.052)	/	2.47E-01	/
		Weighted mode			0.006 (-0.029, 0.041)	/	7.42E-01	/
		MR PRESSO			0.063 (0.032, 0.094)	/	2.34E-04	/
Fetal-specific birthweight	Hypertension	IVW	51	33	0.157 (-0.061, 0.375)	1.17 (0.94, 1.45)	1.58E-01	2.62E-01
		MR Egger			0.493 (-0.134, 1.120)	1.64 (0.87, 3.06)	1.30E-01	/
		Simple mode			0.035 (-0.384, 0.454)	1.04 (0.68, 1.57)	8.71E-01	/
		Weighted median			0.087 (-0.121, 0.295)	1.09 (0.89, 1.34)	4.15E-01	/
		Weighted mode			0.086 (-0.216, 0.388)	1.09 (0.81, 1.47)	5.81E-01	/
		MR PRESSO			0.067 (-0.090, 0.224)	1.07 (0.91, 1.25)	4.03E-01	/
	SBP	IVW	45	31	-0.928 (-2.735, 0.879)	/	3.14E-01	4.27E-01

Fetal-specific birthweight		MR Egger			0.382 (-5.049, 5.813)	/	8.91E-01	/
		Simple mode			-0.812 (-3.335, 1.711)	/	5.32E-01	/
		Weighted median			-0.796 (-1.782, 0.190)	/	1.14E-01	/
		Weighted mode			0.617 (-0.559, 1.793)	/	3.10E-01	/
		MR PRESSO			-1.083 (-2.057, -0.109)	/	3.80E-02	/
Fetal-specific birthweight	DBP	IVW	46	31	-0.284 (-1.219, 0.651)	/	5.52E-01	6.27E-01
		MR Egger			0.868 (-1.890, 3.626)	/	5.40E-01	/
		Simple mode			-0.650 (-1.991, 0.691)	/	3.47E-01	/
		Weighted median			-0.274 (-0.799, 0.251)	/	3.07E-01	/
		Weighted mode			0.103 (-0.677, 0.883)	/	7.96E-01	/
		MR PRESSO			-0.420 (-0.975, 0.135)	/	1.48E-01	/
Fetal-specific birthweight	Type 2 diabetes	IVW	33	32	-0.467 (-0.757, -0.177)	0.63 (0.47, 0.84)	1.64E-03	8.69E-03
		MR Egger			-0.284 (-1.178, 0.610)	0.75 (0.31, 1.84)	5.38E-01	/
		Simple mode			-0.501 (-0.871, -0.131)	0.61 (0.42, 0.88)	1.23E-02	/
		Weighted median			-0.457 (-0.665, -0.249)	0.63 (0.51, 0.78)	1.67E-05	/
		Weighted mode			-0.474 (-0.729, -0.219)	0.62 (0.48, 0.80)	9.74E-04	/
		MR PRESSO			-0.360 (-0.525, -0.195)	0.70 (0.59, 0.82)	2.13E-04	/
Fetal-specific birthweight	Fasting glucose	IVW	51	33	-0.059 (-0.100, -0.018)	/	5.96E-03	2.05E-02
		MR Egger			-0.038 (-0.152, 0.076)	/	5.18E-01	/
		Simple mode			-0.062 (-0.152, 0.028)	/	1.83E-01	/
		Weighted median			-0.047 (-0.090, -0.004)	/	3.65E-02	/
		Weighted mode			-0.049 (-0.108, 0.010)	/	1.15E-01	/
		MR PRESSO			-0.073 (-0.106, -0.040)	/	8.89E-05	/
	2-h glucose	IVW	51	33	-0.113 (-0.268, 0.042)	/	1.52E-01	2.62E-01

Fetal-specific birthweight		MR Egger			0.043 (-0.359, 0.445)	/	8.34E-01	/
		Simple mode			-0.203 (-0.675, 0.269)	/	4.03E-01	/
		Weighted median			-0.129 (-0.301, 0.043)	/	1.45E-01	/
		Weighted mode			-0.104 (-0.512, 0.304)	/	6.19E-01	/
		MR PRESSO			-0.148 (-0.291, -0.005)	/	4.87E-02	/
Fetal-specific birthweight	HbA1c	IVW	51	33	-0.051 (-0.076, -0.026)	/	7.35E-05	1.30E-03
		MR Egger			-0.062 (-0.129, 0.005)	/	7.47E-02	/
		Simple mode			-0.041 (-0.100, 0.018)	/	1.79E-01	/
		Weighted median			-0.029 (-0.054, -0.004)	/	2.64E-02	/
		Weighted mode			-0.020 (-0.057, 0.017)	/	2.93E-01	/
		MR PRESSO			-0.049 (-0.073, -0.025)	/	1.15E-04	/
Fetal-specific birthweight	Fasting insulin	IVW	51	33	-0.117 (-0.164, -0.070)	/	7.65E-07	4.05E-05
		MR Egger			-0.129 (-0.256, -0.002)	/	5.34E-02	/
		Simple mode			-0.052 (-0.162, 0.058)	/	3.55E-01	/
		Weighted median			-0.112 (-0.155, -0.069)	/	4.47E-07	/
		Weighted mode			-0.134 (-0.205, -0.063)	/	4.60E-04	/
		MR PRESSO			-0.094 (-0.129, -0.059)	/	4.66E-06	/
Fetal-specific birthweight	Total cholesterol	IVW	34	33	-0.197 (-0.324, -0.070)	/	2.42E-03	9.87E-03
		MR Egger			0.097 (-0.273, 0.467)	/	6.13E-01	/
		Simple mode			-0.123 (-0.272, 0.026)	/	1.13E-01	/
		Weighted median			-0.112 (-0.208, -0.016)	/	2.12E-02	/
		Weighted mode			-0.112 (-0.220, -0.004)	/	5.09E-02	/
		MR PRESSO			-0.133 (-0.211, -0.055)	/	2.24E-03	/
	HDL-C	IVW	34	33	-0.083 (-0.208, 0.042)	/	1.98E-01	3.09E-01

Fetal-specific birthweight		MR Egger			-0.385 (-0.750, -0.020)	/	4.69E-02	/
		Simple mode			0.018 (-0.196, 0.232)	/	8.71E-01	/
		Weighted median			-0.067 (-0.181, 0.047)	/	2.47E-01	/
		Weighted mode			-0.012 (-0.318, 0.294)	/	9.41E-01	/
		MR PRESSO			0.011 (-0.063, 0.085)	/	7.74E-01	/
Fetal-specific birthweight	LDL-C	IVW	33	33	-0.128 (-0.273, 0.017)	/	8.23E-02	1.90E-01
		MR Egger			0.269 (-0.143, 0.681)	/	2.11E-01	/
		Simple mode			0.018 (-0.170, 0.206)	/	8.49E-01	/
		Weighted median			0.030 (-0.076, 0.136)	/	5.78E-01	/
		Weighted mode			0.047 (-0.059, 0.153)	/	3.92E-01	/
		MR PRESSO			-0.088 (-0.182, 0.006)	/	7.66E-02	/
Fetal-specific birthweight	Triglycerides	IVW	33	33	-0.058 (-0.164, 0.048)	/	2.84E-01	3.96E-01
		MR Egger			0.300 (0.004, 0.596)	/	5.58E-02	/
		Simple mode			-0.037 (-0.307, 0.233)	/	7.93E-01	/
		Weighted median			-0.029 (-0.139, 0.081)	/	6.05E-01	/
		Weighted mode			0.138 (-0.017, 0.293)	/	8.78E-02	/
		MR-PRESSO			-0.085 (-0.179, 0.009)	/	8.51E-02	/
Fetal-specific birthweight	ApoA-I	IVW	51	33	-0.036 (-0.189, 0.117)	/	6.44E-01	6.97E-01
		MR Egger			-0.276 (-0.668, 0.116)	/	1.74E-01	/
		Simple mode			-0.167 (-0.592, 0.258)	/	4.46E-01	/
		Weighted median			-0.140 (-0.330, 0.050)	/	1.48E-01	/
		Weighted mode			-0.184 (-0.511, 0.143)	/	2.76E-01	/
		MR PRESSO			-0.036 (-0.189, 0.117)	/	6.46E-01	/
	ApoB	IVW	51	33	-0.185 (-0.316, -0.054)	/	6.19E-03	2.05E-02

Fetal-specific birthweight		MR Egger			-0.174 (-0.523, 0.175)	/	3.34E-01	/
		Simple mode			-0.110 (-0.445, 0.225)	/	5.22E-01	/
		Weighted median			-0.148 (-0.332, 0.036)	/	1.14E-01	/
		Weighted mode			-0.132 (-0.418, 0.154)	/	3.73E-01	/
		MR PRESSO			-0.185 (-0.316, -0.054)	/	8.54E-03	/
Fetal-specific birthweight	Omega-3 fatty acids	IVW	51	33	-0.058 (-0.138, 0.022)	/	1.56E-01	2.62E-01
		MR Egger			0.043 (-0.161, 0.247)	/	6.81E-01	/
		Simple mode			0.060 (-0.122, 0.242)	/	5.20E-01	/
		Weighted median			0.007 (-0.073, 0.087)	/	8.64E-01	/
		Weighted mode			0.069 (-0.076, 0.214)	/	3.58E-01	/
		MR PRESSO			-0.039 (-0.102, 0.024)	/	2.41E-01	/
Fetal-specific birthweight	Omega-6 fatty acids	IVW	51	33	-0.064 (-0.140, 0.012)	/	9.86E-02	2.01E-01
		MR Egger			-0.115 (-0.309, 0.079)	/	2.48E-01	/
		Simple mode			0.003 (-0.175, 0.181)	/	9.73E-01	/
		Weighted median			-0.054 (-0.136, 0.028)	/	1.97E-01	/
		Weighted mode			-0.044 (-0.160, 0.072)	/	4.60E-01	/
		MR PRESSO			-0.049 (-0.116, 0.018)	/	1.57E-01	/
Fetal-specific birthweight	DHA	IVW	51	33	-0.038 (-0.101, 0.025)	/	2.33E-01	3.48E-01
		MR Egger			-0.039 (-0.198, 0.120)	/	6.34E-01	/
		Simple mode			0.024 (-0.113, 0.161)	/	7.37E-01	/
		Weighted median			0.009 (-0.065, 0.083)	/	8.21E-01	/
		Weighted mode			0.013 (-0.091, 0.117)	/	8.03E-01	/
		MR PRESSO			-0.014 (-0.067, 0.039)	/	6.00E-01	/
	Linoleic acid	IVW	51	33	-0.052 (-0.126, 0.022)	/	1.68E-01	2.70E-01

Fetal-specific birthweight		MR Egger			-0.101 (-0.291, 0.089)	/	3.01E-01	/
		Simple mode			0.018 (-0.164, 0.200)	/	8.44E-01	/
		Weighted median			-0.063 (-0.143, 0.017)	/	1.31E-01	/
		Weighted mode			-0.055 (-0.169, 0.059)	/	3.45E-01	/
		MR PRESSO			-0.038 (-0.105, 0.029)	/	2.64E-01	/
Fetal-specific birthweight	Isoleucine	IVW	51	33	-0.053 (-0.116, 0.010)	/	9.08E-02	1.99E-01
		MR Egger			0.027 (-0.130, 0.184)	/	7.37E-01	/
		Simple mode			-0.091 (-0.242, 0.060)	/	2.44E-01	/
		Weighted median			-0.057 (-0.133, 0.019)	/	1.47E-01	/
		Weighted mode			-0.079 (-0.189, 0.031)	/	1.63E-01	/
		MR PRESSO			-0.056 (-0.105, -0.007)	/	2.95E-02	/
Fetal-specific birthweight	Leucine	IVW	51	33	-0.069 (-0.130, -0.008)	/	2.66E-02	7.83E-02
		MR Egger			-0.016 (-0.171, 0.139)	/	8.44E-01	/
		Simple mode			0.026 (-0.117, 0.169)	/	7.19E-01	/
		Weighted median			0.002 (-0.072, 0.076)	/	9.52E-01	/
		Weighted mode			0.023 (-0.116, 0.162)	/	7.50E-01	/
		MR PRESSO			-0.071 (-0.124, -0.018)	/	1.07E-02	/
Fetal-specific birthweight	Valine	IVW	51	33	-0.061 (-0.134, 0.012)	/	9.39E-02	1.99E-01
		MR Egger			0.057 (-0.123, 0.237)	/	5.37E-01	/
		Simple mode			0.044 (-0.091, 0.179)	/	5.27E-01	/
		Weighted median			-0.004 (-0.080, 0.072)	/	9.28E-01	/
		Weighted mode			0.027 (-0.081, 0.135)	/	6.26E-01	/
		MR PRESSO			-0.073 (-0.130, -0.016)	/	1.68E-02	/
	Phenylalanine	IVW	51	33	-0.067 (-0.126, -0.008)	/	2.49E-02	7.76E-02

Fetal-specific birthweight		MR Egger			-0.009 (-0.158, 0.140)	/	9.08E-01	/
		Simple mode			-0.094 (-0.255, 0.067)	/	2.57E-01	/
		Weighted median			-0.069 (-0.145, 0.007)	/	7.24E-02	/
		Weighted mode			-0.084 (-0.198, 0.030)	/	1.55E-01	/
		MR PRESSO			-0.056 (-0.109, -0.003)	/	4.44E-02	/
Fetal-specific birthweight	Tyrosine	IVW	51	33	-0.066 (-0.139, 0.007)	/	7.38E-02	1.78E-01
		MR Egger			0.051 (-0.131, 0.233)	/	5.87E-01	/
		Simple mode			-0.081 (-0.244, 0.082)	/	3.31E-01	/
		Weighted median			-0.035 (-0.119, 0.049)	/	4.12E-01	/
		Weighted mode			-0.038 (-0.161, 0.085)	/	5.49E-01	/
		MR PRESSO			-0.054 (-0.121, 0.013)	/	1.19E-01	/
Fetal-specific birthweight	Alanine	IVW	51	33	-0.071 (-0.138, -0.004)	/	4.00E-02	1.05E-01
		MR Egger			0.154 (-0.005, 0.313)	/	6.28E-02	/
		Simple mode			0.032 (-0.152, 0.216)	/	7.31E-01	/
		Weighted median			0.025 (-0.053, 0.103)	/	5.32E-01	/
		Weighted mode			0.049 (-0.055, 0.153)	/	3.53E-01	/
		MR-PRESSO			-0.071 (-0.138, -0.004)	/	4.52E-02	/
Fetal-specific birthweight	Glutamine	IVW	51	33	0.059 (-0.039, 0.157)	/	2.37E-01	3.48E-01
		MR Egger			0.009 (-0.238, 0.256)	/	9.46E-01	/
		Simple mode			0.112 (-0.090, 0.314)	/	2.81E-01	/
		Weighted median			0.014 (-0.066, 0.094)	/	7.32E-01	/
		Weighted mode			-0.016 (-0.147, 0.115)	/	8.09E-01	/
		MR PRESSO			0.045 (-0.026, 0.116)	/	2.18E-01	/
	Glycine	IVW	51	33	0.109 (0.036, 0.182)	/	2.90E-03	1.10E-02

Fetal-specific birthweight		MR Egger			0.077 (-0.107, 0.261)	/	4.17E-01	/
		Simple mode			0.109 (-0.077, 0.295)	/	2.58E-01	/
		Weighted median			0.057 (-0.021, 0.135)	/	1.53E-01	/
		Weighted mode			0.021 (-0.087, 0.129)	/	7.10E-01	/
		MR PRESSO			0.121 (0.054, 0.188)	/	8.50E-04	/
Fetal-specific birthweight	CKD	IVW	50	33	-0.119 (-0.272, 0.034)	0.89 (0.76, 1.03)	1.28E-01	2.42E-01
		MR Egger			-0.517 (-0.936, -0.098)	0.60 (0.39, 0.91)	1.94E-02	/
		Simple mode			-0.014 (-0.388, 0.360)	0.99 (0.68, 1.43)	9.42E-01	/
		Weighted median			-0.122 (-0.306, 0.062)	0.89 (0.74, 1.06)	1.95E-01	/
		Weighted mode			-0.047 (-0.345, 0.251)	0.95 (0.71, 1.29)	7.60E-01	/
		MR PRESSO			-0.174 (-0.307, -0.041)	0.84 (0.74, 0.96)	1.42E-02	/
Fetal-specific birthweight	eGFR	IVW	51	33	0.000 (-0.008, 0.008)	/	9.32E-01	9.32E-01
		MR Egger			0.019 (-0.003, 0.041)	/	1.00E-01	/
		Simple mode			-0.002 (-0.020, 0.016)	/	8.50E-01	/
		Weighted median			0.005 (-0.003, 0.013)	/	2.30E-01	/
		Weighted mode			0.009 (-0.001, 0.019)	/	1.02E-01	/
		MR PRESSO			0.000 (-0.006, 0.006)	/	1.00E+00	/
Fetal-specific birthweight	UACR	IVW	50	33	-0.057 (-0.092, -0.022)	/	2.06E-03	9.10E-03
		MR Egger			-0.058 (-0.152, 0.036)	/	2.33E-01	/
		Simple mode			-0.083 (-0.187, 0.021)	/	1.25E-01	/
		Weighted median			-0.046 (-0.087, -0.005)	/	2.74E-02	/
		Weighted mode			-0.027 (-0.092, 0.038)	/	4.13E-01	/
		MR PRESSO			-0.069 (-0.102, -0.036)	/	1.60E-04	/
	CRP	IVW	46	32	-0.033 (-0.115, 0.049)	/	4.34E-01	5.48E-01

Fetal-specific birthweight		MR Egger			-0.253 (-0.488, -0.018)	/	4.03E-02	/
		Simple mode			-0.096 (-0.204, 0.012)	/	8.84E-02	/
		Weighted median			-0.106 (-0.153, -0.059)	/	1.47E-05	/
		Weighted mode			-0.117 (-0.170, -0.064)	/	9.18E-05	/
		MR PRESSO			-0.049 (-0.096, -0.002)	/	4.76E-02	/
Association between higher Maternal-specific birthweight and candidate mediator								
Maternal-specific birthweight	Education	IVW	29	32	0.027 (-0.032, 0.086)	/	3.73E-01	6.77E-01
		MR Egger			-0.123 (-0.303, 0.057)	/	1.92E-01	/
		Simple mode			0.051 (-0.023, 0.125)	/	1.93E-01	/
		Weighted median			0.020 (-0.021, 0.061)	/	3.41E-01	/
		Weighted mode			0.061 (-0.013, 0.135)	/	1.20E-01	/
		MR PRESSO			0.018 (-0.017, 0.053)	/	3.42E-01	/
Maternal-specific birthweight	Household income	IVW	29	32	-0.002 (-0.069, 0.065)	/	9.54E-01	9.90E-01
		MR Egger			-0.077 (-0.291, 0.137)	/	4.88E-01	/
		Simple mode			0.001 (-0.128, 0.130)	/	9.84E-01	/
		Weighted median			0.000 (-0.063, 0.063)	/	9.94E-01	/
		Weighted mode			0.021 (-0.081, 0.123)	/	6.89E-01	/
		MR PRESSO			-0.024 (-0.073, 0.025)	/	3.39E-01	/
Maternal-specific birthweight	Occupational attainment	IVW	27	32	-0.006 (-0.165, 0.153)	/	9.39E-01	9.90E-01
		MR Egger			-0.230 (-0.724, 0.264)	/	3.71E-01	/
		Simple mode			-0.140 (-0.393, 0.113)	/	2.89E-01	/
		Weighted median			-0.051 (-0.202, 0.100)	/	5.12E-01	/
		Weighted mode			-0.156 (-0.385, 0.073)	/	1.94E-01	/
		MR PRESSO			-0.073 (-0.200, 0.054)	/	2.72E-01	/

Maternal-specific birthweight	Townsend deprivation index	IVW	29	32	-0.014 (-0.057, 0.029)	/	5.13E-01	7.35E-01
		MR Egger			0.015 (-0.120, 0.150)	/	8.31E-01	/
		Simple mode			-0.004 (-0.077, 0.069)	/	9.20E-01	/
		Weighted median			-0.028 (-0.069, 0.013)	/	1.85E-01	/
		Weighted mode			-0.012 (-0.077, 0.053)	/	7.21E-01	/
		MR PRESSO			-0.028 (-0.061, 0.005)	/	1.09E-01	/
Maternal-specific birthweight	Positive affect	IVW	23	31	0.000 (-0.027, 0.027)	1.00 (0.97, 1.03)	9.90E-01	9.90E-01
		MR Egger			0.046 (-0.038, 0.130)	1.05 (0.96, 1.14)	2.99E-01	/
		Simple mode			0.035 (-0.014, 0.084)	1.04 (0.99, 1.09)	1.80E-01	/
		Weighted median			0.014 (-0.013, 0.041)	1.01 (0.99, 1.04)	3.32E-01	/
		Weighted mode			0.033 (-0.012, 0.078)	1.03 (0.99, 1.08)	1.62E-01	/
		MR PRESSO			0.010 (-0.010, 0.030)	1.01 (0.99, 1.03)	3.35E-01	/
Maternal-specific birthweight	Life satisfaction	IVW	23	31	-0.001 (-0.030, 0.028)	1.00 (0.97, 1.03)	9.63E-01	9.90E-01
		MR Egger			0.048 (-0.042, 0.138)	1.05 (0.96, 1.15)	3.10E-01	/
		Simple mode			0.035 (-0.022, 0.092)	1.04 (0.98, 1.10)	2.31E-01	/
		Weighted median			0.012 (-0.019, 0.043)	1.01 (0.98, 1.04)	4.74E-01	/
		Weighted mode			0.033 (-0.020, 0.086)	1.03 (0.98, 1.09)	2.22E-01	/
		MR PRESSO			0.010 (-0.012, 0.032)	1.01 (0.99, 1.03)	3.62E-01	/
Maternal-specific birthweight	Neuroticism	IVW	23	31	0.001 (-0.034, 0.036)	1.00 (0.97, 1.04)	9.76E-01	9.90E-01
		MR Egger			0.054 (-0.054, 0.162)	1.06 (0.95, 1.18)	3.35E-01	/
		Simple mode			0.043 (-0.018, 0.104)	1.04 (0.98, 1.11)	1.84E-01	/
		Weighted median			0.027 (-0.012, 0.066)	1.03 (0.99, 1.07)	1.70E-01	/
		Weighted mode			0.044 (-0.015, 0.103)	1.04 (0.99, 1.11)	1.63E-01	/
		MR PRESSO			0.013 (-0.012, 0.038)	1.01 (0.99, 1.04)	3.37E-01	/

Maternal-specific birthweight	Depressive symptoms	IVW	23	31	-0.001 (-0.023, 0.021)	1.00 (0.98, 1.02)	9.40E-01	9.90E-01
		MR Egger			0.034 (-0.035, 0.103)	1.03 (0.97, 1.11)	3.48E-01	/
		Simple mode			0.028 (-0.013, 0.069)	1.03 (0.99, 1.07)	1.96E-01	/
		Weighted median			0.012 (-0.012, 0.036)	1.01 (0.99, 1.04)	3.20E-01	/
		Weighted mode			0.028 (-0.011, 0.067)	1.03 (0.99, 1.07)	1.81E-01	/
		MR PRESSO			0.007 (-0.009, 0.023)	1.01 (0.99, 1.02)	4.13E-01	/
Maternal-specific birthweight	Cigarettes smoked per day	IVW	29	32	0.019 (-0.091, 0.129)	/	7.40E-01	8.72E-01
		MR Egger			-0.301 (-0.628, 0.026)	/	8.28E-02	/
		Simple mode			0.071 (-0.178, 0.320)	/	5.78E-01	/
		Weighted median			0.017 (-0.108, 0.142)	/	7.90E-01	/
		Weighted mode			0.035 (-0.163, 0.233)	/	7.28E-01	/
		MR PRESSO			0.049 (-0.053, 0.151)	/	3.52E-01	/
Maternal-specific birthweight	Alcoholic drinks per week	IVW	29	32	0.005 (-0.036, 0.046)	/	8.11E-01	9.34E-01
		MR Egger			0.007 (-0.126, 0.140)	/	9.17E-01	/
		Simple mode			-0.012 (-0.081, 0.057)	/	7.23E-01	/
		Weighted median			-0.018 (-0.059, 0.023)	/	3.84E-01	/
		Weighted mode			-0.018 (-0.073, 0.037)	/	5.23E-01	/
		MR PRESSO			0.004 (-0.031, 0.039)	/	8.34E-01	/
Maternal-specific birthweight	Coffee consumption	IVW	29	32	0.040 (0.009, 0.071)	/	1.60E-02	8.48E-02
		MR Egger			-0.060 (-0.156, 0.036)	/	2.27E-01	/
		Simple mode			0.041 (-0.032, 0.114)	/	2.77E-01	/
		Weighted median			0.026 (-0.009, 0.061)	/	1.48E-01	/
		Weighted mode			0.021 (-0.044, 0.086)	/	5.22E-01	/
		MR-PRESSO			0.031 (0.004, 0.058)	/	3.80E-02	/

Maternal-specific birthweight	Long sleep (≥9h per night)	IVW	29	32	0.001 (-0.011, 0.013)	1.00 (0.99, 1.01)	8.49E-01	9.57E-01
		MR Egger			0.011 (-0.026, 0.048)	1.01 (0.97, 1.05)	5.75E-01	/
		Simple mode			0.002 (-0.031, 0.035)	1.00 (0.97, 1.04)	9.25E-01	/
		Weighted median			0.005 (-0.009, 0.019)	1.01 (0.99, 1.02)	5.10E-01	/
		Weighted mode			0.006 (-0.019, 0.031)	1.01 (0.98, 1.03)	6.52E-01	/
		MR PRESSO			0.001 (-0.011, 0.013)	1.00 (0.99, 1.01)	8.50E-01	/
Maternal-specific birthweight	Short sleep (<7h per night)	IVW	29	32	0.005 (-0.013, 0.023)	1.01 (0.99, 1.02)	5.89E-01	7.81E-01
		MR Egger			0.023 (-0.034, 0.080)	1.02 (0.97, 1.08)	4.36E-01	/
		Simple mode			0.010 (-0.023, 0.043)	1.01 (0.98, 1.04)	5.61E-01	/
		Weighted median			0.009 (-0.011, 0.029)	1.01 (0.99, 1.03)	3.87E-01	/
		Weighted mode			0.011 (-0.016, 0.038)	1.01 (0.98, 1.04)	4.31E-01	/
		MR PRESSO			0.005 (-0.013, 0.023)	1.01 (0.99, 1.02)	5.93E-01	/
Maternal-specific birthweight	Chronotype	IVW	29	32	-0.011 (-0.052, 0.030)	/	6.10E-01	7.81E-01
		MR Egger			-0.126 (-0.248, -0.004)	/	5.43E-02	/
		Simple mode			-0.011 (-0.127, 0.105)	/	8.56E-01	/
		Weighted median			-0.022 (-0.069, 0.025)	/	3.55E-01	/
		Weighted mode			-0.019 (-0.146, 0.108)	/	7.75E-01	/
		MR PRESSO			0.005 (-0.034, 0.044)	/	7.97E-01	/
Maternal-specific birthweight	MPA (device-measured)	IVW	29	32	-0.178 (-0.682, 0.326)	/	4.88E-01	7.35E-01
		MR Egger			-0.890 (-2.470, 0.690)	/	2.80E-01	/
		Simple mode			-0.314 (-1.935, 1.307)	/	7.07E-01	/
		Weighted median			-0.058 (-0.768, 0.652)	/	8.72E-01	/
		Weighted mode			-0.101 (-1.453, 1.251)	/	8.85E-01	/
		MR PRESSO			-0.178 (-0.680, 0.324)	/	4.94E-01	/

Maternal-specific birthweight	Childhood obesity	IVW	22	33	0.544 (0.119, 0.969)	1.72 (1.13, 2.64)	1.22E-02	7.54E-02
		MR Egger			-0.093 (-1.675, 1.489)	0.91 (0.19, 4.43)	9.10E-01	/
		Simple mode			0.139 (-0.882, 1.160)	1.15 (0.41, 3.19)	7.92E-01	/
		Weighted median			0.270 (-0.308, 0.848)	1.31 (0.73, 2.33)	3.60E-01	/
		Weighted mode			0.075 (-0.815, 0.965)	1.08 (0.44, 2.62)	8.71E-01	/
		MR PRESSO			0.544 (0.179, 0.909)	1.72 (1.20, 2.48)	8.22E-03	/
Maternal-specific birthweight	BMI	IVW	25	33	0.053 (-0.023, 0.129)	/	1.74E-01	4.19E-01
		MR Egger			0.011 (-0.275, 0.297)	/	9.42E-01	/
		Simple mode			0.062 (-0.091, 0.215)	/	4.30E-01	/
		Weighted median			0.051 (-0.031, 0.133)	/	2.23E-01	/
		Weighted mode			0.060 (-0.069, 0.189)	/	3.71E-01	/
		MR PRESSO			0.038 (-0.021, 0.097)	/	2.27E-01	/
Maternal-specific birthweight	WC	IVW	25	33	0.063 (-0.037, 0.163)	/	2.17E-01	5.00E-01
		MR Egger			-0.066 (-0.442, 0.310)	/	7.34E-01	/
		Simple mode			-0.103 (-0.277, 0.071)	/	2.60E-01	/
		Weighted median			0.061 (-0.033, 0.155)	/	2.10E-01	/
		Weighted mode			0.031 (-0.087, 0.149)	/	6.10E-01	/
		MR PRESSO			-0.005 (-0.074, 0.064)	/	8.99E-01	/
Maternal-specific birthweight	WHR	IVW	25	33	-0.055 (-0.131, 0.021)	/	1.58E-01	3.99E-01
		MR Egger			-0.026 (-0.316, 0.264)	/	8.61E-01	/
		Simple mode			-0.071 (-0.257, 0.115)	/	4.63E-01	/
		Weighted median			0.005 (-0.083, 0.093)	/	9.11E-01	/
		Weighted mode			0.024 (-0.092, 0.140)	/	6.85E-01	/
		MR PRESSO			-0.055 (-0.131, 0.021)	/	1.71E-01	/

Maternal-specific birthweight	Height	IVW	24	33	0.294 (0.018, 0.570)	/	3.76E-02	1.42E-01
		MR Egger			-0.092 (-1.111, 0.927)	/	8.61E-01	/
		Simple mode			0.064 (-0.036, 0.164)	/	2.25E-01	/
		Weighted median			0.071 (-0.009, 0.151)	/	8.53E-02	/
		Weighted mode			0.064 (-0.007, 0.135)	/	9.29E-02	/
		MR PRESSO			0.212 (0.085, 0.339)	/	6.96E-03	/
Maternal-specific birthweight	Appendicular lean mass	IVW	29	32	0.237 (0.051, 0.423)	/	1.26E-02	7.54E-02
		MR Egger			0.014 (-0.574, 0.602)	/	9.63E-01	/
		Simple mode			0.208 (-0.039, 0.455)	/	1.10E-01	/
		Weighted median			0.112 (0.041, 0.183)	/	1.94E-03	/
		Weighted mode			0.074 (-0.022, 0.170)	/	1.38E-01	/
		MR PRESSO			0.236 (0.158, 0.314)	/	2.24E-04	/
Maternal-specific birthweight	Grip strength	IVW	29	32	0.051 (0.004, 0.098)	/	3.40E-02	1.42E-01
		MR Egger			0.068 (-0.083, 0.219)	/	3.89E-01	/
		Simple mode			0.058 (-0.016, 0.132)	/	1.41E-01	/
		Weighted median			0.054 (0.019, 0.089)	/	3.32E-03	/
		Weighted mode			0.061 (0.010, 0.112)	/	2.65E-02	/
		MR PRESSO			0.053 (0.016, 0.090)	/	8.87E-03	/
Maternal-specific birthweight	Hypertension	IVW	28	32	-0.582 (-0.992, -0.172)	0.56 (0.37, 0.84)	5.49E-03	6.85E-02
		MR Egger			0.012 (-1.287, 1.311)	1.01 (0.28, 3.71)	9.86E-01	/
		Simple mode			0.000 (-0.386, 0.386)	1.00 (0.68, 1.47)	9.98E-01	/
		Weighted median			-0.103 (-0.354, 0.148)	0.90 (0.70, 1.16)	4.21E-01	/
		Weighted mode			0.055 (-0.231, 0.341)	1.06 (0.79, 1.41)	7.06E-01	/
		MR PRESSO			-0.361 (-0.606, -0.116)	0.70 (0.55, 0.89)	8.94E-03	/

Maternal-specific birthweight	SBP	IVW	29	32	-4.632 (-7.321, -1.943)	/	7.36E-04	3.90E-02
		MR Egger			-1.273 (-9.850, 7.304)	/	7.73E-01	/
		Simple mode			-1.030 (-2.527, 0.467)	/	1.88E-01	/
		Weighted median			-1.510 (-2.486, -0.534)	/	2.45E-03	/
		Weighted mode			-1.030 (-2.255, 0.195)	/	1.10E-01	/
		MR PRESSO			-3.542 (-4.840, -2.244)	/	1.32E-04	/
Maternal-specific birthweight	DBP	IVW	29	32	-2.479 (-4.431, -0.527)	/	1.28E-02	7.54E-02
		MR Egger			-1.199 (-7.473, 5.075)	/	7.11E-01	/
		Simple mode			-0.032 (-0.745, 0.681)	/	9.31E-01	/
		Weighted median			-0.367 (-0.881, 0.147)	/	1.62E-01	/
		Weighted mode			-0.245 (-0.706, 0.216)	/	3.06E-01	/
		MR PRESSO			-1.714 (-2.665, -0.763)	/	4.71E-03	/
Maternal-specific birthweight	Type 2 diabetes	IVW	22	34	0.094 (-0.300, 0.488)	1.10 (0.74, 1.63)	6.41E-01	7.90E-01
		MR Egger			0.721 (-0.610, 2.052)	2.06 (0.54, 7.78)	3.01E-01	/
		Simple mode			-0.100 (-0.472, 0.272)	0.90 (0.62, 1.31)	6.03E-01	/
		Weighted median			-0.111 (-0.332, 0.110)	0.89 (0.72, 1.12)	3.25E-01	/
		Weighted mode			-0.119 (-0.487, 0.249)	0.89 (0.61, 1.28)	5.35E-01	/
		MR PRESSO			0.020 (-0.131, 0.171)	1.02 (0.88, 1.19)	8.01E-01	/
Maternal-specific birthweight	Fasting glucose	IVW	29	32	0.235 (0.014, 0.456)	/	3.71E-02	1.42E-01
		MR Egger			0.559 (-0.162, 1.280)	/	1.40E-01	/
		Simple mode			0.019 (-0.044, 0.082)	/	5.62E-01	/
		Weighted median			0.012 (-0.031, 0.055)	/	5.86E-01	/
		Weighted mode			-0.003 (-0.054, 0.048)	/	9.18E-01	/
		MR PRESSO			0.099 (0.038, 0.160)	/	5.40E-03	/

Maternal-specific birthweight	2-h glucose	IVW	29	32	0.102 (-0.165, 0.369)	/	4.50E-01	7.23E-01
		MR Egger			-0.202 (-1.086, 0.682)	/	6.57E-01	/
		Simple mode			0.153 (-0.184, 0.490)	/	3.83E-01	/
		Weighted median			0.070 (-0.102, 0.242)	/	4.24E-01	/
		Weighted mode			-0.007 (-0.262, 0.248)	/	9.57E-01	/
		MR PRESSO			0.032 (-0.093, 0.157)	/	6.19E-01	/
Maternal-specific birthweight	HbA1c	IVW	29	32	0.072 (-0.010, 0.154)	/	8.46E-02	2.49E-01
		MR Egger			0.088 (-0.182, 0.358)	/	5.31E-01	/
		Simple mode			-0.037 (-0.088, 0.014)	/	1.72E-01	/
		Weighted median			-0.024 (-0.057, 0.009)	/	1.58E-01	/
		Weighted mode			-0.034 (-0.079, 0.011)	/	1.52E-01	/
		MR PRESSO			0.011 (-0.020, 0.042)	/	5.13E-01	/
Maternal-specific birthweight	Fasting insulin	IVW	29	32	-0.061 (-0.104, -0.018)	/	6.46E-03	6.85E-02
		MR Egger			-0.121 (-0.264, 0.022)	/	1.11E-01	/
		Simple mode			-0.091 (-0.207, 0.025)	/	1.34E-01	/
		Weighted median			-0.068 (-0.113, -0.023)	/	2.98E-03	/
		Weighted mode			-0.103 (-0.199, -0.007)	/	4.36E-02	/
		MR PRESSO			-0.054 (-0.091, -0.017)	/	9.15E-03	/
Maternal-specific birthweight	Total cholesterol	IVW	25	33	0.079 (-0.080, 0.238)	/	3.32E-01	6.28E-01
		MR Egger			-0.036 (-0.636, 0.564)	/	9.06E-01	/
		Simple mode			-0.098 (-0.286, 0.090)	/	3.16E-01	/
		Weighted median			-0.016 (-0.130, 0.098)	/	7.88E-01	/
		Weighted mode			-0.002 (-0.135, 0.131)	/	9.77E-01	/
		MR PRESSO			0.027 (-0.057, 0.111)	/	5.41E-01	/

Maternal-specific birthweight	HDL-C	IVW	25	33	0.133 (-0.002, 0.268)	/	5.26E-02	1.64E-01
		MR Egger			0.289 (-0.215, 0.793)	/	2.71E-01	/
		Simple mode			0.086 (-0.132, 0.304)	/	4.45E-01	/
		Weighted median			0.072 (-0.040, 0.184)	/	2.05E-01	/
		Weighted mode			0.096 (-0.039, 0.231)	/	1.79E-01	/
		MR PRESSO			0.070 (-0.022, 0.162)	/	1.53E-01	/
Maternal-specific birthweight	LDL-C	IVW	25	33	0.056 (-0.081, 0.193)	/	4.26E-01	7.06E-01
		MR Egger			-0.115 (-0.632, 0.402)	/	6.67E-01	/
		Simple mode			-0.125 (-0.296, 0.046)	/	1.66E-01	/
		Weighted median			-0.019 (-0.123, 0.085)	/	7.15E-01	/
		Weighted mode			-0.043 (-0.161, 0.075)	/	4.85E-01	/
		MR PRESSO			-0.017 (-0.101, 0.067)	/	6.98E-01	/
Maternal-specific birthweight	Triglycerides	IVW	25	33	-0.117 (-0.201, -0.033)	/	6.45E-03	6.85E-02
		MR Egger			-0.191 (-0.507, 0.125)	/	2.48E-01	/
		Simple mode			-0.176 (-0.399, 0.047)	/	1.37E-01	/
		Weighted median			-0.151 (-0.253, -0.049)	/	3.65E-03	/
		Weighted mode			-0.196 (-0.398, 0.006)	/	6.74E-02	/
		MR PRESSO			-0.117 (-0.201, -0.033)	/	1.18E-02	/
Maternal-specific birthweight	ApoA-I	IVW	29	32	0.253 (0.059, 0.447)	/	1.05E-02	7.54E-02
		MR Egger			0.492 (-0.129, 1.113)	/	1.32E-01	/
		Simple mode			0.153 (-0.312, 0.618)	/	5.24E-01	/
		Weighted median			0.273 (0.055, 0.491)	/	1.37E-02	/
		Weighted mode			0.106 (-0.300, 0.512)	/	6.12E-01	/
		MR PRESSO			0.254 (0.099, 0.409)	/	3.33E-03	/

Maternal-specific birthweight	ApoB	IVW	29	32	-0.077 (-0.220, 0.066)	/	2.88E-01	5.65E-01
		MR Egger			0.074 (-0.379, 0.527)	/	7.53E-01	/
		Simple mode			-0.049 (-0.457, 0.359)	/	8.16E-01	/
		Weighted median			0.006 (-0.194, 0.206)	/	9.54E-01	/
		Weighted mode			0.028 (-0.258, 0.314)	/	8.51E-01	/
		MR PRESSO			-0.077 (-0.197, 0.043)	/	2.14E-01	/
Maternal-specific birthweight	Omega-3 fatty acids	IVW	29	32	-0.040 (-0.114, 0.034)	/	2.86E-01	5.65E-01
		MR Egger			0.021 (-0.216, 0.258)	/	8.63E-01	/
		Simple mode			-0.058 (-0.229, 0.113)	/	5.10E-01	/
		Weighted median			-0.053 (-0.141, 0.035)	/	2.39E-01	/
		Weighted mode			-0.047 (-0.176, 0.082)	/	4.89E-01	/
		MR PRESSO			-0.063 (-0.134, 0.008)	/	8.95E-02	/
Maternal-specific birthweight	Omega-6 fatty acids	IVW	29	32	0.041 (-0.079, 0.161)	/	4.97E-01	7.35E-01
		MR Egger			0.279 (-0.091, 0.649)	/	1.51E-01	/
		Simple mode			0.007 (-0.160, 0.174)	/	9.33E-01	/
		Weighted median			0.031 (-0.061, 0.123)	/	5.17E-01	/
		Weighted mode			0.026 (-0.096, 0.148)	/	6.84E-01	/
		MR PRESSO			-0.014 (-0.096, 0.068)	/	7.41E-01	/
Maternal-specific birthweight	DHA	IVW	29	32	-0.022 (-0.100, 0.056)	/	5.90E-01	7.81E-01
		MR Egger			0.016 (-0.235, 0.267)	/	9.04E-01	/
		Simple mode			0.054 (-0.134, 0.242)	/	5.78E-01	/
		Weighted median			0.019 (-0.067, 0.105)	/	6.62E-01	/
		Weighted mode			0.052 (-0.113, 0.217)	/	5.42E-01	/
		MR PRESSO			-0.022 (-0.100, 0.056)	/	5.94E-01	/

Maternal-specific birthweight	Linoleic acid	IVW	29	32	0.061 (-0.049, 0.171)	/	2.79E-01	5.65E-01
		MR Egger			0.298 (-0.041, 0.637)	/	9.74E-02	/
		Simple mode			0.000 (-0.151, 0.151)	/	9.97E-01	/
		Weighted median			0.041 (-0.051, 0.133)	/	3.88E-01	/
		Weighted mode			0.033 (-0.089, 0.155)	/	6.00E-01	/
		MR PRESSO			0.015 (-0.065, 0.095)	/	7.17E-01	/
Maternal-specific birthweight	Isoleucine	IVW	29	32	-0.066 (-0.133, 0.001)	/	4.94E-02	1.64E-01
		MR Egger			-0.076 (-0.288, 0.136)	/	4.86E-01	/
		Simple mode			-0.076 (-0.233, 0.081)	/	3.55E-01	/
		Weighted median			-0.102 (-0.188, -0.016)	/	2.13E-02	/
		Weighted mode			-0.091 (-0.222, 0.040)	/	1.89E-01	/
		MR PRESSO			-0.066 (-0.133, 0.001)	/	5.94E-02	/
Maternal-specific birthweight	Leucine	IVW	29	32	-0.072 (-0.158, 0.014)	/	1.05E-01	2.93E-01
		MR Egger			-0.181 (-0.455, 0.093)	/	2.08E-01	/
		Simple mode			-0.098 (-0.249, 0.053)	/	2.14E-01	/
		Weighted median			-0.101 (-0.183, -0.019)	/	1.65E-02	/
		Weighted mode			-0.151 (-0.284, -0.018)	/	3.45E-02	/
		MR PRESSO			-0.061 (-0.120, -0.002)	/	5.31E-02	/
Maternal-specific birthweight	Valine	IVW	29	32	-0.073 (-0.144, -0.002)	/	4.33E-02	1.53E-01
		MR Egger			-0.094 (-0.319, 0.131)	/	4.19E-01	/
		Simple mode			-0.126 (-0.295, 0.043)	/	1.54E-01	/
		Weighted median			-0.094 (-0.178, -0.010)	/	2.93E-02	/
		Weighted mode			-0.121 (-0.260, 0.018)	/	1.02E-01	/
		MR PRESSO			-0.047 (-0.110, 0.016)	/	1.49E-01	/

Maternal-specific birthweight	Phenylalanine	IVW	29	32	-0.032 (-0.126, 0.062)	/	5.07E-01	7.35E-01
		MR Egger			-0.180 (-0.476, 0.116)	/	2.44E-01	/
		Simple mode			0.062 (-0.148, 0.272)	/	5.69E-01	/
		Weighted median			0.019 (-0.075, 0.113)	/	6.97E-01	/
		Weighted mode			0.049 (-0.167, 0.265)	/	6.55E-01	/
		MR PRESSO			-0.031 (-0.115, 0.053)	/	4.74E-01	/
Maternal-specific birthweight	Tyrosine	IVW	29	32	-0.022 (-0.130, 0.086)	/	6.84E-01	8.24E-01
		MR Egger			-0.034 (-0.377, 0.309)	/	8.47E-01	/
		Simple mode			-0.186 (-0.372, 0.000)	/	6.15E-02	/
		Weighted median			-0.121 (-0.217, -0.025)	/	1.29E-02	/
		Weighted mode			-0.198 (-0.376, -0.020)	/	3.82E-02	/
		MR PRESSO			-0.067 (-0.147, 0.013)	/	1.13E-01	/
Maternal-specific birthweight	Alanine	IVW	29	32	-0.040 (-0.130, 0.050)	/	3.83E-01	6.77E-01
		MR Egger			-0.013 (-0.299, 0.273)	/	9.31E-01	/
		Simple mode			-0.129 (-0.354, 0.096)	/	2.72E-01	/
		Weighted median			-0.086 (-0.180, 0.008)	/	7.55E-02	/
		Weighted mode			-0.148 (-0.338, 0.042)	/	1.41E-01	/
		MR PRESSO			-0.083 (-0.159, -0.007)	/	4.52E-02	/
Maternal-specific birthweight	Glutamine	IVW	29	32	0.064 (-0.087, 0.215)	/	4.09E-01	6.99E-01
		MR Egger			0.134 (-0.348, 0.616)	/	5.90E-01	/
		Simple mode			0.028 (-0.190, 0.246)	/	8.01E-01	/
		Weighted median			0.019 (-0.085, 0.123)	/	7.18E-01	/
		Weighted mode			0.070 (-0.136, 0.276)	/	5.12E-01	/
		MR PRESSO			0.068 (-0.016, 0.152)	/	1.28E-01	/

Maternal-specific birthweight	Glycine	IVW	29	32	0.026 (-0.076, 0.128)	/	6.19E-01	7.81E-01
		MR Egger			0.242 (-0.074, 0.558)	/	1.44E-01	/
		Simple mode			-0.017 (-0.168, 0.134)	/	8.25E-01	/
		Weighted median			0.017 (-0.071, 0.105)	/	7.02E-01	/
		Weighted mode			0.010 (-0.117, 0.137)	/	8.82E-01	/
		MR PRESSO			0.046 (-0.028, 0.120)	/	2.41E-01	/
Maternal-specific birthweight	CKD	IVW	29	32	-0.127 (-0.360, 0.106)	0.88 (0.70, 1.11)	2.83E-01	5.65E-01
		MR Egger			-0.084 (-0.841, 0.673)	0.92 (0.43, 1.96)	8.30E-01	/
		Simple mode			0.091 (-0.289, 0.471)	1.10 (0.75, 1.60)	6.43E-01	/
		Weighted median			0.000 (-0.212, 0.212)	1.00 (0.81, 1.24)	9.98E-01	/
		Weighted mode			0.091 (-0.283, 0.465)	1.10 (0.75, 1.59)	6.37E-01	/
		MR PRESSO			-0.014 (-0.165, 0.137)	0.99 (0.85, 1.15)	8.58E-01	/
Maternal-specific birthweight	eGFR	IVW	29	32	0.015 (0.001, 0.029)	/	2.47E-02	1.19E-01
		MR Egger			0.000 (-0.041, 0.041)	/	9.95E-01	/
		Simple mode			-0.002 (-0.016, 0.012)	/	7.55E-01	/
		Weighted median			0.007 (-0.001, 0.015)	/	1.35E-01	/
		Weighted mode			0.000 (-0.012, 0.012)	/	9.67E-01	/
		MR PRESSO			0.006 (-0.002, 0.014)	/	1.28E-01	/
Maternal-specific birthweight	UACR	IVW	29	32	-0.036 (-0.083, 0.011)	/	1.40E-01	3.71E-01
		MR Egger			-0.115 (-0.266, 0.036)	/	1.49E-01	/
		Simple mode			-0.057 (-0.149, 0.035)	/	2.34E-01	/
		Weighted median			-0.045 (-0.090, 0.000)	/	5.33E-02	/
		Weighted mode			-0.048 (-0.113, 0.017)	/	1.58E-01	/
		MR PRESSO			-0.039 (-0.082, 0.004)	/	8.65E-02	/

Maternal-specific birthweight	CRP	IVW	26	32	-0.146 (-0.248, -0.044)	/	5.05E-03	6.85E-02
		MR Egger			-0.525 (-0.809, -0.241)	/	1.36E-03	/
		Simple mode			-0.057 (-0.147, 0.033)	/	2.28E-01	/
		Weighted median			-0.034 (-0.089, 0.021)	/	2.23E-01	/
		Weighted mode			-0.032 (-0.097, 0.033)	/	3.32E-01	/
		MR PRESSO			-0.069 (-0.116, -0.022)	/	9.40E-03	/

[a]β (95% CI) and OR (95% CI) represent the associations of each 1-SD increase in birthweight with candidate mediators.
Abbreviations: ApoA-I=Apolipoprotein A-I; ApoB=Apolipoprotein B; BMI=body mass index; CI=confidence interval; CKD=Chronic kidney disease; CRP=C-reactive protein; DBP=diastolic blood pressure; DHA=docosahexaenoic acid; eGFR=estimated glomerular filtration rate; FDR=false discovery rate ; HbA1c=glycated hemoglobin; HDL-C=high-density lipoprotein cholesterol; IVW=inverse variance weighted; LDL-C=low-density lipoprotein cholesterol; No=number; OR=odds ratio; SBP=systolic blood pressure; SD=standard deviation; SNP=single nucleotide polymorphism; UACR=urinary albumin-to-creatinine ratio; UVMR=univariable Mendelian randomization; WC=Waist circumference; WHR=waist-to-hip ratio.

Table S6. UVMR pleiotropy and heterogeneity test for the causal associations between birthweight and candidate mediators

Exposure	Outcome	Pleiotropy test			Heterogeneity test		
		Egger_intercept	Intercept_se	$P_{intercept}$	Q statistic	Q_df	$P_{heterogeneity}$
Association between birthweight and candidate mediator							
Birthweight	Education	0.000	0.001	7.98E-01	466	121	6.48E-42
Birthweight	Household income	0.000	0.001	9.31E-01	262	122	3.33E-12
Birthweight	Occupational attainment	0.000	0.002	9.77E-01	170	103	3.38E-05
Birthweight	Townsend deprivation index	0.000	0.001	4.69E-01	187	122	1.40E-04
Birthweight	Positive affect	0.000	0.001	9.28E-01	282	95	2.17E-20
Birthweight	Life satisfaction	0.000	0.001	6.74E-01	239	95	2.42E-14
Birthweight	Neuroticism	0.000	0.001	9.51E-01	284	95	1.04E-20
Birthweight	Depressive symptoms	0.000	0.000	9.55E-01	283	95	1.68E-20
Birthweight	Cigarettes smoked per day	0.002	0.002	3.06E-01	183	117	8.50E-05
Birthweight	Alcoholic drinks per week	0.000	0.001	8.51E-01	308	116	2.74E-19
Birthweight	Coffee consumption	-0.001	0.001	5.88E-02	415	121	6.83E-34
Birthweight	Long sleep (≥9h per night)	0.000	0.000	4.50E-01	148	122	5.75E-02
Birthweight	Short sleep (<7h per night)	0.000	0.000	7.75E-01	219	122	1.55E-07
Birthweight	Chronotype	-0.001	0.001	1.11E-01	292	122	5.14E-16
Birthweight	MPA (device-measured)	0.018	0.011	9.90E-02	171	122	2.13E-03
Birthweight	Childhood obesity	0.019	0.009	4.30E-02	94	76	7.90E-02
Birthweight	BMI	-0.002	0.002	3.44E-01	261	89	9.77E-19
Birthweight	WC	-0.004	0.002	2.36E-02	232	89	9.63E-15
Birthweight	WHR	-0.005	0.002	5.93E-03	250	89	3.22E-17
Birthweight	Height	0.000	0.004	9.27E-01	1674	88	2.12E-291

Birthweight	Appendicular lean mass	0.003	0.003	3.06E-01	5720	122	0.00E+00
Birthweight	Grip strength	0.001	0.001	2.90E-01	621	122	6.47E-68
Birthweight	Hypertension	-0.011	0.006	5.92E-02	566	118	1.07E-59
Birthweight	SBP	-0.078	0.043	6.96E-02	3117	115	0.00E+00
Birthweight	DBP	-0.029	0.027	2.74E-01	3698	116	0.00E+00
Birthweight	Type 2 diabetes	0.033	0.012	7.88E-03	2343	83	0.00E+00
Birthweight	Fasting glucose	0.000	0.002	9.24E-01	2480	121	0.00E+00
Birthweight	2-h glucose	0.005	0.004	2.11E-01	429	122	8.04E-36
Birthweight	HbA1c	0.001	0.001	4.18E-01	994	122	1.13E-136
Birthweight	Fasting insulin	-0.001	0.001	3.00E-01	453	122	1.57E-39
Birthweight	Total cholesterol	-0.005	0.003	1.64E-01	513	85	2.05E-62
Birthweight	HDL-C	0.007	0.002	8.56E-03	341	85	1.96E-32
Birthweight	LDL-C	-0.006	0.003	6.42E-02	429	84	1.02E-47
Birthweight	Triglycerides	-0.007	0.002	1.96E-03	293	84	7.13E-25
Birthweight	ApoA-I	0.005	0.003	1.19E-01	166	121	4.10E-03
Birthweight	ApoB	-0.002	0.003	4.17E-01	154	122	2.74E-02
Birthweight	Omega-3 fatty acids	-0.002	0.002	2.31E-01	340	122	1.86E-22
Birthweight	Omega-6 fatty acids	-0.001	0.002	7.64E-01	424	122	5.49E-35
Birthweight	DHA	-0.001	0.001	6.87E-01	240	122	1.16E-09
Birthweight	Linoleic acid	-0.001	0.002	7.65E-01	379	122	3.39E-28
Birthweight	Isoleucine	0.000	0.001	8.16E-01	191	122	6.27E-05
Birthweight	Leucine	0.001	0.001	6.67E-01	234	122	4.18E-09
Birthweight	Valine	0.000	0.002	8.62E-01	305	122	9.96E-18
Birthweight	Phenylalanine	0.001	0.001	5.56E-01	188	122	1.27E-04

Birthweight	Tyrosine	-0.001	0.001	4.92E-01	247	122	1.89E-10
Birthweight	Alanine	0.002	0.002	3.30E-01	336	122	6.73E-22
Birthweight	Glutamine	0.001	0.002	6.71E-01	575	122	6.40E-60
Birthweight	Glycine	0.001	0.002	4.66E-01	310	122	2.28E-18
Birthweight	CKD	0.005	0.004	1.74E-01	255	121	1.54E-11
Birthweight	eGFR	0.000	0.000	9.90E-02	698	122	1.62E-81
Birthweight	UACR	-0.001	0.001	4.99E-01	394	121	1.12E-30
Birthweight	CRP	0.000	0.002	7.96E-01	1067	106	1.48E-158
Association between fetal-specific birthweight and candidate mediator							
Fetal-specific birthweight	Education	-0.001	0.001	5.37E-01	189	50	5.95E-18
Fetal-specific birthweight	Household income	0.001	0.002	4.74E-01	129	50	5.74E-09
Fetal-specific birthweight	Occupational attainment	-0.002	0.004	6.47E-01	88	43	5.82E-05
Fetal-specific birthweight	Townsend deprivation index	0.000	0.001	9.41E-01	66	50	6.11E-02
Fetal-specific birthweight	Positive affect	0.001	0.002	6.62E-01	195	37	2.39E-23
Fetal-specific birthweight	Life satisfaction	0.001	0.002	4.66E-01	163	37	9.09E-18
Fetal-specific birthweight	Neuroticism	0.001	0.002	7.14E-01	204	37	6.63E-25
Fetal-specific birthweight	Depressive symptoms	0.000	0.001	7.05E-01	203	37	7.89E-25
Fetal-specific birthweight	Cigarettes smoked per day	-0.001	0.003	8.27E-01	67	46	2.40E-02
Fetal-specific birthweight	Alcoholic drinks per week	0.000	0.001	6.93E-01	81	45	8.95E-04
Fetal-specific birthweight	Coffee consumption	0.000	0.001	8.47E-01	106	49	4.21E-06
Fetal-specific birthweight	Long sleep (≥9h per night)	-0.001	0.000	6.90E-02	55	50	2.83E-01
Fetal-specific birthweight	Short sleep (<7h per night)	0.001	0.000	2.11E-01	70	50	3.23E-02
Fetal-specific birthweight	Chronotype	0.001	0.001	6.90E-01	109	50	2.93E-06
Fetal-specific birthweight	MPA (device-measured)	0.010	0.015	5.27E-01	50	50	4.56E-01

Fetal-specific birthweight	Childhood obesity	0.003	0.017	8.57E-01	34	31	3.35E-01
Fetal-specific birthweight	BMI	0.002	0.002	5.37E-01	50	34	3.75E-02
Fetal-specific birthweight	WC	-0.005	0.003	1.06E-01	76	34	4.62E-05
Fetal-specific birthweight	WHR	-0.012	0.004	3.68E-03	125	34	2.55E-12
Fetal-specific birthweight	Height	-0.002	0.011	8.33E-01	1158	34	2.47E-221
Fetal-specific birthweight	Appendicular lean mass	0.006	0.007	4.00E-01	3441	50	0.00E+00
Fetal-specific birthweight	Grip strength	0.000	0.001	8.57E-01	269	50	7.69E-32
Fetal-specific birthweight	Hypertension	-0.010	0.009	2.68E-01	167	50	1.54E-14
Fetal-specific birthweight	SBP	-0.037	0.073	6.18E-01	926	44	1.43E-165
Fetal-specific birthweight	DBP	-0.032	0.037	3.89E-01	779	45	1.35E-134
Fetal-specific birthweight	Type 2 diabetes	-0.005	0.012	6.73E-01	221	32	4.98E-30
Fetal-specific birthweight	Fasting glucose	-0.001	0.002	6.98E-01	184	50	3.13E-17
Fetal-specific birthweight	2-h glucose	-0.005	0.006	4.11E-01	117	50	2.58E-07
Fetal-specific birthweight	HbA1c	0.000	0.001	7.15E-01	118	50	1.75E-07
Fetal-specific birthweight	Fasting insulin	0.000	0.002	8.53E-01	180	50	1.53E-16
Fetal-specific birthweight	Total cholesterol	-0.008	0.005	1.09E-01	149	33	1.24E-16
Fetal-specific birthweight	HDL-C	0.008	0.005	9.46E-02	160	33	1.59E-18
Fetal-specific birthweight	LDL-C	-0.011	0.006	5.42E-02	174	32	1.84E-21
Fetal-specific birthweight	Triglycerides	-0.010	0.004	1.72E-02	115	32	2.85E-11
Fetal-specific birthweight	ApoA-I	0.007	0.005	2.00E-01	73	50	1.92E-02
Fetal-specific birthweight	ApoB	0.000	0.005	9.47E-01	58	50	1.97E-01
Fetal-specific birthweight	Omega-3 fatty acids	-0.003	0.003	2.94E-01	138	50	3.26E-10
Fetal-specific birthweight	Omega-6 fatty acids	0.002	0.003	5.74E-01	125	50	2.22E-08
Fetal-specific birthweight	DHA	0.000	0.002	9.88E-01	86	50	1.15E-03

Fetal-specific birthweight	Linoleic acid	0.001	0.003	5.84E-01	120	50	1.08E-07
Fetal-specific birthweight	Isoleucine	-0.002	0.002	2.79E-01	83	50	2.39E-03
Fetal-specific birthweight	Leucine	-0.002	0.002	4.67E-01	83	50	2.15E-03
Fetal-specific birthweight	Valine	-0.004	0.003	1.67E-01	113	50	9.50E-07
Fetal-specific birthweight	Phenylalanine	-0.002	0.002	4.08E-01	71	50	2.47E-02
Fetal-specific birthweight	Tyrosine	-0.003	0.003	1.77E-01	110	50	1.92E-06
Fetal-specific birthweight	Alanine	-0.007	0.002	3.97E-03	96	50	9.44E-05
Fetal-specific birthweight	Glutamine	0.001	0.003	6.69E-01	196	50	4.06E-19
Fetal-specific birthweight	Glycine	0.001	0.003	7.08E-01	120	50	1.05E-07
Fetal-specific birthweight	CKD	0.011	0.006	5.21E-02	84	49	1.24E-03
Fetal-specific birthweight	eGFR	-0.001	0.000	7.23E-02	199	50	1.23E-19
Fetal-specific birthweight	UACR	0.000	0.001	9.73E-01	109	49	2.00E-06
Fetal-specific birthweight	CRP	0.006	0.003	5.69E-02	456	45	1.97E-69
Association between maternal-specific birthweight and candidate mediator							
Maternal-specific birthweight	Education	0.005	0.003	9.70E-02	175	28	2.94E-23
Maternal-specific birthweight	Household income	0.003	0.004	4.76E-01	95	28	3.59E-09
Maternal-specific birthweight	Occupational attainment	0.007	0.008	3.58E-01	61	26	1.17E-04
Maternal-specific birthweight	Townsend deprivation index	-0.001	0.002	6.61E-01	63	28	1.59E-04
Maternal-specific birthweight	Positive affect	-0.002	0.001	2.74E-01	44	22	3.11E-03
Maternal-specific birthweight	Life satisfaction	-0.002	0.001	2.77E-01	42	22	5.66E-03
Maternal-specific birthweight	Neuroticism	-0.002	0.002	3.14E-01	46	22	1.88E-03
Maternal-specific birthweight	Depressive symptoms	-0.001	0.001	3.10E-01	46	22	2.03E-03
Maternal-specific birthweight	Cigarettes smoked per day	0.011	0.005	5.36E-02	57	28	1.07E-03
Maternal-specific birthweight	Alcoholic drinks per week	0.000	0.002	9.74E-01	67	28	4.59E-05

Maternal-specific birthweight	Coffee consumption	0.003	0.002	3.93E-02	63	28	1.86E-04
Maternal-specific birthweight	Long sleep (≥9h per night)	0.000	0.001	5.96E-01	39	28	8.70E-02
Maternal-specific birthweight	Short sleep (<7h per night)	-0.001	0.001	5.19E-01	49	28	7.87E-03
Maternal-specific birthweight	Chronotype	0.004	0.002	6.25E-02	59	28	6.19E-04
Maternal-specific birthweight	MPA (device-measured)	0.024	0.026	3.60E-01	28	28	4.68E-01
Maternal-specific birthweight	Childhood obesity	0.020	0.024	4.22E-01	15	21	7.98E-01
Maternal-specific birthweight	BMI	0.001	0.005	7.65E-01	51	24	1.04E-03
Maternal-specific birthweight	WC	0.004	0.006	4.91E-01	71	24	1.73E-06
Maternal-specific birthweight	WHR	-0.001	0.005	8.40E-01	42	24	1.14E-02
Maternal-specific birthweight	Height	0.012	0.016	4.48E-01	861	23	5.20E-167
Maternal-specific birthweight	Appendicular lean mass	0.008	0.010	4.41E-01	1423	28	1.57E-282
Maternal-specific birthweight	Grip strength	-0.001	0.002	8.24E-01	149	28	1.88E-18
Maternal-specific birthweight	Hypertension	-0.020	0.021	3.54E-01	235	27	4.68E-35
Maternal-specific birthweight	SBP	-0.114	0.140	4.25E-01	1131	28	2.08E-220
Maternal-specific birthweight	DBP	-0.043	0.103	6.77E-01	1807	28	0.00E+00
Maternal-specific birthweight	Type 2 diabetes	-0.021	0.022	3.45E-01	230	21	3.92E-37
Maternal-specific birthweight	Fasting glucose	-0.011	0.012	3.62E-01	2168	28	0.00E+00
Maternal-specific birthweight	2-h glucose	0.010	0.015	4.85E-01	143	28	1.85E-17
Maternal-specific birthweight	HbA1c	-0.001	0.004	9.07E-01	542	28	1.70E-96
Maternal-specific birthweight	Fasting insulin	0.002	0.002	3.97E-01	66	28	6.49E-05
Maternal-specific birthweight	Total cholesterol	0.004	0.010	7.00E-01	140	24	1.91E-18
Maternal-specific birthweight	HDL-C	-0.005	0.008	5.34E-01	110	24	5.49E-13
Maternal-specific birthweight	LDL-C	0.006	0.009	5.08E-01	100	24	3.14E-11
Maternal-specific birthweight	Triglycerides	0.003	0.005	6.36E-01	45	24	6.14E-03

Maternal-specific birthweight	ApoA-I	-0.008	0.010	4.34E-01	49	28	9.44E-03
Maternal-specific birthweight	ApoB	-0.005	0.007	4.98E-01	20	28	8.80E-01
Maternal-specific birthweight	Omega-3 fatty acids	-0.002	0.004	5.95E-01	49	28	9.35E-03
Maternal-specific birthweight	Omega-6 fatty acids	-0.008	0.006	1.95E-01	127	28	1.33E-14
Maternal-specific birthweight	DHA	-0.001	0.004	7.61E-01	56	28	1.22E-03
Maternal-specific birthweight	Linoleic acid	-0.008	0.006	1.61E-01	108	28	2.90E-11
Maternal-specific birthweight	Isoleucine	0.000	0.003	9.23E-01	39	28	8.63E-02
Maternal-specific birthweight	Leucine	0.004	0.005	4.19E-01	70	28	1.86E-05
Maternal-specific birthweight	Valine	0.001	0.004	8.44E-01	45	28	2.28E-02
Maternal-specific birthweight	Phenylalanine	0.005	0.005	3.11E-01	76	28	2.82E-06
Maternal-specific birthweight	Tyrosine	0.000	0.006	9.44E-01	98	28	1.03E-09
Maternal-specific birthweight	Alanine	-0.001	0.005	8.46E-01	69	28	2.56E-05
Maternal-specific birthweight	Glutamine	-0.002	0.008	7.65E-01	194	28	1.03E-26
Maternal-specific birthweight	Glycine	-0.007	0.005	1.68E-01	100	28	5.40E-10
Maternal-specific birthweight	CKD	-0.001	0.012	9.06E-01	88	28	4.06E-08
Maternal-specific birthweight	eGFR	0.000	0.001	4.73E-01	192	28	2.49E-26
Maternal-specific birthweight	UACR	0.003	0.002	2.94E-01	83	28	2.27E-07
Maternal-specific birthweight	CRP	0.013	0.005	1.09E-02	286	25	4.69E-46

Abbreviations: ApoA-I=Apolipoprotein A-I; ApoB=Apolipoprotein B; BMI=body mass index; CKD=Chronic kidney disease; CRP=C-reactive protein; DBP=diastolic blood pressure; DHA=docosahexaenoic acid; eGFR=estimated glomerular filtration rate; HbA1c=glycated hemoglobin; HDL-C=high-density lipoprotein cholesterol; LDL-C=low-density lipoprotein cholesterol; SBP=systolic blood pressure; UACR=urinary albumin-to-creatinine ratio; UVMR=univariable Mendelian randomization; WC=Waist circumference; WHR=waist-to-hip ratio.

Table S7. MVMR estimates for the causal associations of candidate mediators with MI and AF with adjustment for birthweight

Candidate mediator	Adjusted for	Outcome	Method	β (95% CI)[a]	OR (95% CI)[a]	P value
Association between candidate mediator and MI with adjustment for birthweight						
Education	Birthweight	MI	MV-IVW	-0.579 (-0.702, -0.456)	0.56 (0.50, 0.63)	3.94E-20
			MVMR-Egger	-0.318 (-0.587, -0.049)	0.73 (0.56, 0.95)	2.05E-02
Household income	Birthweight	MI	MV-IVW	-0.620 (-0.906, -0.334)	0.54 (0.40, 0.72)	2.16E-05
			MVMR-Egger	-0.611 (-0.899, -0.323)	0.54 (0.41, 0.72)	3.13E-05
Occupational attainment	Birthweight	MI	MV-IVW	-0.080 (-0.221, 0.061)	0.92 (0.80, 1.06)	2.63E-01
			MVMR-Egger	-0.155 (-0.343, 0.033)	0.86 (0.71, 1.03)	1.07E-01
Coffee consumption	Birthweight	MI	MV-IVW	0.089 (-0.321, 0.499)	1.09 (0.73, 1.65)	6.71E-01
			MVMR-Egger	0.109 (-0.299, 0.517)	1.12 (0.74, 1.68)	5.99E-01
MPA (device-measured)	Birthweight	MI	MV-IVW	-0.001 (-0.042, 0.040)	1.00 (0.96, 1.04)	9.69E-01
			MVMR-Egger	0.001 (-0.040, 0.042)	1.00 (0.96, 1.04)	9.72E-01
Childhood obesity	Birthweight	MI	MV-IVW	0.060 (-0.007, 0.127)	1.06 (0.99, 1.14)	7.94E-02
			MVMR-Egger	0.089 (0.003, 0.175)	1.09 (1.00, 1.19)	4.22E-02
BMI	Birthweight	MI	MV-IVW	0.329 (0.200, 0.458)	1.39 (1.22, 1.58)	7.26E-07
			MVMR-Egger	0.398 (0.212, 0.584)	1.49 (1.24, 1.79)	2.82E-05
WC	Birthweight	MI	MV-IVW	0.317 (0.148, 0.486)	1.37 (1.16, 1.63)	2.18E-04
			MVMR-Egger	0.332 (0.158, 0.506)	1.39 (1.17, 1.66)	2.11E-04
Height	Birthweight	MI	MV-IVW	-0.067 (-0.124, -0.010)	0.94 (0.88, 0.99)	2.09E-02
			MVMR-Egger	-0.025 (-0.143, 0.093)	0.98 (0.87, 1.10)	6.77E-01
Appendicular lean mass	Birthweight	MI	MV-IVW	-0.115 (-0.178, -0.052)	0.89 (0.84, 0.95)	3.10E-04
			MVMR-Egger	-0.131 (-0.251, -0.011)	0.88 (0.78, 0.99)	3.12E-02
Grip strength	Birthweight	MI	MV-IVW	-0.370 (-0.607, -0.133)	0.69 (0.54, 0.88)	2.24E-03

			MVMR-Egger	-0.391 (-0.640, -0.142)	0.68 (0.53, 0.87)	2.07E-03
SBP	Birthweight	MI	MV-IVW	0.031 (0.025, 0.037)	1.03 (1.03, 1.04)	6.21E-25
			MVMR-Egger	0.032 (0.026, 0.038)	1.03 (1.03, 1.04)	2.37E-24
Type 2 diabetes	Birthweight	MI	MV-IVW	0.150 (0.099, 0.201)	1.16 (1.10, 1.22)	7.49E-09
			MVMR-Egger	0.072 (-0.006, 0.150)	1.07 (0.99, 1.16)	7.21E-02
Fasting glucose	Birthweight	MI	MV-IVW	0.243 (0.041, 0.445)	1.28 (1.04, 1.56)	1.88E-02
			MVMR-Egger	0.202 (-0.049, 0.453)	1.22 (0.95, 1.57)	1.14E-01
2-h glucose	Birthweight	MI	MV-IVW	0.171 (0.036, 0.306)	1.19 (1.04, 1.36)	1.34E-02
			MVMR-Egger	0.105 (-0.071, 0.281)	1.11 (0.93, 1.32)	2.46E-01
HbA1c	Birthweight	MI	MV-IVW	0.369 (0.095, 0.643)	1.45 (1.10, 1.90)	8.46E-03
			MVMR-Egger	0.307 (-0.046, 0.660)	1.36 (0.96, 1.93)	8.70E-02
Fasting insulin	Birthweight	MI	MV-IVW	0.710 (0.343, 1.077)	2.03 (1.41, 2.94)	1.51E-04
			MVMR-Egger	0.637 (0.253, 1.021)	1.89 (1.29, 2.78)	1.16E-03
Total cholesterol	Birthweight	MI	MV-IVW	0.409 (0.317, 0.501)	1.51 (1.37, 1.65)	2.60E-18
			MVMR-Egger	0.409 (0.317, 0.501)	1.51 (1.37, 1.65)	3.29E-18
LDL-C	Birthweight	MI	MV-IVW	0.412 (0.326, 0.498)	1.51 (1.39, 1.65)	1.32E-20
			MVMR-Egger	0.451 (0.347, 0.555)	1.57 (1.41, 1.74)	3.25E-17
Triglycerides	Birthweight	MI	MV-IVW	0.294 (0.196, 0.392)	1.34 (1.22, 1.48)	4.55E-09
			MVMR-Egger	0.267 (0.147, 0.387)	1.31 (1.16, 1.47)	1.14E-05
ApoB	Birthweight	MI	MV-IVW	0.398 (0.314, 0.482)	1.49 (1.37, 1.62)	5.65E-20
			MVMR-Egger	0.398 (0.312, 0.484)	1.49 (1.37, 1.62)	9.36E-20
Omega-3 fatty acids	Birthweight	MI	MV-IVW	0.095 (0.001, 0.189)	1.10 (1.00, 1.21)	5.02E-02
			MVMR-Egger	0.089 (-0.007, 0.185)	1.09 (0.99, 1.20)	6.70E-02
Omega-6 fatty acids	Birthweight	MI	MV-IVW	0.140 (0.011, 0.269)	1.15 (1.01, 1.31)	3.39E-02

			MVMR-Egger	0.134 (0.001, 0.267)	1.14 (1.00, 1.31)	4.82E-02
Linoleic acid	Birthweight	MI	MV-IVW	0.147 (0.008, 0.286)	1.16 (1.01, 1.33)	3.95E-02
			MVMR-Egger	0.142 (0.001, 0.283)	1.15 (1.00, 1.33)	4.74E-02
Isoleucine	Birthweight	MI	MV-IVW	0.205 (-0.040, 0.450)	1.23 (0.96, 1.57)	1.01E-01
			MVMR-Egger	0.074 (-0.208, 0.356)	1.08 (0.81, 1.43)	6.07E-01
Leucine	Birthweight	MI	MV-IVW	0.175 (-0.037, 0.387)	1.19 (0.96, 1.47)	1.05E-01
			MVMR-Egger	0.149 (-0.094, 0.392)	1.16 (0.91, 1.48)	2.29E-01
Valine	Birthweight	MI	MV-IVW	0.167 (-0.011, 0.345)	1.18 (0.99, 1.41)	6.64E-02
			MVMR-Egger	0.157 (-0.025, 0.339)	1.17 (0.98, 1.40)	9.29E-02
Phenylalanine	Birthweight	MI	MV-IVW	0.029 (-0.161, 0.219)	1.03 (0.85, 1.24)	7.63E-01
			MVMR-Egger	0.008 (-0.186, 0.202)	1.01 (0.83, 1.22)	9.37E-01
Tyrosine	Birthweight	MI	MV-IVW	0.043 (-0.096, 0.182)	1.04 (0.91, 1.20)	5.41E-01
			MVMR-Egger	0.045 (-0.094, 0.184)	1.05 (0.91, 1.20)	5.31E-01
Alanine	Birthweight	MI	MV-IVW	0.127 (-0.022, 0.276)	1.14 (0.98, 1.32)	9.38E-02
			MVMR-Egger	0.034 (-0.150, 0.218)	1.03 (0.86, 1.24)	7.17E-01
Glycine	Birthweight	MI	MV-IVW	-0.047 (-0.100, 0.006)	0.95 (0.90, 1.01)	8.23E-02
			MVMR-Egger	-0.015 (-0.068, 0.038)	0.99 (0.93, 1.04)	5.69E-01
CKD	Birthweight	MI	MV-IVW	0.009 (-0.073, 0.091)	1.01 (0.93, 1.10)	8.24E-01
			MVMR-Egger	-0.020 (-0.122, 0.082)	0.98 (0.89, 1.09)	7.02E-01
UACR	Birthweight	MI	MV-IVW	0.224 (-0.084, 0.532)	1.25 (0.92, 1.70)	1.53E-01
			MVMR-Egger	-0.058 (-0.481, 0.365)	0.94 (0.62, 1.44)	7.88E-01
CRP	Birthweight	MI	MV-IVW	0.032 (-0.060, 0.124)	1.03 (0.94, 1.13)	4.95E-01
			MVMR-Egger	-0.080 (-0.219, 0.059)	0.92 (0.80, 1.06)	2.61E-01
Association between candidate mediator and AF with adjustment for birthweight						

Education	Birthweight	AF	MV-IVW	-0.200 (-0.304, -0.095)	0.82 (0.74, 0.91)	1.86E-04
			MVMR-Egger	-0.162 (-0.390, 0.067)	0.85 (0.68, 1.07)	1.66E-01
Household income	Birthweight	AF	MV-IVW	-0.104 (-0.339, 0.131)	0.90 (0.71, 1.14)	3.86E-01
			MVMR-Egger	-0.101 (-0.337, 0.135)	0.90 (0.71, 1.14)	4.03E-01
Occupational attainment	Birthweight	AF	MV-IVW	-0.068 (-0.180, 0.044)	0.93 (0.84, 1.04)	2.36E-01
			MVMR-Egger	0.076 (-0.071, 0.222)	1.08 (0.93, 1.25)	3.10E-01
Coffee consumption	Birthweight	AF	MV-IVW	0.150 (-0.156, 0.456)	1.16 (0.86, 1.58)	3.38E-01
			MVMR-Egger	0.159 (-0.147, 0.465)	1.17 (0.86, 1.59)	3.08E-01
MPA (device-measured)	Birthweight	AF	MV-IVW	0.011 (-0.022, 0.044)	1.01 (0.98, 1.04)	5.07E-01
			MVMR-Egger	0.013 (-0.020, 0.046)	1.01 (0.98, 1.05)	4.41E-01
Childhood obesity	Birthweight	AF	MV-IVW	0.073 (0.021, 0.124)	1.08 (1.02, 1.13)	5.45E-03
			MVMR-Egger	0.071 (0.005, 0.137)	1.07 (1.01, 1.15)	3.59E-02
BMI	Birthweight	AF	MV-IVW	0.338 (0.251, 0.426)	1.40 (1.29, 1.53)	3.66E-14
			MVMR-Egger	0.293 (0.168, 0.419)	1.34 (1.18, 1.52)	4.55E-06
WC	Birthweight	AF	MV-IVW	0.366 (0.247, 0.485)	1.44 (1.28, 1.62)	1.53E-09
			MVMR-Egger	0.351 (0.227, 0.475)	1.42 (1.25, 1.61)	2.87E-08
Height	Birthweight	AF	MV-IVW	0.238 (0.183, 0.293)	1.27 (1.20, 1.34)	1.64E-17
			MVMR-Egger	0.344 (0.229, 0.459)	1.41 (1.26, 1.58)	5.00E-09
Appendicular lean mass	Birthweight	AF	MV-IVW	0.235 (0.176, 0.293)	1.26 (1.19, 1.34)	5.48E-15
			MVMR-Egger	0.271 (0.157, 0.386)	1.31 (1.17, 1.47)	3.40E-06
Grip strength	Birthweight	AF	MV-IVW	0.162 (-0.064, 0.389)	1.18 (0.94, 1.48)	1.60E-01
			MVMR-Egger	0.153 (-0.083, 0.389)	1.17 (0.92, 1.48)	2.04E-01
SBP	Birthweight	AF	MV-IVW	0.020 (0.015, 0.024)	1.02 (1.02, 1.02)	4.75E-17
			MVMR-Egger	0.020 (0.015, 0.025)	1.02 (1.02, 1.03)	7.95E-17

Type 2 diabetes	Birthweight	AF	MV-IVW	0.012 (-0.021, 0.044)	1.01 (0.98, 1.04)	4.74E-01
			MVMR-Egger	0.006 (-0.046, 0.057)	1.01 (0.96, 1.06)	8.34E-01
Fasting glucose	Birthweight	AF	MV-IVW	-0.028 (-0.169, 0.113)	0.97 (0.84, 1.12)	6.95E-01
			MVMR-Egger	-0.113 (-0.287, 0.060)	0.89 (0.75, 1.06)	1.99E-01
2-h glucose	Birthweight	AF	MV-IVW	-0.047 (-0.152, 0.057)	0.95 (0.86, 1.06)	3.75E-01
			MVMR-Egger	-0.083 (-0.219, 0.053)	0.92 (0.80, 1.05)	2.32E-01
HbA1c	Birthweight	AF	MV-IVW	0.014 (-0.184, 0.211)	1.01 (0.83, 1.23)	8.90E-01
			MVMR-Egger	-0.043 (-0.294, 0.209)	0.96 (0.75, 1.23)	7.39E-01
Fasting insulin	Birthweight	AF	MV-IVW	-0.047 (-0.334, 0.240)	0.95 (0.72, 1.27)	7.47E-01
			MVMR-Egger	-0.031 (-0.331, 0.270)	0.97 (0.72, 1.31)	8.42E-01
Total cholesterol	Birthweight	AF	MV-IVW	0.024 (-0.025, 0.073)	1.02 (0.98, 1.08)	3.44E-01
			MVMR-Egger	0.024 (-0.026, 0.073)	1.02 (0.97, 1.08)	3.47E-01
LDL-C	Birthweight	AF	MV-IVW	0.024 (-0.023, 0.071)	1.02 (0.98, 1.07)	3.18E-01
			MVMR-Egger	0.024 (-0.033, 0.080)	1.02 (0.97, 1.08)	4.12E-01
Triglycerides	Birthweight	AF	MV-IVW	0.035 (-0.032, 0.102)	1.04 (0.97, 1.11)	3.04E-01
			MVMR-Egger	0.014 (-0.067, 0.095)	1.01 (0.94, 1.10)	7.39E-01
ApoB	Birthweight	AF	MV-IVW	0.012 (-0.046, 0.069)	1.01 (0.96, 1.07)	6.88E-01
			MVMR-Egger	0.009 (-0.049, 0.066)	1.01 (0.95, 1.07)	7.60E-01
Omega-3 fatty acids	Birthweight	AF	MV-IVW	0.007 (-0.048, 0.061)	1.01 (0.95, 1.06)	8.13E-01
			MVMR-Egger	-0.001 (-0.055, 0.053)	1.00 (0.95, 1.05)	9.74E-01
Omega-6 fatty acids	Birthweight	AF	MV-IVW	-0.022 (-0.094, 0.050)	0.98 (0.91, 1.05)	5.53E-01
			MVMR-Egger	-0.027 (-0.100, 0.047)	0.97 (0.90, 1.05)	4.79E-01
Linoleic acid	Birthweight	AF	MV-IVW	-0.040 (-0.116, 0.036)	0.96 (0.89, 1.04)	3.02E-01
			MVMR-Egger	-0.044 (-0.120, 0.033)	0.96 (0.89, 1.03)	2.65E-01

Isoleucine	Birthweight	AF	MV-IVW	0.014 (-0.177, 0.205)	1.01 (0.84, 1.23)	8.86E-01
			MVMR-Egger	-0.014 (-0.237, 0.209)	0.99 (0.79, 1.23)	9.05E-01
Leucine	Birthweight	AF	MV-IVW	0.027 (-0.138, 0.193)	1.03 (0.87, 1.21)	7.45E-01
			MVMR-Egger	0.017 (-0.173, 0.208)	1.02 (0.84, 1.23)	8.59E-01
Valine	Birthweight	AF	MV-IVW	0.044 (-0.092, 0.180)	1.05 (0.91, 1.20)	5.25E-01
			MVMR-Egger	0.045 (-0.094, 0.184)	1.05 (0.91, 1.20)	5.28E-01
Phenylalanine	Birthweight	AF	MV-IVW	0.052 (-0.102, 0.205)	1.05 (0.90, 1.23)	5.10E-01
			MVMR-Egger	0.031 (-0.124, 0.187)	1.03 (0.88, 1.21)	6.93E-01
Tyrosine	Birthweight	AF	MV-IVW	0.108 (0.003, 0.213)	1.11 (1.00, 1.24)	4.31E-02
			MVMR-Egger	0.109 (0.004, 0.215)	1.12 (1.00, 1.24)	4.28E-02
Alanine	Birthweight	AF	MV-IVW	-0.057 (-0.177, 0.063)	0.94 (0.84, 1.07)	3.52E-01
			MVMR-Egger	-0.072 (-0.223, 0.078)	0.93 (0.80, 1.08)	3.45E-01
Glycine	Birthweight	AF	MV-IVW	-0.024 (-0.064, 0.016)	0.98 (0.94, 1.02)	2.36E-01
			MVMR-Egger	-0.025 (-0.067, 0.017)	0.98 (0.94, 1.02)	2.43E-01
CKD	Birthweight	AF	MV-IVW	0.007 (-0.059, 0.072)	1.01 (0.94, 1.07)	8.42E-01
			MVMR-Egger	0.025 (-0.057, 0.106)	1.03 (0.94, 1.11)	5.51E-01
UACR	Birthweight	AF	MV-IVW	0.257 (0.028, 0.486)	1.29 (1.03, 1.63)	2.77E-02
			MVMR-Egger	0.171 (-0.148, 0.489)	1.19 (0.86, 1.63)	2.94E-01
CRP	Birthweight	AF	MV-IVW	0.060 (0.007, 0.114)	1.06 (1.01, 1.12)	2.76E-02
			MVMR-Egger	0.030 (-0.048, 0.109)	1.03 (0.95, 1.12)	4.48E-01
Association between candidate mediator and MI with adjustment for fetal-specific birthweight						
Occupational attainment	Fetal-specific birthweight	MI	MV-IVW	-0.140 (-0.246, -0.034)	0.87 (0.78, 0.97)	9.82E-03
			MVMR-Egger	-0.173 (-0.342, -0.004)	0.84 (0.71, 1.00)	4.44E-02
Alcoholic drinks per week	Fetal-specific birthweight	MI	MV-IVW	0.124 (-0.117, 0.365)	1.13 (0.89, 1.44)	3.13E-01

			MVMR-Egger	0.158 (-0.085, 0.401)	1.17 (0.92, 1.49)	2.04E-01
Coffee consumption	Fetal-specific birthweight	MI	MV-IVW	0.075 (-0.329, 0.479)	1.08 (0.72, 1.61)	7.16E-01
			MVMR-Egger	0.218 (-0.346, 0.782)	1.24 (0.71, 2.19)	4.49E-01
MPA (device-measured)	Fetal-specific birthweight	MI	MV-IVW	0.002 (-0.035, 0.039)	1.00 (0.97, 1.04)	9.37E-01
			MVMR-Egger	0.021 (-0.026, 0.068)	1.02 (0.97, 1.07)	3.93E-01
Childhood obesity	Fetal-specific birthweight	MI	MV-IVW	0.085 (0.028, 0.142)	1.09 (1.03, 1.15)	3.03E-03
			MVMR-Egger	0.087 (0.030, 0.144)	1.09 (1.03, 1.15)	2.98E-03
BMI	Fetal-specific birthweight	MI	MV-IVW	0.357 (0.253, 0.461)	1.43 (1.29, 1.59)	2.05E-11
			MVMR-Egger	0.296 (0.124, 0.468)	1.34 (1.13, 1.60)	8.19E-04
WC	Fetal-specific birthweight	MI	MV-IVW	0.363 (0.222, 0.504)	1.44 (1.25, 1.66)	5.25E-07
			MVMR-Egger	0.410 (0.173, 0.647)	1.51 (1.19, 1.91)	6.97E-04
Height	Fetal-specific birthweight	MI	MV-IVW	-0.097 (-0.146, -0.048)	0.91 (0.86, 0.95)	1.20E-04
			MVMR-Egger	-0.067 (-0.185, 0.051)	0.94 (0.83, 1.05)	2.68E-01
Appendicular lean mass	Fetal-specific birthweight	MI	MV-IVW	-0.154 (-0.215, -0.093)	0.86 (0.81, 0.91)	6.29E-07
			MVMR-Egger	-0.171 (-0.298, -0.044)	0.84 (0.74, 0.96)	7.98E-03
Grip strength	Fetal-specific birthweight	MI	MV-IVW	-0.316 (-0.510, -0.122)	0.73 (0.60, 0.89)	1.45E-03
			MVMR-Egger	-0.316 (-0.520, -0.112)	0.73 (0.59, 0.89)	2.35E-03
Type 2 diabetes	Fetal-specific birthweight	MI	MV-IVW	0.142 (0.089, 0.195)	1.15 (1.09, 1.22)	1.56E-07
			MVMR-Egger	0.140 (0.087, 0.193)	1.15 (1.09, 1.21)	2.98E-07
Fasting glucose	Fetal-specific birthweight	MI	MV-IVW	0.213 (0.023, 0.403)	1.24 (1.02, 1.50)	2.82E-02
			MVMR-Egger	0.196 (-0.065, 0.457)	1.22 (0.94, 1.58)	1.40E-01
HbA1c	Fetal-specific birthweight	MI	MV-IVW	0.287 (0.007, 0.567)	1.33 (1.01, 1.76)	4.46E-02
			MVMR-Egger	0.245 (-0.155, 0.645)	1.28 (0.86, 1.91)	2.29E-01
Fasting insulin	Fetal-specific birthweight	MI	MV-IVW	0.694 (0.390, 0.998)	2.00 (1.48, 2.71)	7.59E-06

			MVMR-Egger	0.310 (-0.153, 0.773)	1.36 (0.86, 2.17)	1.89E-01
Total cholesterol	Fetal-specific birthweight	MI	MV-IVW	0.422 (0.326, 0.518)	1.53 (1.39, 1.68)	3.70E-18
			MVMR-Egger	0.421 (0.325, 0.517)	1.52 (1.38, 1.68)	6.39E-18
ApoB	Fetal-specific birthweight	MI	MV-IVW	0.433 (0.353, 0.513)	1.54 (1.42, 1.67)	1.94E-26
			MVMR-Egger	0.440 (0.360, 0.520)	1.55 (1.43, 1.68)	1.00E-26
Leucine	Fetal-specific birthweight	MI	MV-IVW	0.141 (-0.049, 0.331)	1.15 (0.95, 1.39)	1.47E-01
			MVMR-Egger	0.082 (-0.136, 0.300)	1.09 (0.87, 1.35)	4.61E-01
Phenylalanine	Fetal-specific birthweight	MI	MV-IVW	0.002 (-0.163, 0.167)	1.00 (0.85, 1.18)	9.78E-01
			MVMR-Egger	-0.016 (-0.188, 0.156)	0.98 (0.83, 1.17)	8.53E-01
Alanine	Fetal-specific birthweight	MI	MV-IVW	-0.010 (-0.130, 0.110)	0.99 (0.88, 1.12)	8.76E-01
			MVMR-Egger	-0.025 (-0.188, 0.138)	0.98 (0.83, 1.15)	7.67E-01
Glycine	Fetal-specific birthweight	MI	MV-IVW	-0.048 (-0.095, -0.001)	0.95 (0.91, 1.00)	4.35E-02
			MVMR-Egger	-0.042 (-0.089, 0.005)	0.96 (0.91, 1.01)	8.43E-02
UACR	Fetal-specific birthweight	MI	MV-IVW	0.147 (-0.151, 0.445)	1.16 (0.86, 1.56)	3.33E-01
			MVMR-Egger	-0.152 (-0.626, 0.322)	0.86 (0.53, 1.38)	5.31E-01
Association between candidate mediator and AF with adjustment for fetal-specific birthweight						
Occupational attainment	Fetal-specific birthweight	AF	MV-IVW	-0.005 (-0.115, 0.105)	1.00 (0.89, 1.11)	9.31E-01
			MVMR-Egger	0.084 (-0.087, 0.255)	1.09 (0.92, 1.29)	3.36E-01
Alcoholic drinks per week	Fetal-specific birthweight	AF	MV-IVW	0.093 (-0.128, 0.314)	1.10 (0.88, 1.37)	4.10E-01
			MVMR-Egger	0.115 (-0.110, 0.340)	1.12 (0.90, 1.40)	3.17E-01
Coffee consumption	Fetal-specific birthweight	AF	MV-IVW	0.241 (-0.014, 0.496)	1.27 (0.99, 1.64)	6.32E-02
			MVMR-Egger	0.171 (-0.184, 0.526)	1.19 (0.83, 1.69)	3.47E-01
MPA (device-measured)	Fetal-specific birthweight	AF	MV-IVW	0.023 (-0.014, 0.060)	1.02 (0.99, 1.06)	2.32E-01
			MVMR-Egger	0.023 (-0.024, 0.070)	1.02 (0.98, 1.07)	3.35E-01

Childhood obesity	Fetal-specific birthweight	AF	MV-IVW	0.079 (0.024, 0.134)	1.08 (1.02, 1.14)	4.62E-03
			MVMR-Egger	0.085 (0.030, 0.140)	1.09 (1.03, 1.15)	2.20E-03
BMI	Fetal-specific birthweight	AF	MV-IVW	0.329 (0.243, 0.415)	1.39 (1.28, 1.51)	1.23E-13
			MVMR-Egger	0.310 (0.165, 0.455)	1.36 (1.18, 1.58)	2.74E-05
WC	Fetal-specific birthweight	AF	MV-IVW	0.352 (0.236, 0.468)	1.42 (1.27, 1.60)	3.12E-09
			MVMR-Egger	0.463 (0.271, 0.655)	1.59 (1.31, 1.93)	2.25E-06
Height	Fetal-specific birthweight	AF	MV-IVW	0.262 (0.211, 0.313)	1.30 (1.23, 1.37)	3.01E-24
			MVMR-Egger	0.329 (0.206, 0.452)	1.39 (1.23, 1.57)	1.47E-07
Appendicular lean mass	Fetal-specific birthweight	AF	MV-IVW	0.242 (0.183, 0.301)	1.27 (1.20, 1.35)	3.39E-16
			MVMR-Egger	0.272 (0.149, 0.395)	1.31 (1.16, 1.48)	1.60E-05
Grip strength	Fetal-specific birthweight	AF	MV-IVW	0.192 (-0.018, 0.402)	1.21 (0.98, 1.49)	7.27E-02
			MVMR-Egger	0.196 (-0.024, 0.416)	1.22 (0.98, 1.52)	7.87E-02
Type 2 diabetes	Fetal-specific birthweight	AF	MV-IVW	0.019 (-0.014, 0.052)	1.02 (0.99, 1.05)	2.61E-01
			MVMR-Egger	0.020 (-0.013, 0.053)	1.02 (0.99, 1.05)	2.49E-01
Fasting glucose	Fetal-specific birthweight	AF	MV-IVW	0.011 (-0.126, 0.148)	1.01 (0.88, 1.16)	8.76E-01
			MVMR-Egger	0.004 (-0.182, 0.190)	1.00 (0.83, 1.21)	9.70E-01
HbA1c	Fetal-specific birthweight	AF	MV-IVW	0.011 (-0.177, 0.199)	1.01 (0.84, 1.22)	9.07E-01
			MVMR-Egger	0.040 (-0.227, 0.307)	1.04 (0.80, 1.36)	7.70E-01
Fasting insulin	Fetal-specific birthweight	AF	MV-IVW	-0.059 (-0.345, 0.227)	0.94 (0.71, 1.25)	6.88E-01
			MVMR-Egger	-0.421 (-0.854, 0.012)	0.66 (0.43, 1.01)	5.70E-02
Total cholesterol	Fetal-specific birthweight	AF	MV-IVW	0.026 (-0.023, 0.075)	1.03 (0.98, 1.08)	2.99E-01
			MVMR-Egger	0.025 (-0.024, 0.074)	1.03 (0.98, 1.08)	3.18E-01
ApoB	Fetal-specific birthweight	AF	MV-IVW	0.011 (-0.044, 0.066)	1.01 (0.96, 1.07)	6.95E-01
			MVMR-Egger	0.005 (-0.050, 0.060)	1.01 (0.95, 1.06)	8.70E-01

Leucine	Fetal-specific birthweight	AF	MV-IVW	0.045 (-0.118, 0.208)	1.05 (0.89, 1.23)	5.86E-01
			MVMR-Egger	-0.024 (-0.208, 0.160)	0.98 (0.81, 1.17)	8.02E-01
Phenylalanine	Fetal-specific birthweight	AF	MV-IVW	-0.003 (-0.158, 0.152)	1.00 (0.85, 1.16)	9.73E-01
			MVMR-Egger	-0.041 (-0.202, 0.120)	0.96 (0.82, 1.13)	6.17E-01
Alanine	Fetal-specific birthweight	AF	MV-IVW	-0.016 (-0.130, 0.098)	0.98 (0.88, 1.10)	7.76E-01
			MVMR-Egger	0.014 (-0.137, 0.165)	1.01 (0.87, 1.18)	8.60E-01
Glycine	Fetal-specific birthweight	AF	MV-IVW	-0.024 (-0.061, 0.013)	0.98 (0.94, 1.01)	2.01E-01
			MVMR-Egger	-0.017 (-0.054, 0.020)	0.98 (0.95, 1.02)	3.67E-01
UACR	Fetal-specific birthweight	AF	MV-IVW	0.241 (0.021, 0.461)	1.27 (1.02, 1.59)	3.13E-02
			MVMR-Egger	0.149 (-0.206, 0.504)	1.16 (0.81, 1.66)	4.09E-01
Association between candidate mediator and MI with adjustment for maternal-specific birthweight						
Coffee consumption	Maternal-specific birthweight	MI	MV-IVW	0.224 (-0.246, 0.694)	1.25 (0.78, 2.00)	3.51E-01
			MVMR-Egger	0.160 (-0.350, 0.670)	1.17 (0.70, 1.95)	5.38E-01
Childhood obesity	Maternal-specific birthweight	MI	MV-IVW	0.110 (0.020, 0.200)	1.12 (1.02, 1.22)	1.63E-02
			MVMR-Egger	0.088 (-0.022, 0.198)	1.09 (0.98, 1.22)	1.18E-01
Height	Maternal-specific birthweight	MI	MV-IVW	-0.088 (-0.141, -0.035)	0.92 (0.87, 0.97)	1.07E-03
			MVMR-Egger	-0.021 (-0.144, 0.102)	0.98 (0.87, 1.11)	7.37E-01
Appendicular lean mass	Maternal-specific birthweight	MI	MV-IVW	-0.132 (-0.187, -0.077)	0.88 (0.83, 0.93)	2.64E-06
			MVMR-Egger	-0.130 (-0.185, -0.075)	0.88 (0.83, 0.93)	4.98E-06
Grip strength	Maternal-specific birthweight	MI	MV-IVW	-0.359 (-0.559, -0.159)	0.70 (0.57, 0.85)	4.43E-04
			MVMR-Egger	-0.356 (-0.558, -0.154)	0.70 (0.57, 0.86)	5.81E-04
Hypertension	Maternal-specific birthweight	MI	MV-IVW	0.275 (0.195, 0.355)	1.32 (1.22, 1.43)	2.95E-11
			MVMR-Egger	0.279 (0.193, 0.365)	1.32 (1.21, 1.44)	2.53E-10
SBP	Maternal-specific birthweight	MI	MV-IVW	0.030 (0.024, 0.036)	1.03 (1.02, 1.04)	7.99E-22

			MVMR-Egger	0.030 (0.024, 0.036)	1.03 (1.02, 1.04)	1.10E-21
DBP	Maternal-specific birthweight	MI	MV-IVW	0.054 (0.044, 0.064)	1.06 (1.04, 1.07)	4.91E-29
			MVMR-Egger	0.069 (0.045, 0.093)	1.07 (1.05, 1.10)	1.87E-09
Fasting glucose	Maternal-specific birthweight	MI	MV-IVW	0.477 (0.242, 0.712)	1.61 (1.27, 2.04)	7.60E-05
			MVMR-Egger	0.480 (0.137, 0.823)	1.62 (1.15, 2.28)	6.07E-03
Fasting insulin	Maternal-specific birthweight	MI	MV-IVW	0.859 (0.459, 1.259)	2.36 (1.58, 3.52)	2.59E-05
			MVMR-Egger	0.441 (-0.269, 1.151)	1.55 (0.76, 3.16)	2.22E-01
Triglycerides	Maternal-specific birthweight	MI	MV-IVW	0.317 (0.205, 0.429)	1.37 (1.23, 1.54)	2.38E-08
			MVMR-Egger	0.196 (0.043, 0.349)	1.22 (1.04, 1.42)	1.17E-02
ApoA-I	Maternal-specific birthweight	MI	MV-IVW	0.007 (-0.115, 0.129)	1.01 (0.89, 1.14)	9.13E-01
			MVMR-Egger	-0.007 (-0.158, 0.144)	0.99 (0.85, 1.15)	9.29E-01
Isoleucine	Maternal-specific birthweight	MI	MV-IVW	0.072 (-0.236, 0.380)	1.07 (0.79, 1.46)	6.45E-01
			MVMR-Egger	-0.044 (-0.426, 0.338)	0.96 (0.65, 1.40)	8.23E-01
Valine	Maternal-specific birthweight	MI	MV-IVW	0.145 (-0.076, 0.366)	1.16 (0.93, 1.44)	1.97E-01
			MVMR-Egger	0.141 (-0.080, 0.362)	1.15 (0.92, 1.44)	2.12E-01
eGFR	Maternal-specific birthweight	MI	MV-IVW	0.432 (-0.244, 1.108)	1.54 (0.78, 3.03)	2.11E-01
			MVMR-Egger	0.894 (-0.629, 2.417)	2.44 (0.53, 11.21)	2.50E-01
CRP	Maternal-specific birthweight	MI	MV-IVW	0.028 (-0.070, 0.126)	1.03 (0.93, 1.13)	5.68E-01
			MVMR-Egger	0.028 (-0.070, 0.126)	1.03 (0.93, 1.13)	5.74E-01

[a]β (95% CI) and OR (95% CI) represent the direct effect of each 1-unit increase in candidate mediator on MI and AF after adjusting for birthweight.
Abbreviations: ApoA-I=Apolipoprotein A-I; ApoB=Apolipoprotein B; AF=atrial fibrillation; BMI=body mass index; CI=confidence interval; DBP=diastolic blood pressure; eGFR=estimated glomerular filtration rate; HbA1c=glycated hemoglobin; LDL-C=low-density lipoprotein cholesterol; MV-IVW=multivariable Mendelian randomization inverse variance weighted; MI=myocardial infarction; MVMR=multivariable Mendelian randomization; MVMR Egger, multivariable Mendelian randomization Egger; OR=odds ratio; SBP=systolic blood pressure; UACR=urinary albumin-to-creatinine ratio; WC=Waist circumference; WHR=waist-to-hip ratio.

Table S8. Mediation analysis of the effect of fetal/maternal-specific birthweight on MI and AF via mediators

Mediator	Exposure	Outcome	Total effect, β (95% CI)	Effect of exposure on mediator, β1 (95% CI)	Effect of mediator on outcome, β2 (95% CI)	Mediated proportion (95% CI)
Mediator between fetal-specific birthweight and MI						
Total cholesterol	Fetal-specific birthweight	MI	-0.268 (-0.413, -0.123)	-0.197 (-0.324, -0.070)	0.422 (0.326, 0.518)	30.92% (9.76%, 52.09%)
Fasting insulin	Fetal-specific birthweight	MI	-0.268 (-0.413, -0.123)	-0.117 (-0.164, -0.070)	0.694 (0.390, 0.998)	30.33% (12.41%, 48.24%)
ApoB	Fetal-specific birthweight	MI	-0.268 (-0.413, -0.123)	-0.185 (-0.316, -0.054)	0.433 (0.353, 0.513)	29.75% (7.76%, 51.75%)
Type 2 diabetes	Fetal-specific birthweight	MI	-0.268 (-0.413, -0.123)	-0.467 (-0.757, -0.177)	0.142 (0.089, 0.195)	24.71% (6.77%, 42.65%)
Appendicular lean mass	Fetal-specific birthweight	MI	-0.268 (-0.413, -0.123)	0.353 (0.167, 0.539)	-0.154 (-0.215, -0.093)	20.22% (6.92%, 33.52%)
Height	Fetal-specific birthweight	MI	-0.268 (-0.413, -0.123)	0.397 (0.146, 0.648)	-0.097 (-0.146, -0.048)	14.37% (2.70%, 26.04%)
Grip strength	Fetal-specific birthweight	MI	-0.268 (-0.413, -0.123)	0.076 (0.035, 0.117)	-0.316 (-0.510, -0.122)	8.98% (1.65%, 16.31%)
Occupational attainment	Fetal-specific birthweight	MI	-0.268 (-0.413, -0.123)	0.136 (0.009, 0.263)	-0.140 (-0.246, -0.034)	7.09% (0.00%, 15.60%)
HbA1c	Fetal-specific birthweight	MI	-0.268 (-0.413, -0.123)	-0.051 (-0.076, -0.026)	0.287 (0.007, 0.567)	5.40% (0.00%, 11.31%)
Fasting glucose	Fetal-specific birthweight	MI	-0.268 (-0.413, -0.123)	-0.059 (-0.100, -0.018)	0.213 (0.023, 0.403)	4.64% (0.00%, 9.94%)
Glycine	Fetal-specific birthweight	MI	-0.268 (-0.413, -0.123)	0.109 (0.036, 0.182)	-0.048 (-0.095, -0.001)	1.95% (0.00%, 4.23%)
Mediator between maternal-specific birthweight and MI						
Hypertension	Maternal-specific birthweight	MI	-0.364 (-0.631, -0.097)	-0.582 (-0.992, -0.172)	0.275 (0.195, 0.355)	43.87% (10.31%, 77.43%)
SBP	Maternal-specific birthweight	MI	-0.364 (-0.631, -0.097)	-4.632 (-7.321, -1.943)	0.030 (0.024, 0.036)	38.39% (14.76%, 62.01%)
DBP	Maternal-specific birthweight	MI	-0.364 (-0.631, -0.097)	-2.479 (-4.431, -0.527)	0.054 (0.044, 0.064)	36.94% (7.13%, 66.76%)
Fasting insulin	Maternal-specific birthweight	MI	-0.364 (-0.631, -0.097)	-0.061 (-0.104, -0.018)	0.859 (0.459, 1.259)	14.33% (2.04%, 26.62%)
Triglycerides	Maternal-specific birthweight	MI	-0.364 (-0.631, -0.097)	-0.117 (-0.201, -0.033)	0.317 (0.205, 0.429)	10.16% (2.02%, 18.29%)
Appendicular lean mass	Maternal-specific birthweight	MI	-0.364 (-0.631, -0.097)	0.237 (0.051, 0.423)	-0.132 (-0.187, -0.077)	8.61% (0.95%, 16.27%)
Height	Maternal-specific birthweight	MI	-0.364 (-0.631, -0.097)	0.294 (0.018, 0.570)	-0.088 (-0.141, -0.035)	7.09% (0.00%, 15.01%)

Grip strength	Maternal-specific birthweight	MI	-0.364 (-0.631, -0.097)	0.051 (0.004, 0.098)	-0.359 (-0.559, -0.159)	5.06% (0.00%, 10.53%)
Mediator between fetal-specific birthweight and AF						
Height	Fetal-specific birthweight	AF	0.164 (0.023, 0.305)	0.397 (0.146, 0.648)	0.262 (0.211, 0.313)	63.48% (21.50%, 100.00%)
Appendicular lean mass	Fetal-specific birthweight	AF	0.164 (0.023, 0.305)	0.353 (0.167, 0.539)	0.242 (0.183, 0.301)	52.07% (21.89%, 82.24%)
WC	Fetal-specific birthweight	AF	0.164 (0.023, 0.305)	0.191 (0.109, 0.273)	0.352 (0.236, 0.468)	40.99% (18.87%, 63.10%)
Childhood obesity	Fetal-specific birthweight	AF	0.164 (0.023, 0.305)	0.672 (0.256, 1.088)	0.079 (0.024, 0.134)	32.41% (2.33%, 62.49%)
BMI	Fetal-specific birthweight	AF	0.164 (0.023, 0.305)	0.102 (0.043, 0.161)	0.329 (0.243, 0.415)	20.45% (7.39%, 33.50%)

Abbreviations: AF=atrial fibrillation; ApoB=Apolipoprotein B; BMI=body mass index; CI=confidence interval; DBP=diastolic blood pressure; HbA1c=glycated hemoglobin; MI=myocardial infarction; SBP=systolic blood pressure; WC=Waist circumference.

Fig. S1. Leave-one-out analysis for the associations of birthweight with MI and AF

Abbreviations: AF=atrial fibrillation; MI=myocardial infarction.

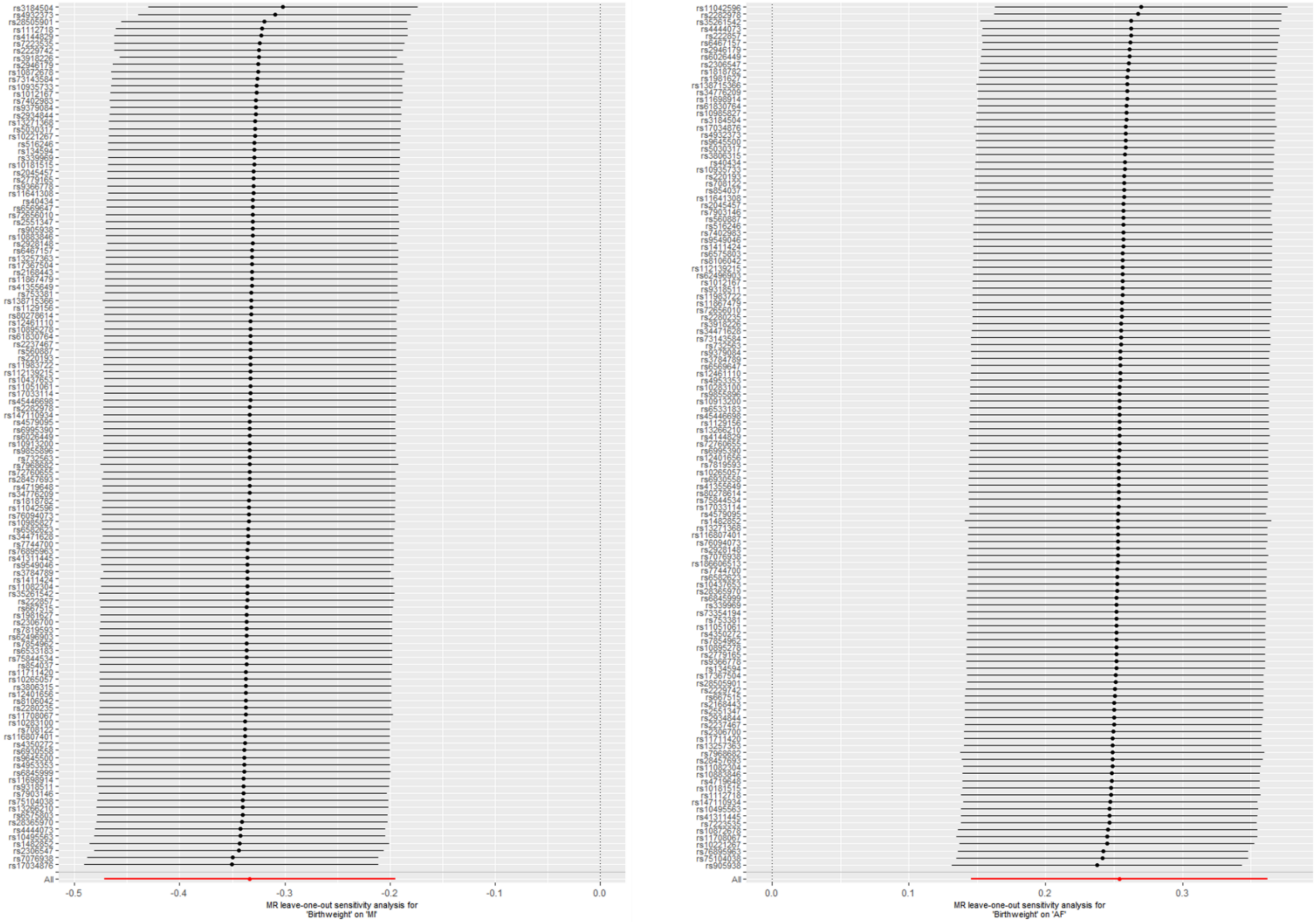

Fig. S2. Leave-one-out analysis for the associations of fetal/maternal-specific birthweight with MI and AF

Abbreviations: AF=atrial fibrillation; MI=myocardial infarction.

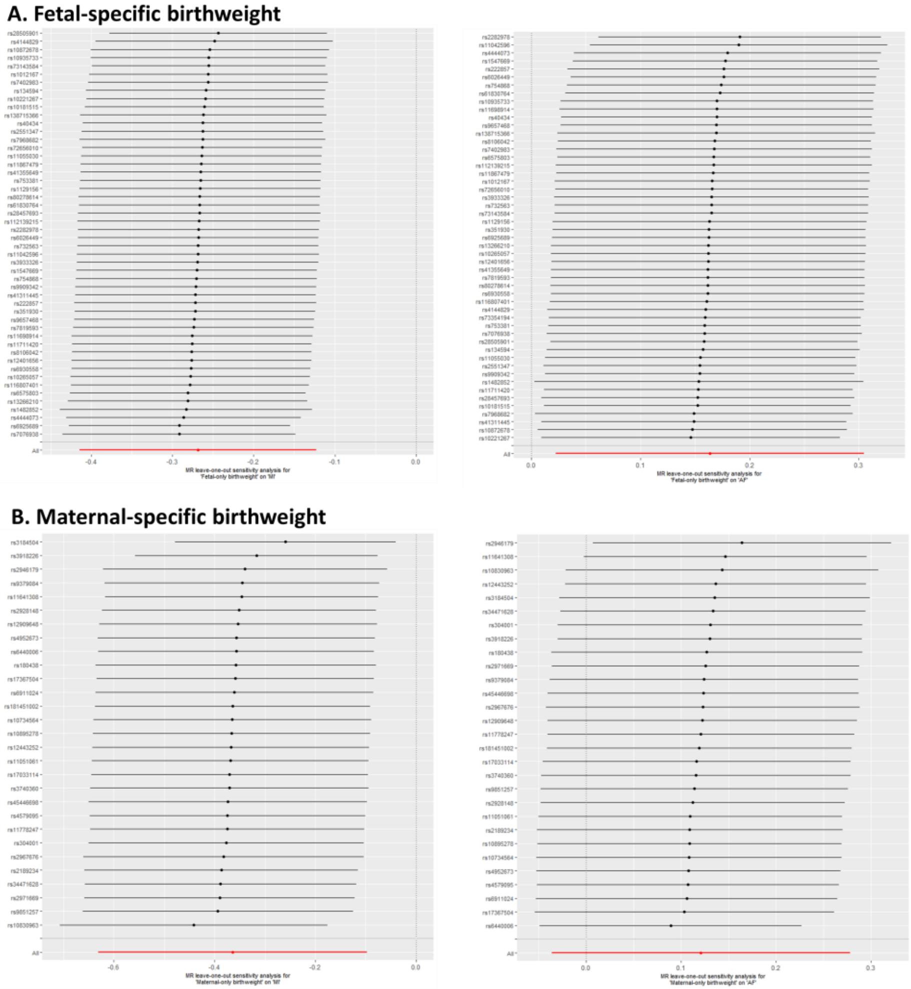

Supplement: Supplementary file 1 — Additional file 1. Additional tables and figures. [file 12933_2023_2062_MOESM1_ESM.pdf]
